# High-speed rail model reveals the gene tandem amplification mediated by short repeated sequence in eukaryote

**DOI:** 10.1038/s41598-022-06250-3

**Published:** 2022-02-10

**Authors:** Haidi Chen, Jingwen Xue, Zhenghou Zhang, Geyu Zhang, Xinyuan Xu, He Li, Ruxue Zhang, Najeeb Ullah, Lvxing Chen, Amanullah, Zhuqing Zang, Shanshan Lai, Ximiao He, Wei Li, Miao Guan, Jingyi Li, Liangbiao Chen, Cheng Deng

**Affiliations:** 1 Jiangsu Key Laboratory for Biodiversity and Biotechnology, College of Life Sciences, Nanjing Normal University, 1 Wenyuan Rd., Nanjing, 210023 China; 2 The Fourth Affiliated Hospital of China Medical University, Shenyang, 110032 China; 3 Department of Physiology, School of Basic Medicine, Tongji Medical College, Huazhong University of Science and Technology, Wuhan, 430030 Hubei China; 4 Center for Genomics and Proteomics Research, School of Basic Medicine, Tongji Medical College, Huazhong University of Science and Technology, Wuhan, 430030 Hubei China; 5 Hubei Key Laboratory of Drug Target Research and Pharmacodynamic Evaluation, Huazhong University of Science and Technology, Wuhan, 430030 Hubei China; 6 Department of Dermatovenereology, Institutes for Systems Genetics, Rare Disease Center, West China Hospital, Sichuan University, No. 37 Guo Xue Xiang Street, Chengdu, 610041 Sichuan China; 7 M.D. Department of Dermatology and Venereology, West China Hospital of Sichuan University, No. 37 Guo Xue Lane, Chengdu, 610041 China; 8 Key Laboratory of Exploration and Utilization of Aquatic Genetic Resources (Ministry of Education), Institute of Experimental Pathology, Shanghai Ocean University, Shanghai, 201306 China

**Keywords:** Evolution, Evolutionary genetics, Molecular evolution

## Abstract

The occurrence of gene duplication/amplification (GDA) provide potential material for adaptive evolution with environmental stress. Several molecular models have been proposed to explain GDA, recombination via short stretches of sequence similarity plays a crucial role. By screening genomes for such events, we propose a “SRS (short repeated sequence) *N + unit + SRS*N” amplified unit under USCE (unequal sister-chromatid exchange) for tandem amplification mediated by SRS with different repeat numbers in eukaryotes. The amplified units identified from 2131 well-organized amplification events that generate multi gene/element copy amplified with subsequent adaptive evolution in the respective species. Genomic data we analyzed showed dynamic changes among related species or subspecies or plants from different ecotypes/strains. This study clarifies the characteristics of variable copy number SRS on both sides of amplified unit under USCE mechanism, to explain well-organized gene tandem amplification under environmental stress mediated by SRS in all eukaryotes.

## Introduction

Gene duplication/amplification (GDA) is a genetic mechanism for enhanced antibiotic resistance in bacteria^1^, and the same mechanism leading to copy number variations (CNVs), which was proposed to underlie many human diseases, such as mental illness, developmental disorders and cancer in humans^2^. Several molecular mechanisms, including nonequal homologous recombination, rolling circle replication, long-distance template switching, nonhomologous end joining (NHEJ), fork stalling and template switching (FoSTeS)/microhomology-mediated break-induced replication (MMBIR) have been proposed to give rise to GDA and CNVs^2–4^. All involve sequential processes of DNA double strand breaks (DSBs) and microhomology/homology-mediated recombination followed by DNA template switching^4^. Importantly, homologous recombination, mediated by homologous sequences, plays a crucial role in DNA template switching, and thus the new junction positions created from genomic rearrangements are frequently investigated for commonalities in breakpoint junction sequences^5^. These studies showed that homologous sequences of variable lengths (from several bp to 2.7 kb) can mediate homologous recombination^1,6^. Thus far, several sequence motifs associated with recombinant hotspots have been identified in humans^7^. Earlier studies proposed a rolling circle mechanism without mediation by short repeated sequences in both eukaryotes and prokaryotes^8^. Homologous recombination can also occur via sister chromatid exchange (SCE), and unequal SCEs (USCEs), which result in the duplication or deletion of genes has been shown in several species, e.g., fly^9^, yeast^10,11^ , mouse^12,13^ and frog^14^. However, the study of USCE mechanism only studies a single gene of a specific species, only shows that homologous recombination sequences mediate USCE, or there are repeat sequences within these sequences. What are the characteristics of genes amplified under the USCE mechanism? Is the USCE mechanism widespread in all organisms? So far, these need to be further studied.

One extreme example of GDA and CNVs is the generation of tandemly arranged gene clusters, which are well-organized, locally and head-to-tail^15,16^. For instance, tens to hundreds of gene copies are produced in the histone and rDNA gene clusters, which are believed to fulfill the need for massive expression^17,18^. Additionally, type III antifreeze protein (AFPIII) exists in polar eelpouts and reaches 20–35 mg/ml in the blood of the Antarctic eelpout *Lycodichthyus dearborni*^19^. Previous screening of a genomic DNA library estimated approximately 40 similar genes^16^, which are located within a single genomic locus^16,20^. Several studies^2–4^ indicated that tandemly arranged repeats with massive gene copies could be generated by repeated sequence-mediated homologous recombination, but it is not known whether the molecular mechanisms among tandemly arranged repeats from genomes are the same.

How new genes evolve and functionally diversify are key questions in evolutionary biology, as new genes play vital roles in evolutionary innovation and allow organisms to adapt and increase in complexity and speciation^21^. An organism can acquire new genes by three distinct routes: (11) direct horizontal acquisition from other organisms (transduction, transformation and conjugation), (2) gene duplication/amplification by recombination or retrotransposition and de novo acquisition from non-coding DNA^22^. Duplication/amplification mechanisms that generate new genes or gene variants are a major force in evolution^22^. Gene duplication and subsequent modification are fundamental for genetic variability and adaptation to stresses during environmental changes. Gene duplications are grouped into 5 classes: whole-genome duplication (WGD), tandem duplication (TD), proximal duplication, transposed duplication and dispersed duplication^23^. Tandem duplicated genes account for a high proportion of eukaryotes. For example, 14–17% of genes in the human, mouse and rat genomes are duplicated genes, and nearly one-third of duplicated genes are tandemly arrayed^15^. The development of 3rd-generation sequencing technologies launched a special era for the discovery of more USCE models, enabling the detection of more USCE model sequences in the genomes of diverse organisms^24,25^.

Based on screening 568 genomes, we analyzed the sequences of 2131 USCE models and found that the characteristic of variable copy number SRS on both sides of amplification unit under USCE mechanism mined from whole genomes in nearly all taxa. We defined this amplification unit model as “SRS (short repeated sequence) *N + unit + SRS*N” structures. Unit (gene/DNA fragment under duplication) is the space between SRSs, and N (greater than or equal to 1) represents the variable copy number of SRS. The model of “SRS*N + unit + SRS*N” structure is just as high-speed rail carriage, and SRS is a connection on both sides of the carriage. Then, we proposed a high-speed rail model showing that the locally tandem amplification units are mediated by SRSs with different repeat numbers in most species.

## Results

### A high-speed rail model with an “SRS*N + unit + SRS*N” structure is universal in eukaryotes and comprises a small fraction in bacteria

**Figure 1:**
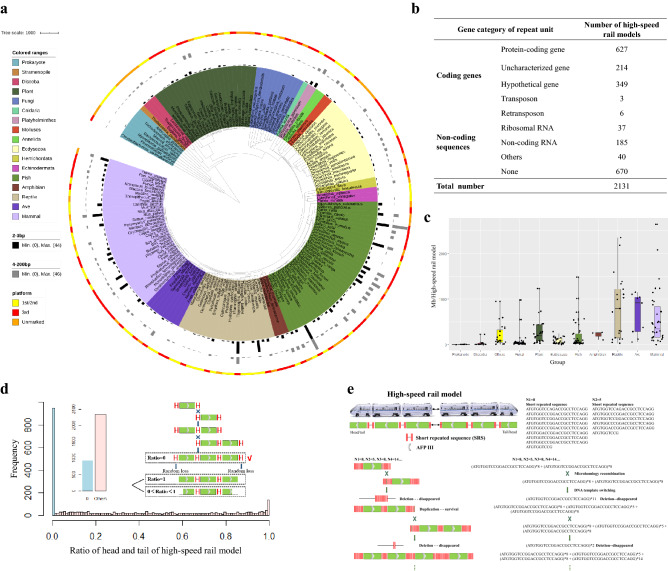
(**a**) Phylogenetic tree of 171 species with high-speed rail model structure sequences and their corresponding high-speed rail model sequence numbers with SRS lengths of 2–3 bp and 4–200 bp and their genome sequencing platforms. (**b**) Description of genes category carried in high-speed rail model sequences with SRS lengths of 2–3 bp and 4–200 bp. (**c**) The value of Mb per high-speed rail model in the genome of species with high-speed rail model sequences from different groups. The other groups contain Stramenopile, Platyhelminthes, Cnidaria, Mollusks, Annelida, Echinodermata, Hemichordata and Tunicata, which group with a small number of species. (**d**) The distribution of the ratio of the head and tail of the high-speed rail model. The ratio of 0 is the dominant value which means that the mechanism of the high-speed rail model tends to end with SRS. The case given in the box represents ratio = 0, ratio = 1 or 0 ~ 1. (**e**) Organization of the high-speed rail model structure and a proposed tandem amplification model mediated by SRS. The predicted molecular process of gene tandem amplification is resulted from SRS mediated microhomology recombination and DNA double strand break (DSB) repair. Each vertical short line represents one copy of SRS, and the ellipsis represents the variable copy number of SRS.

To explore a universal model for tandem gene amplification, we collected 568 genomes representing eukaryotic and prokaryotic species (Supplementary Table S1). Of these 568 species, 249 have “SRS*N + unit (gene/DNA fragment under duplication) + SRS*N” structure sequences, always connected head to tail by SRSs, similar to the Chinese high-speed rail model (head to tail by junction) (Fig. 1e), including 34 (out of 41) mammals, 9 (out of 11) aves, 21 (out of 47) reptiles, 3 (out of 4) amphibians, 39 (out of 41) fishes, 4 (out of 14) echinodermata, 2 (out of 2) hemichordata, 29 (out of 91) ecdysozoa, 3 (out of 7) annelida, 4 (out of 15) mollusks, 5 (out of 12) platyhelminthes, 1 (out of 14) tunicata, 3 (out of 8) cnidaria, 45 (out of 100) fungi, 25 (out of) 51 plants, 12 (out of 57) discoba, 1 (out of 1) stramenopile and 9 (out of 50) prokaryotes (Supplementary Table S2). 79 species have no information on evolutionary time, so Fig. 1a displays only 170 species in the phylogenetic tree. A total of 1025 and 1106 high-speed rail model sequences were obtained when the SRS was selected in length of 4–200 bp and 2–3 bp, respectively (Supplementary Table S2). This result indicated that the 2–3 bp SRS is similar or slightly more active in mediating amplification than the longer 4–200 bp SRS (Supplementary Fig. S1a). Interestingly, the fish group has much more high-speed rail model sequences than other groups, which may reflect the fact that the recombination rate of fish is higher than those of mammal, amphibian, and reptile^26^ (Supplementary Table S2). The median distances (in Mb) between high-speed rail models in the genome ranged from 2.66 (prokaryote group) to 912.9 (reptile group). On average, there is a high-speed rail model sequence every 250 Mb in the genomes examined (Supplementary Table S2). The presence of 2131 high-speed rail model sequences screened from 249 different species groups suggested that the high-speed rail model is a universal gene amplification model in most eukaryotic and prokaryotic species (Supplementary Table S6). Notably, the efficiency of high-speed rail model sequence screening is greatly dependent on the sequencing platform by which a genome is sequenced. First- and second-generation sequencing platforms are problematic for assembling repeated sequences^24,25,27^, while genomes sequenced through the 3rd generation sequencing platform can readily detect many high-speed rail sequences (Fig. 1a, Supplementary Fig. S1b). With the wide utilization of the 3rd generation platform, there must be more high-speed rail model sequences identified from various species.

The pattern of the “SRS*N + unit + SRS*N” model of high-speed rail sequences obtained was further analyzed. The median length of SRS ranged from 2 to 11 bp (Supplementary Fig. S2a), and the lengths of the majority of the ‘unit’ sequences were less than 5000 bp (Supplementary Fig. S2b). There was no significant difference in GC content between SRS and the host genome (Supplementary Fig. S2c and S2d and Supplementary Table S3). The medium number of all high-speed rail models identified ranged from 7 to 10 (Supplementary Fig. S2e). To confirm the high-speed rail model pattern in homologous recombination, the head/tail sequences of the high-speed rail model were collected and analyzed. 0 is defined as only SRS in head/tail without any other high-speed rail model unit sequences (“SRS*N + unit … + unit + SRS*N”), and 1 is defined as 100% unit without any SRS sequence in head/tail (“unit + SRS*N … + SRS*N + unit”, start with “unit + SRS*N” or end with “SRS*N + unit”). As shown in Fig. 1d, 0 exhibits the highest frequency ratio, and other ratios between 0 and 1 are evenly distributed. This indicates that the process of gene amplification is mostly complete. Although the total frequency of the other group (0 < ratio ≤ 1) is larger than 0 group, we speculate that this is due to the large randomness of species evolutionary process, but for a single high-speed rail model, it is still more inclined to complete replication. According to head and tail data of the high-speed rail model, we proposed that the high-speed rail model pattern is homologous recombination mediated by SRS (Fig. 1c,d).

Several sequence motifs associated with recombination hotspots have been identified in humans^28–32^. After analyzing all these high-speed rail model sequences, we identified 578 conserved motifs (“AC”, “CA”, “TG” and “GT”) from these SRSs (Supplementary Table S5). The motif “TG (AC)” was dominant with 241 high-speed rail model sequences identified to contain this motif.

### The genetic nature of the amplified units

To investigate the genetic nature of the amplified units in the high-speed rail model, these units were investigated by BLASTX and BLASTN. As shown in Supplementary Table S4, S5 and S6, from the high-speed rail model sequences we identified in this study, 1190 coding genes, including 627 protein-coding genes, 214 uncharacterized genes, 349 hypothetical genes, 3 transposons, 6 retrotransposons; and 37 ribosomal RNAs and 185 non-coding RNAs (Fig. 1b), were found to be arranged into tandem duplications. Interestingly, some of these genes matched sequences from bacteria or viruses, implying horizontal gene transfer from bacterial or viral organisms to eukaryotic organisms. An example is that the hypothetical protein gene existing in the bacteria *Staphylococcus aureus* was detected in the genome of goats, which organized into a high-speed rail model gene cluster containing 6 duplicated units (Supplementary Table S4). There are also genes known to encode RNA sequences, including pseudogene rRNA, microRNA and long non-coding RNAs. The microRNA 430a-180 genes in zebrafish were organized in the high-speed rail model. The detailed classifications of the coding capacities of the ‘unit’ sequences are listed in Supplementary Table S4 and Table S5.

### Identification of specific types of high-speed rail models

**Figure 2:**
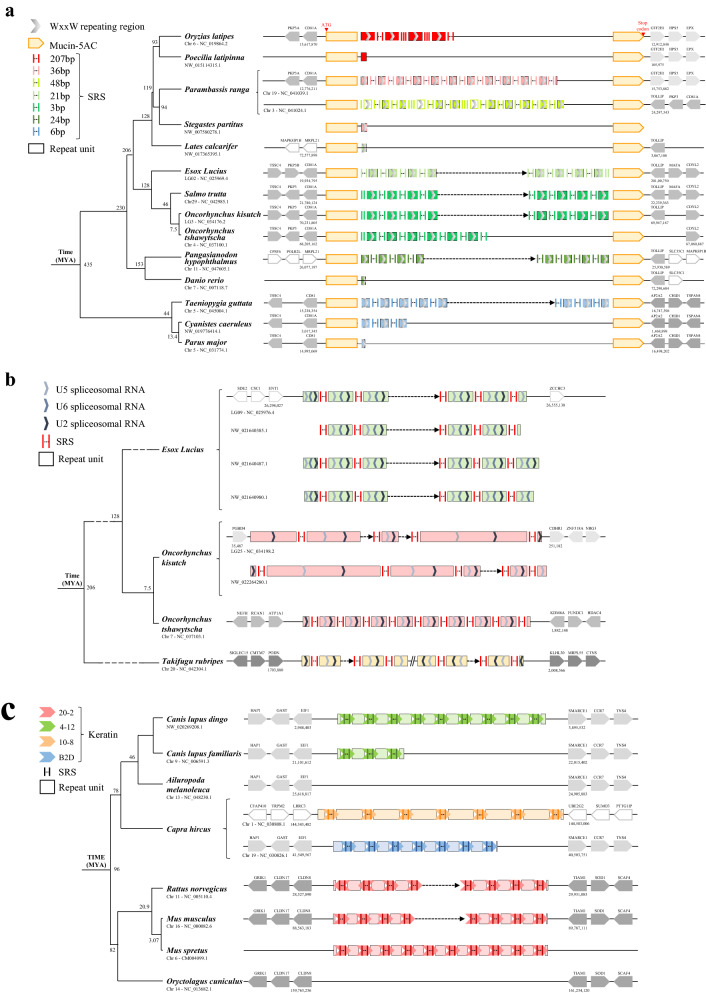
Specific types of high-speed rail structural sequences. (**a**) Tandem mucin-5AC with a WxxW repeating region. The specificity is that the whole high-speed rail model sequences is contained in mucin-5AC. Species with high-speed rail model sequences and their corresponding closed species without high-speed rail model sequence show the time when high-speed rail model appears. SRS and repeat units with different colors represent different SRS and repeat units, respectively. Repeat units with nearby colors (red and pink, different shades of green) indicated that their sequences are similar. (**b**) Tandem U2, U5 and U6 spliceosomal RNA. The specificity is that there is more than one coding gene in the repeat unit. All or two of U2, U5 and U6 spliceosomal RNAs are contained in the same repeat unit and they will be amplified by SRS at the same time. (**c**) Tandem keratin-associated protein cluster. The specificity is that SRS is involved in keratin proteins. The colors red, green, orange and blue represent keratin of 20–2, 4–12, 10–8 and B2D, respectively. Each vertical short line represents one copy of SRS, and the ellipsis represents the variable copy number of SRS.

In addition to those containing one amplifying “unit” per gene, some high-speed rail cases were more specific. In specific case 1, a high-speed rail model type of sequence is located within a domain of a protein, as exemplified by Mucin-5AC, in which numerous WxxW repeating units with unknown function were present^33^. The flanking gene of high-speed rail model from 9 species is shown in Fig. 2a. Mucin-2 and Mucin-5AC, with conserved domains but unknown functions, also found high-speed rail model sequences, as shown in supplementary Figure S3. In specific case 2, the high-speed rail model in different species has the same SRS and ‘unit’ coding gene, but there are additional sequences that are different between species exemplified in the U2, U5 and U6 clusters. These sequences could contribute to species-specific regulatory sequences. (Fig. 2b). The third special high-speed rail model case is that the SRS is part of the protein coding sequence such as the keratin-associated protein genes in *Capra hircus* (Fig. 2c).

### A case of high-speed rail genes that confer cold resistance

Antifreeze proteins (AFPs) protect various polar marine teleost fish from freezing in polar oceans^16^. Type III antifreeze protein (AFPIII) exists in polar eelpouts, reaching 20–35 mg/ml in *Lycodichthyus dearborni*^19^. Previous screening of a genomic DNA library estimated approximately 40 amplified gene copies located in a single genomic locus^16,20^ (Supplementary Fig. S4e).

**Figure 3:**
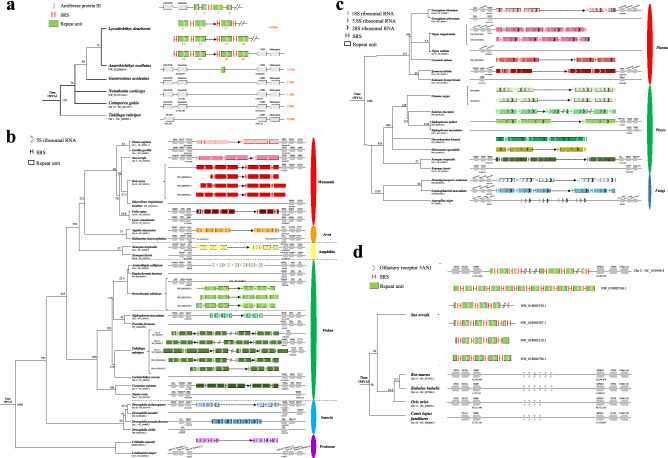
(**a**) Genomic organization of the AFPIII gene locus with tandem AFPIII 8-kb units in Antarctic eelpout (*Lycodichthys dearborni*). AFPIII genomic locus organization is conserved between *Lycodichthys dearborni* and other teleosts (*Anarrhichthys ocellatus, Gasterosteus aculeatus, Notothenia coriiceps, Cottoperca gob*i*o*, and *Takifugu rubripes*). The 320-kb locus has three large gaps with an average size of 20–30 kb and comprises ~ 30 AFPIII genes predominantly arrayed in 8-kb tandem repeats. However, for closed species of *Anarrhichthys ocellatus, Gasterosteus aculeatus, Notothenia coriiceps, Cottoperca gob*i*o*, and *Takifugu rubripes*, the gap lengths between flanking genes (synuclein and LDB3) are 5.2, 1.3, 1.5, 2.9 and 103 kb, respectively. (**b**) Tandem 5S ribosomal RNA (rRNA) cluster. Various groups of species, including mammals, aves, amphibians, fishes, insects, and protozoa, contain high-speed rail sequences containing 5S ribosomal RNA. The sequence of repeat units is different and marked with different colors. The colors of SRS and repeat units from groups of mammals, aves, amphibians, fishes, insects, and protozoa are marked with different shades of red, orange, yellow, green, blue, and purple, respectively. (**c**) Tandem 18S, 5.8S, 28S ribosomal RNA (rRNA) cluster. Various groups of species, including plants, fishes, and fungi, contain high-speed rail sequences containing 18S, 5.8S and 28S ribosomal RNA. The colors of SRS and repeat units from groups of plants, fishes, and fungi are marked with different shades of red, green, and blue, respectively. The 18S, 5.8S and 28S ribosomal RNAs are contained in the same repeat and will be amplified by SRS at the same time. (**d**) Tandem OR5AN1-like clusters in wild *Sus scrofa* related to musk-smelling compound. There are several loci with high-speed rail sequences (OR5AN1-like) in *Sus scrofa*. However, there are no high-speed rail structural sequences in closed species (*Bos taurus, Bubalus bubalis, Ovis aries*, and *Canis lupus familiaris*). Each vertical short line represents one copy of SRS, and the ellipsis represents the variable copy number of SRS.

By screening a previously constructed bacterial artificial chromosome (BAC) library of *L. dearborni* genomic DNA, sequencing, and subsequent assembly (see Supplementary Method and Supplementary Fig. S4), we reconstructed the *L. dearborni* AFPIII locus spanning approximately 400 kb of the genomic region with three gaps of an average length of 20–30 kb. We annotated 40 8 kb AFPIII-containing units arranged in tandem within the AFPIII locus, and identified 30 intact ORFs (Fig. 3a). The 8 kb repeating units are flanked by different numbers of SRS “ATGTGGCCCGGACCGCCTCCAGG”, and the repeated sequences shared more than 95% sequence similarity between each other (Supplementary Fig. S5). A few of the repeating units contained retrotransposon insertions in the non-coding region (see the AFPIII-15 unit in Supplementary Fig. S6). The entire locus is flanked by Glud1b-Synuclein in the 5’ end and LIM domain binding 3b-Melanopsin in the 3’ end. The Glud1b-synuclein-LIM domain binding 3b-melanopsin synteny is conserved in other teleosts. However, the locus between synuclein-LIM domain binding 3b in stickleback occupies only 1.3 kb without an AFPIII homologous sequence (Fig. 3a).

From the 40 repeating structures, we identified 30 contained intact ORFs. All 30 repeating units contained one AFPIII coding gene, consistent with the ‘one unit one gene’ high-speed rail model. Phylogenetic relationship analysis of the 30 genes showed two major lineages (Supplementary Fig. S7a). Among these genes, the number of ice-binding domains (IDBs) coded in each gene may differ. The majority (23) of AFPIII genes encode proteins with only one IBD (termed LD1). In 4 genes however, the number of IBDs encoded within each gene differ from that of LD1, of which two encode AFPIII molecules containing 2 IBDs (LD2), one encodes 3 IDBs (LD3) and one encodes 4 IDBs (LD4) (Supplementary Fig. S7b). In the multi-IDB encoding genes, the individual ice-binding domains are highly identical to those of LD1 in amino acid sequence, except 2, 3, or 4 IDBs are linked by a conserved 9-amino-acid linker, suggesting intragenic domain duplication, like the WxxW types of duplication in Mucin-5AC genes shown in the previous case.

Evolutionary analysis by PAML showed that the majority of AFPIII genes are under purifying selection. For example, AFPIII-2, 5, and 7 are 100% identical in nucleotides (Supplementary Fig. S7a). Adaptive evolution is also detected on paralogs derived from gene amplification. Seven AFPIIIs were under significant positive selection (Supplementary Fig. S7c), including the multimer AFPIII genes. The higher hysteresis activity found in the multi-IDB AFPs^34^ indicated adaptative evolution to the more extreme freezing conditions in the Antarctic environment. The high-speed rail model of the AFPIII gene locus represents an example of gene amplification under environmental stress in vertebrates.

### The high-speed rail model mediates housekeeping gene amplification-tandem 5S rRNA clusters and tandem 18S-5.8S-28S rRNA clusters

Ribosomal genes are repeated and organized in two distinct clusters in eukaryotic genomes (45S rDNA and 5S rDNA) located in a single locus or on multiple loci. Three types of rRNA molecules (18S, 5.8S, and 28S) are produced by posttranscriptional processing of a 45S precursor transcript in eukaryote. To produce sufficient rRNA for the highly abundant ribosomes that are indispensable in translation, the genes encoding the rRNA are represented in multiple copies in eukaryotic genomes^35^. As shown in Fig. 3b,c, the high-speed rail model exhibits gene amplification of rDNA in various species.

High-speed rail models containing 5S ribosomal RNA coding sequences were amply found in 13 species, including Mammals *(Homo sapiens, Sus scrofa, Ovis aries* and *Felis catus),* Aves *(Aquila chrysaetos),* Amphibians *(Xenopus tropicalis),* Fishes *(Astatotilapia calliptera, Oreochromis niloticus, Xiphophorus maculatus, Takifugu flavidus* and *Carassius auratus)*, Insetca *(Drosopila melanogaster* and *Drosopila pseudoobscura)* and Protozoa (*Crithidia expoeki*) (Fig. 3b). Amplification of 5S rRNA genes in 5 species (*Sus scrofa, Aquila chrysaetos, Xiphophorus maculatus, Takifugu rubripes* and *Drosophila melanogaster*) was restricted to the high-speed rail model. However, amplification of 5S rRNA genes in another 6 species (*Homo sapiens, Xenopus tropicalis, Astatotilapia calliptera, Oreochromis niloticus, Carassius auratus* and *Drosophila pseudoobscura*) adopted both the high-speed rail model and other duplication mechanisms (Supplementary Table S7). The 5S rDNA transcriptional unit consists of a 120-bp fragment that is evolutionarily conserved, even between phylogenetically distant organisms such as *Homo sapiens* and *Xenopus tropicalis*, and a nontranscribed spacer (NTS) subject to sequence variations in size and/or sequence^36^. The units and the SRS flanking the units bear no sequence similarity among the 13 high-speed rail models, except for the 5S rRNA coding regions within these units of the high-speed rail model, which showed high similarity, and this result indicated that the 5S rRNA clusters from 13 different species were independently derived by the high-speed rail model (Fig. 3b). In addition, the 5S rRNA from 13 species also showed local tandem amplification, e.g., rolling circle model^8^ or other discrete duplicate mechanisms, which is different from the high-speed rail model (Fig. 3b and Supplementary Fig. S8). The 13 5S rRNA high-speed rail models were distributed in different chromosomal locations and had distinct flanking genes (Fig. 3b), suggesting independent origins.

The repeat high-speed rail model containing 18S, 5.8S, 28S rRNA was also found in plants (*Gossypium hirsutum, Vigna unguiculata, Cucumis sativus* and *Ipomoea triloba*), fishes (*Channa argus, Salarias fasciatus, Xiphophorus helleri, Oncorhynchus kisutch, Etheostoma spectabile* and *Xenopus tropicalis*) and fungi (*Parastagonospora nodorum* and *Leptosphaeria maculans*) (Fig. 3c). Similar to the 5S rRNA high-speed rail model described above, the SRSs and the units of the 12 high-speed rail s are different, again supporting independent origins of the 18S, 5.8S and 28S rRNA high-speed rail models in different species.

Thus, rDNA amplification in diverse taxa could have taken place in different molecular mechanisms, but the high-speed rail model appears to be the major model.

### The high-speed rail model of amplification mediates expansion of the olfactory receptor 5AN1 clusters

The olfactory receptor family 5 subfamily AN number 1 (OR5AN1) is a G protein-coupled receptors (GPCR), which recognize musk and its related molecules^37^. We found that the organization of the OR5AN1 genes followed the high-speed rail model in *Sus scrofa,* in which tandem repeat units encoding OR5AN1 are flanked by SRSs of “CCTT”. The same chromosomal loci in other species encode OR5AN1 as well but are not organized in the high-speed rail model (Fig. 3d). In general, pigs have more copies of functional OR5AN1 copies than other species. It is likely that the high-speed rail model of amplification resulted in further expansion of OR5AN1 in pigs which rendered pigs with higher musk sensitivity. We found that the length heterogeneity of pig OR5AN1 copies was due to variable copies of these SRSs. Meanwhile, both *Bos taurus* and *Bubalus bubalis* have 6 copies of OR5AN1 gene, *Ovis aries* and *Canis lupus familiaris* both have 4 copies of OR5AN1 gene, and none of them are amplified in an SRS-mediated manner.

### Dynamic unit numbers among closely related species, subspecies, and ecotypes/strains

**Figure 4:**
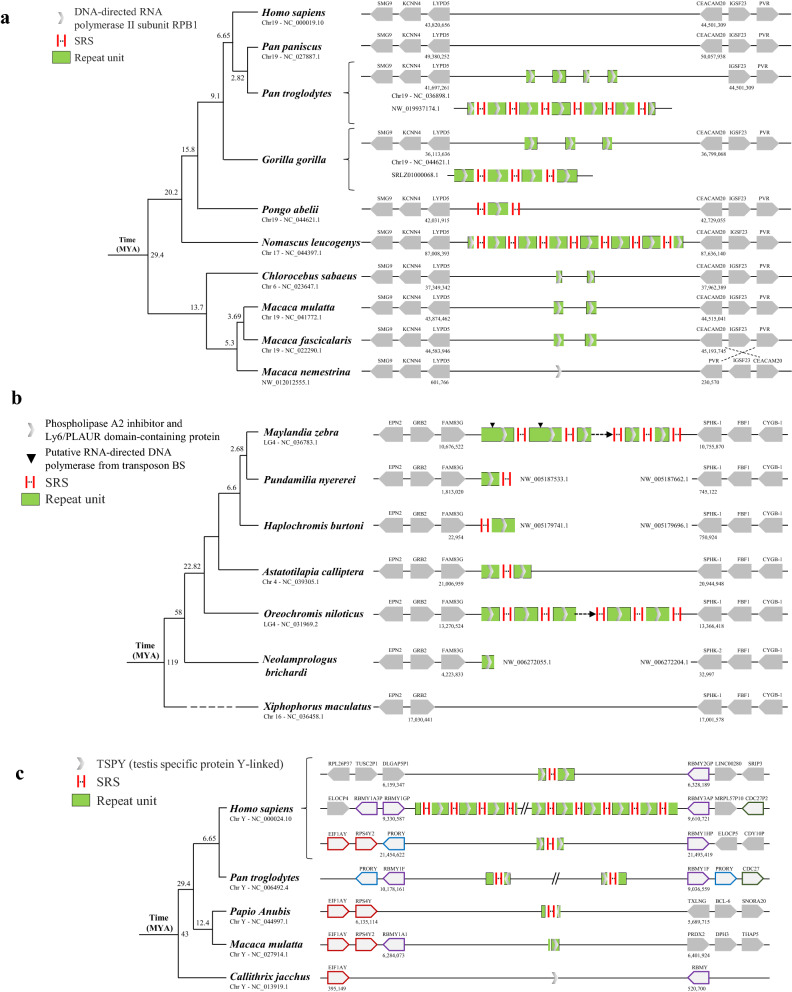
The high-speed rail model shows dynamic unit numbers among closed species. (**a**) The species *Pan troglodytes, Gorilla gorilla, Pongo abelii*, and *Nomascus leucogenys* all have high-speed rail model sequences that contain repeat units coding the DNA-directed RNA polymerase II subunit RPB1. Their closed species (*Chlorocebus sabaeus, Macaca mulatta, Macaca fascicalaris*, and *Macaca nemestrina*) do not have complete high-speed rail model sequences. (**b**) The species *Maylandia zebra, Pundamilia nyererei, Haplochromis burtoni, Astatotilapia calliptera*, and *Oreochromis niloticus* all have high-speed rail sequences that contain repeat units coding phospholipase A2 inhibitor and Ly6/PLAUR domain-containing proteins. Their closed species (*Neolamprologus brichardi* and *Xiphophorus maculatus*) do not have complete high-speed rail model sequences. There are two insert sequences (putative RNA-directed DNA polymerase from transposon BS) in the first and second repeat units of *Maylandia zebra* and marked with black triangles. (**c**) The species *Homo sapiens*, *Pan troglodytes*, and *Papio Anubis* all have high-speed rail model sequences that contain repeat units coding testis-specific protein Y-linked (TSPY). Their closed species (*Macaca mulatta* and *Callithrix jacchus*) do not have complete high-speed rail model sequences. Each vertical short line represents one copy of SRS, and the ellipsis represents the variable copy number of SRS.

The high-speed rail model of gene amplification was found to be present in four primates (*Pan troglodytes, Gorilla gorilla, Pongo abelii* and *Nomascus leucogenys*), but not in another 6 species, although all the species shared the same locus (Fig. 4a). Uniform high-speed rail sequences with the same SRS and same unit sequences were found in the DNA-directed RNA polymerase II subunit RPB1 gene clusters of the primates (Fig. 4a), the phospholipase A2 inhibitor and the Ly6/PLAUR domain-containing protein clusters in fishes (Fig. 4b) and the TSPY cluster in primates (Fig. 4c). The dynamics of the loci were observed in those species (Fig. 4). The numbers of repeated units ranged from 1 to 10, and those of SRS varied. Furthermore, the lengths of the head/tail units were also different. The interspecific variations of the same high-speed rail model indicated the high evolutionary dynamics of the high-speed rail models.

### High-speed rail model dynamics are also observed among subspecies

**Figure 5:**
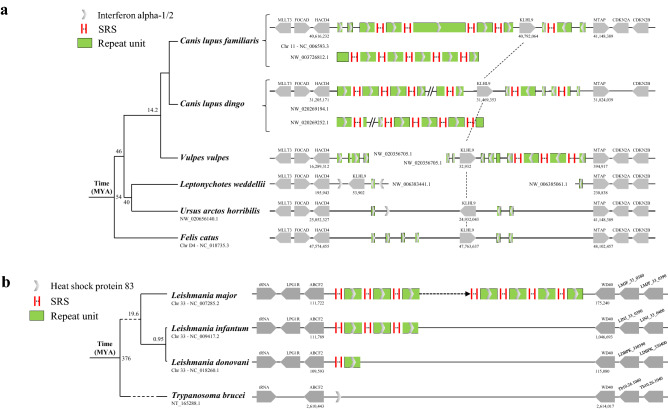
The high-speed rail model shows dynamic unit numbers among subspecies. (**a**) The subspecies of *Canis lupus familiaris* and *Canis lupus dingo* have high-speed rail model sequences whose repeat unit encodes interferon alpha-1/2. (**b**) The subspecies of *Leishmania major*, *Leishmania infantum* and *Leishmania donovani* all have high-speed rail model sequences whose repeat unit encodes heat shock protein 83. Each vertical short line represents one copy of SRS, and the ellipsis represents the variable copy number of SRS.

Through genome screening, we found that the IFNα-1/2 gene locus is consists of tandem repeat 3.6-kb units (one IFNα-1/2 gene per unit) in subspecies of *Canis lupus familiaris* and *Canis lupus dingo* which belong to *Canis lupus*. They shared the same repeat units and SRS (Fig. 5a), suggesting the same high-speed rail model. Sequence analysis showed that this high-speed rail type of locus first appeared in *Vulpes vulpes* but not in *Leptonychotes weddellii*. We hypothesize that this high-speed rail model experienced dynamic changes in *Canis lupus familiaris* and *Canis lupus dingo* (Fig. 5a).

Interferon (IFN) is a glycoprotein, which has the antiviral functions inhibiting cell proliferation and regulating immunity and antitumor activity1^38,39^. As shown in Supplementary Figure S9, type I IFNs in the skin of FV3-challenged (Frog virus 3, now recognized worldwide to be an amphibian pathogen with a threatening potential to cross multiple species barriers^40^) amphibians *Xenopus laevis* and *Xenopus tropicalis* were arranged by the high-speed rail model. Subcutaneous administration of type I IFN offered short-term protection of tadpoles against FV3, and these type I IFNs induced the expression of distinct antiviral genes in the tadpole skin^41^.

Similarly, the heat shock protein 83 locus in the subspecies of *Leishmania major* was also found to be highly dynamic (Fig. 5b).

### High-speed rail model genes show dynamics among different Arabidopsis thaliana ecotypes and Drosophila melanogaster strains

**Figure 6:**
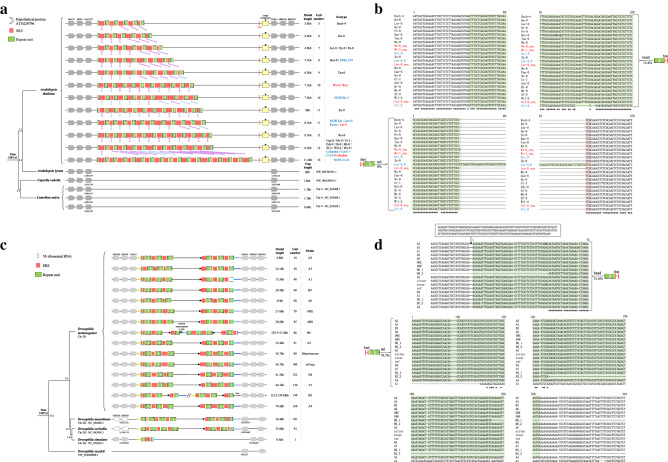
The high-speed rail model shows dynamic unit numbers among ecotypes or strains. (**a**) Dynamic unit numbers in different ecotypes of *Arabidopsis thaliana*. The names of different ecotypes with black, blue, and red colors are genomes from references of Nature, NCBI and our PCR and third-generation sequencing experiments, respectively. Arrows pointing to different ecotypes represent consistent copy numbers of SRS. The yellow box represents the TNP2 transposon. (**b**) Alignment of the heads and tails of different ecotypes of *Arabidopsis thaliana*. The sequences of repeat units from the head and tail of the high-speed rail model sequence and SRS are marked with red and green colors, respectively. (**c**) Dynamic unit numbers in strains of *Drosopila melanogaster*. (**d**) Alignment of the heads and tails of different strains of *Drosopila melanogaster*. The sequences of repeat units are marked in green colors. Each vertical short line represents one copy of SRS, and the ellipsis represents the variable copy number of SRS.

Genome sequences (Illumina sequencing platform) from 19 *Arabidopsis thaliana* ecotypes were obtained from the literature^42^. The dynamic amplification units between ecotypes are shown in Fig. 6a,b. The number of units in the high-speed rail models from the same loci are different among subspecies of Kn-0 (Lithuania), Ler-0 (Poland, formerly Germany), Bur-0 (Ireland), Tsu-0 (Japan), Zu-0 (Germany) and Can-0 (Canary Isles). The high-speed rail models from some of the ecotypes are identical, e.g., Ler-0, Oy-0 and Po-0 sharing a same high-speed rail model of 7 units, and the ecotypes of Can-0, Ct-1, Edi-0, Hi-0, Mt-0, No-0, Sf-2, Wil-2, Ws-0 and Wu-0 share the same high-speed rail model of 12 units (Fig. 6a and Supplementary Table S8). The evolution of the high-speed rail model of gene duplication in these *Arabidopsis thaliana* ecotypes could be traced (Fig. 6a), although they showed different numbers of units and SRSs. The sequences at the head and tail of the high-speed rail model are highly conserved (Fig. 6b). To confirm the dynamic unit numbers among the ecotypes of *Arabidopsis thaliana*, we performed long-range PCR to amplify and sequence these high-speed rail model loci. We found that the sequencing results of ecotype Ws-0 and Ler-0 were consistent with those of NCBI (Fig. 6a and Supplementary Fig. S10).

Similar results were found in *Drosophila melanogaster* strains. In the 18 tested strains, the number of units ranged from 16 to 166 and the whole length of the high-speed rail model structure sequence was in the range from 4.8 to 52 kb (Fig. 6c and Supplementary Table S9). The head and tail of the high-speed rail model structure sequence are conserved between different subspecies (Fig. 6d). Rick et al. and Alan M. et al. also showed that CNVs varied across subspecies and even individuals^43,44^. The sequences at the head and tail of the high-speed rail model sequences and the sequences flanking the high-speed rail model sequence in different strains of *Drosophila melanogaster* and different ecotypes of *Arabidopsis thaliana* are highly conserved (Fig. 6b,d). Taken together, the high-speed rail model shows dynamic unit numbers between closely related species, subspecies, ecotypes and strains of the same species.

## Discussion

**Figure 7:**
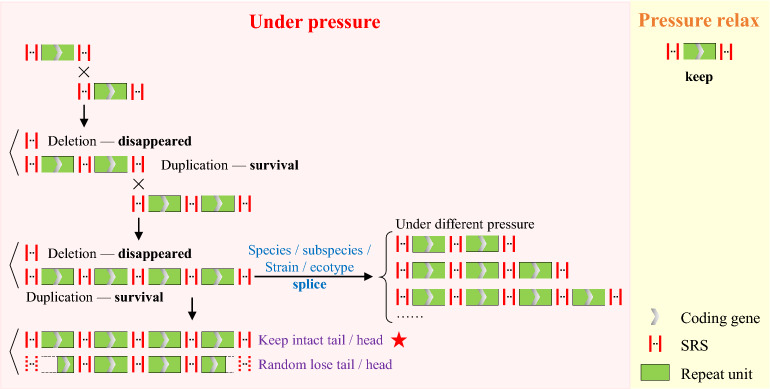
The high-speed rail model shows differences in the SRS and repeat units under different pressure. Each vertical short line represents one copy of SRS, and the ellipsis represents the variable copy number of SRS.

A comparison of all gene clusters mediated by the high-speed rail model to date revealed that the high-speed rail model showed dynamics among different species/subspecies and strains. Moreover, their gene-coding sequences were characterized by high variation. Consequently, the differences in the number of copies of SRS and repeat units were most likely responsible for genetic adaptation to altered growth conditions or environmental stresses (Fig. 7). As previously studied, gene duplication amplification (GDA) is an expatiation/adaptation in response to changes in the environment^23,45,46^. Organisms need continuous genetic variations to adapt to the environment. Copy number variation (CNV) is a type of structural variant involving alterations in the number of copies of DNA^43^. The term CNV is used to describe all kinds of variations in the genome, including tandem genome repeats, duplication, and deletion^5^. CNV can be simple in structure, such as tandem gene duplication or may involve complex gains or losses of homologous sequences at multiple sites in the genome. CNVs influence gene expression, phenotype variability and adaptation by varying gene dosages and providing genetic materials of evolution.

Several studies^2–4^ have indicated that tandemly arranged repeats with massive gene copies could be generated by repeated sequence-mediated homology recombination, but it is not known whether the molecular mechanisms among tandemly arranged repeats are the same. Here, we identified a high-speed rail model for tandem amplification mediated by SRSs with different repeat numbers in eukaryotes by screening 568 genomes. Amplified units from high-speed rail model encode various kinds of coding or non-coding sequences and increase gene dosage or generate gene diversity leading to adaptive evolution. The high-speed rail model shows dynamic unit numbers among closed species, subspecies, and ecotypes/strains, but not among individuals from the same ecotypes/strains (Supplementary Fig. S11). In conclusion, our study provides a special high-speed rail model, different from the rolling cycle model, to explain well-organized gene tandem amplification under environmental stress mediated by SRSs in all eukaryotes. The length heterogeneity of amplified units is due to variable copies of these SRSs.

## Methods

### Screening the patterns of the high-speed rail model in genomes

A total of 568 genomes representing all classic species of eukaryotes and prokaryotes were downloaded from the GenBank database. The reference genome of each species was chosen. Tandem repeat sequences from the whole genome were screened by Tandem Repeats Finder software^47^ (version 4.09, match, mismatch and indel with the parameters of 2, 3 and 5) with the Linux system. A matrix of all short-repeated sequences (SRSs) with their information (start, end, copy number, percentage match, SRS) was derived from TRF. The program algorithm based on R language (version 4.0.3) was built with several screening conditions to screen all high-speed rail models. The set screening conditions contained the following: (1) the length of SRS was chosen as 2–200 bp; (2) the match between SRSs was larger than 70%; (3) the length of the amplified unit was 50–30,000 bp; (4) the nucleotide sequence similarity between units was larger than 90% through two sequence BLAST; and (5) the copy number of each SRS was inconsistent (If the copy number of each SRS is same, we will filter out these sequences). The coding sequence (protein-coding gene, uncharacterized gene, and hypothetical gene) and non-coding sequence (transposons, retrotransposon, rRNA, pseudogenes rRNA, microRNA, long non-coding sequence) of the amplified unit were investigated by BLASTX and BLASTN.

### Genome PCR and third generation sequencing

All the experimental materials were complied with Regulation for the collection of genetic resources (This guideline was formulated by the Ministry of environmental protection of China and implemented in 2012). Plants should be collected according to the principle of non-injurious sampling of animals and fresh leaves tissues can be collected. All material was frozen in liquid nitrogen and stored at − 80 °C before processing. High-speed rail models with different *Arabidopsis thaliana* ecotypes (Ler-0, Ws-0, Col-0 and Ws-1 wild-type) were chosen for third-generation resequencing. Library preparation was performed following the manufacturer’s instructions. Approximately 100 mg (1 cm^2^) of greenhouse-grown *Arabidopsis thaliana* leaves (the frozen leaves as a gift from Professor Ziqiang Zhu and Professor Weifeng Xu, the method of leaves tissues collection follows that described by Schmid et al.^48^) and frozen muscle tissues of *L. dearborni* (the dead fish tissues as a gift from Professor Liangbiao Chen) was used for genomic DNA extraction. Briefly, tissues were incubated with 500 μl of homogenization buffer (0.4 M NaCl, 10 mM Tris–HCl pH 8.0, 2 mM EDTA pH 8.0, and 400 μg/ml proteinase K) at 55 °C overnight. Samples were spun down for 10 min at 12,000 g, and an equal volume of isopropanol was added to the supernatant. Samples were incubated at -20 °C for 1 h and then centrifuged for 15 min at 4 °C and 12,000 g. The pellet was washed with 75% ethanol, dried and finally resuspended in 100 µl sterile dH_2_O. Genomic DNA was amplified using a long PCR kit (M0533S, NEB)^49^. Then the products were collected for sequencing (PacBio Sequel II, CCS model).

### Gene synteny analyses of 5S rRNA and other genes locus

For the identification of 5S rRNA and other genes in other species, all annotated 5S rRNA and other genes from the GenBank database were checked for their series type. The divergence time of high-speed rail model-containing genes compared with nearest species were estimated using the real-time method, which has been shown to perform well in the calculation of divergence times for duplicated genes.

### Phylogenetic analyses

The sequence of the high-speed rail model was subjected to BLASTX and BLASTN (https://blast.ncbi.nlm.nih.gov) analysis to confirm the gene encoded in the repeat unit, and other species with the same or similar high-speed rail model sequence were also found. Then, these species were checked until the related species without the high-speed rail model were found. When no species with the same high-speed rail model were found in the first step, Genome Data Viewer (GDV) (https://www.ncbi.nlm.nih.gov/genome/gdv) was used to find related species. The genomes of these species were subjected to BLAST to determine whether they had the same model. After finding the flanking genes of the high-speed rail model, the sequences between the same flanking genes of relative species were analyzed to confirm once again whether there were similar sequences. TIMETREE^50,51^ (www.timetree.org) was used to determine the divergence time of the species, and to infer the generation time of the high-speed rail model. Tandem unit and AFPIII coding sequences were aligned by Clustal-W version 1.83 with default settings^52^. Phylogenetic trees were constructed using the neighbor-joining (NJ) algorithm with 1000 bootstrap replicates in MEGA version 4^53^.

### Sequence analysis of head/tail sequence

We used BLAST to confirm the head/tail (we artificially set a direction for each high-speed rail and marked head in the front and tail in the tail, and head means the first unit, tail means the last unit) sequence of the high-speed rail model and then calculated the sequence integrity (shown as a percentage of the complete repeat unit sequence length). 0 to 1 indicates the ratio of the length of the head/tail unit sequence to the length of the complete unit sequence in this high-speed rail. “1” is defined as 100% unit without any SRS sequence in head/tail, “0” is defined as only SRS in head/tail without any other high-speed rail model unit sequences, “0–1” means that this is an incomplete unit. To confirm whether the head/tail sequences of different strains were consistent, we aligned (CLUSTALX)^54^ their head/tail and nearby sequences. According to the length of the high-speed rail model of each strain, the numbers of “N” were arranged into a matrix, to analyze the model structure of different strains. Global and complete alignments were used to match the numbers, and the matched numbers were marked with the same color to show the difference more intuitively in conservation between the head/tail and the middle part of the model.

### AFPIII gene locus assembly

BAC library construction and screening have been previously described^20^. Six AFPIII clones that covered most of the AFPIII locus were sequenced by shotgun library sequencing technology (Supplementary Method). Gene annotation were obtained by BLAST, and the contigs were compared with the National Center for Biotechnology Information (NCBI) database.

### Chromosomal fluorescence in situ hybridization of AFPIII

The full-length digoxigenin-labeled AFPIII gene probe was hybridized to metaphase chromosomal preparations from *L. dearborni* kidney cells following a previously published protocol^55^.

### Selection pressure analysis of AFPIIIs

A wrapper tool named EasycodeML^56^ were used for AFPIIIs selection pressure analysis. Branch site model (BSM) can be used to identify signals of episodic selection occurring along a specified branch after gene duplication^57^. The output of the BSM model is a table shown the model, Ln L, estimates of parameters and p-value.

## Supplementary Information

Supplementary Information 1.

Supplementary Information 2.

## Data Availability

The sequences reported in this paper have been deposited in the GenBank database (LdBAC001 accession no. JX844826, LdBAC003 accession no. JX844828, LdBAC005-004 accession no. JX844827, and LdBAC007 accession no. JX844825). All sequences used in data analysis are available on NCBI at https://www.ncbi.nlm.nih.gov/ and UCL at http://mtweb.cs.ucl.ac.uk/mus/www/19genomes/19genomes.htm.
