# Supplementary material for: High-speed rail model reveals the gene tandem amplification mediated by short repeated sequence in eukaryote
Source: Sci Rep. 2022 Feb 10;12:2289. doi: 10.1038/s41598-022-06250-3 (PMC8831618; doi:10.1038/s41598-022-06250-3)
Supplement: Supplementary file 1 — Supplementary Information 1. [file 41598_2022_6250_MOESM1_ESM.docx]

**High-speed Rail Model Reveals the Gene Tandem Amplification Mediated by Short Repeated Sequence in Eukaryote**

Haidi Chen^1#^, Jingwen Xue^1#^, Zhenghou Zhang^2#^, Geyu Zhang^1#^, Xinyuan Xu^1^, He Li^1^, Ruxue Zhang^1^, Najeeb Ullah^1^, Lvxing Chen^1^, Amanullah^1^, Zhuqing Zang^1^, Shanshan Lai^1^, Ximiao He^3,4,5^, Wei Li^6^, Miao Guan^1,*^, Jingyi Li^7,*^, Liangbiao Chen^8,*^, Cheng Deng^1,*^

^1^Jiangsu Key Laboratory for Biodiversity and Biotechnology, College of Life Sciences, Nanjing Normal University, Nanjing 210023, China.

^2^The Fourth Affiliated Hospital of China Medical University, Shenyang 110032, China.

^3^Department of Physiology, School of Basic Medicine, Tongji Medical College, Huazhong University of Science and Technology, Wuhan 430030, Hubei, China.

^4^Center for Genomics and Proteomics Research, School of Basic Medicine, Tongji Medical College, Huazhong University of Science and Technology, Wuhan 430030, Hubei, China.

^5^Hubei Key Laboratory of Drug Target Research and Pharmacodynamic Evaluation, Huazhong University of Science and Technology, Wuhan 430030, Hubei, China.

^6^Department of Dermatovenereology, Rare Disease Center, West China Hospital, Sichuan University, No. 37 Guo Xue Xiang Street, Chengdu, Sichuan 610041, China.

^7^M.D. Department of Dermatology and Venereology, West China Hospital of Sichuan University, No.37 Guo Xue Lane, Chengdu, 610041, China.

^8^Key Laboratory of Exploration and Utilization of Aquatic Genetic Resources (Ministry of Education), Shanghai Ocean University, Shanghai, 201306, China.

*Corresponding author: Jiangsu Key Laboratory for Biodiversity and Biotechnology, College of Life Sciences, Nanjing Normal University, Nanjing 210023, China. 1 Wenyuan Rd. Nanjing 210023, China. Tel.: 86-25-8589-1040; E-mail: [dengcheng2014@126.com](mailto:dengcheng@njnu.edu.cnI); Institutes for Systems Genetics, West China Hospital, Sichuan University, Chengdu, 610041, China. Institute of Experimental Pathology. Key Laboratory of Exploration and Utilization of Aquatic Genetic Resources (Ministry of Education), Shanghai Ocean University, Shanghai, zip code 201306, China. [lbchen@shou.edu.cn](mailto:lbchen@shou.edu.cn); Jiangsu Key Laboratory for Biodiversity and Biotechnology, College of Life Sciences, Nanjing Normal University, Nanjing 210023, China. 1 Wenyuan Rd. Nanjing 210023, China. Tel.: 86-25-8589-1040; E-mail: [xiaoniao8911@126.com](mailto:xiaoniao8911@126.com); M.D. Department of Dermatology and Venereology, West China Hospital of Sichuan University, No.37 Guo Xue Lane, Chengdu, 610041, China. E-mail: ljy7733@163.com

**Supplementary Figure S1a.** The counts of different groups of short repeated sequences (SRS) with different bp.


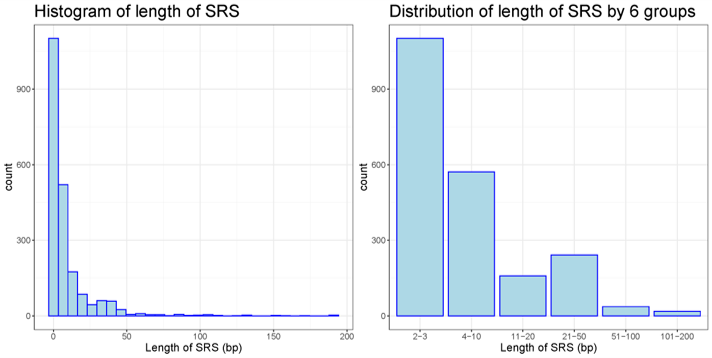


**Supplementary Figure S1b.** The distances (in Mb) between high-speed rail models in the genome from species group of prokaryotes, discoba, fungi, plant, ecdysozoa, fish, reptile and mammal using different sequencing platforms (1^st^/2^nd^, 3^rd^ and unmarked). The distances (in Mb) between high-speed rail models in the genome using sequencing platform of 3^rd^ is smaller than 1^st^/2rd for most of species’ groups which demonstrated 3^rd^ sequencing platform can discovery more high-speed rail model sequences than other platforms.

**
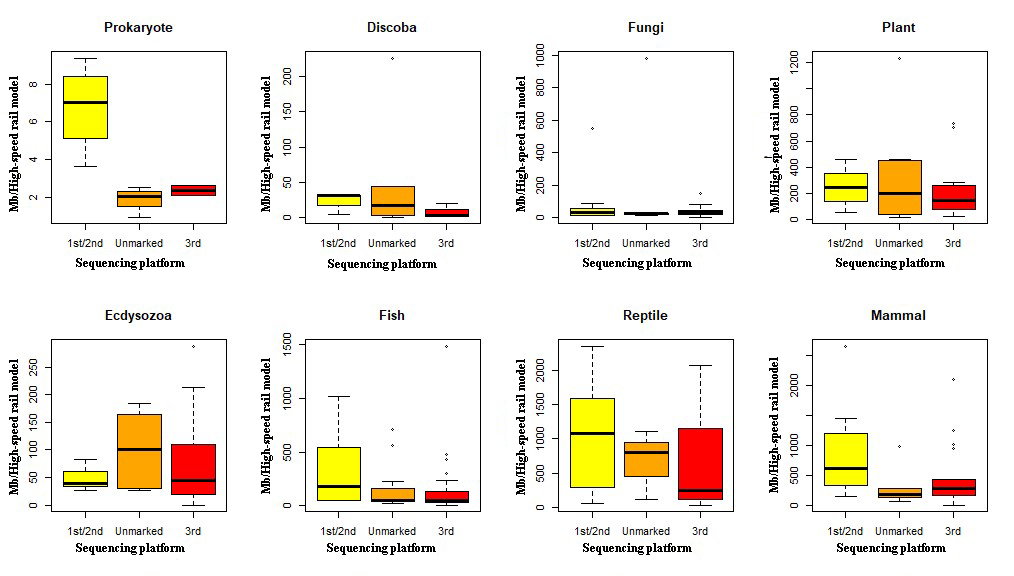
**

**Supplementary Figure S2.** (a) Summary of the distribution of length of simple repeats (bp); (b) distribution of length of unit (bp); (c) distribution of GC content of genome; (d) distribution of GC content of simple repeats; (e) distribution of unit number of six species groups (fungi, plant, ecdysozoa, fish, amphibian, reptile, ave and mammal).

**
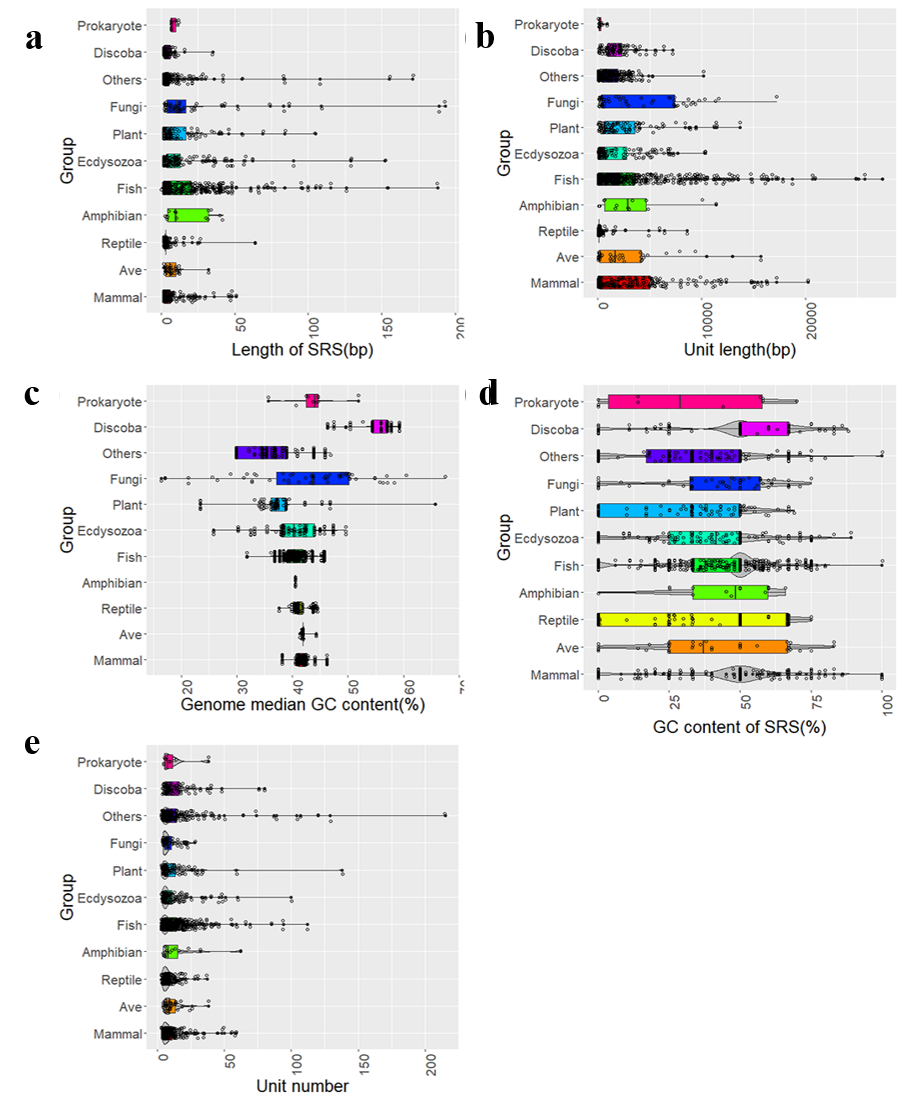
**

**Supplementary Figure S3.** Specific types of high-speed rail model structural sequences. (a) Tandem mucin-2 with a WxxW repeating region. (b) Tandem mucin-2/5AC with an unknown domain repeating region. Each vertical short line represents an SRS, and the ellipsis represents the variable copy number of SRS. SRS and repeat units with different colors represent different SRS and repeat units, respectively.

**
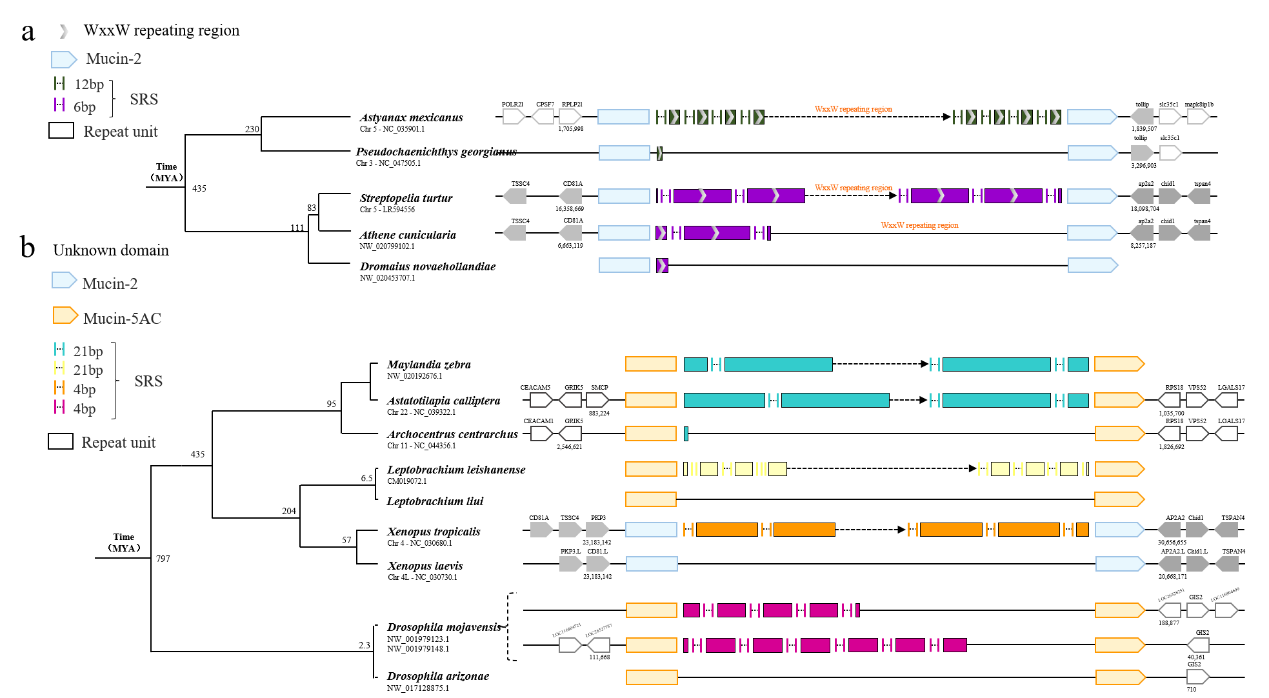
**

**Supplementary Method** for AFPIII positive BAC mapping and AFPIII locus sequencing

After 48 AFPIII positive BACs were isolated, the SNaPshot Multiplex kit (Applied Biosystems, USA) was used to fingerprint the 26 positive clones (26 out of 48 AFPIII positive BACs). The labeled restriction fragments were separated on a 3730 squencer, and information collected using GeneMapper v3.5. The data was cleaned of vector sequence using Genoprofiler and assembled with the FPC v8 software(Luo, Thomas et al. 2003) and the result was draw in Supplementary Figure. S4b. At the same time, we did fragment analysis after restriction enzyme cutting (EcoRI and HindIII) all 48 AFPIII positive BACs and finally, 20 BACs (Supplementary Figure S4c.) can cover all the length of DNA fragments of 48 AFPIII positive BACs.

6 BACs (008,001,003,007,005,004) which can cover most length of DNA fragments of 48 AFPIII positive BACs were chosen to shear to 1.5-2 kb in length and cloned to a PUC18 vector to construct a Shotgun library for sequencing. Contigs of each BAC clone were assembled using the Phred/Phrap/Consed package(Gordon 2003). The results showed 008 overlap with 001 in the 5’ end of AFPIII locus and 005 overlap with 004 in the 3’ end of AFPIII locus but not for 003 and 007. Then we designed the PCR primer for the specific sequences (the junction sequence where retrotransopon gene insertion to the AFPIII gene) of the 3’ end of 001, the 5’end of 005 and both 5’/3’ end of 003, 007. Fragment analysis of PCR results using 20 BACs (Supplementary Figure S4c.) as the templates showed the 001 overlaps with 009, 009 overlaps with 003, 003 overlaps with 011, 011 overlaps with 007, 007 overlaps with 010, 010 overlaps with 005 (Supplementary Figure S4d.). The gap1, 2, 3 were estimated by the size and fragment analysis of BAC009, 010 and 011. In summary, the 9 BACs can cover all the AFPIII gene locus (Supplementary Figure. S4a).

**Supplementary Figure S4a.** Nine BACs can cover the whole locus by High-throughput fingerprinting technology. These sequenced BACs are in black, and the non-sequenced BACs are in white. There are still three gaps with an average length about 20-40 kb.

**Supplementary Figure S4b.** High-throughput fingerprinting results of the 26 positive clones (26 out of 48 AFPIII positive BACs)

**Supplementary Figure S4c.** 20 BACs can cover all the length of DNA fragments of 48 AFPIII positive BACs.


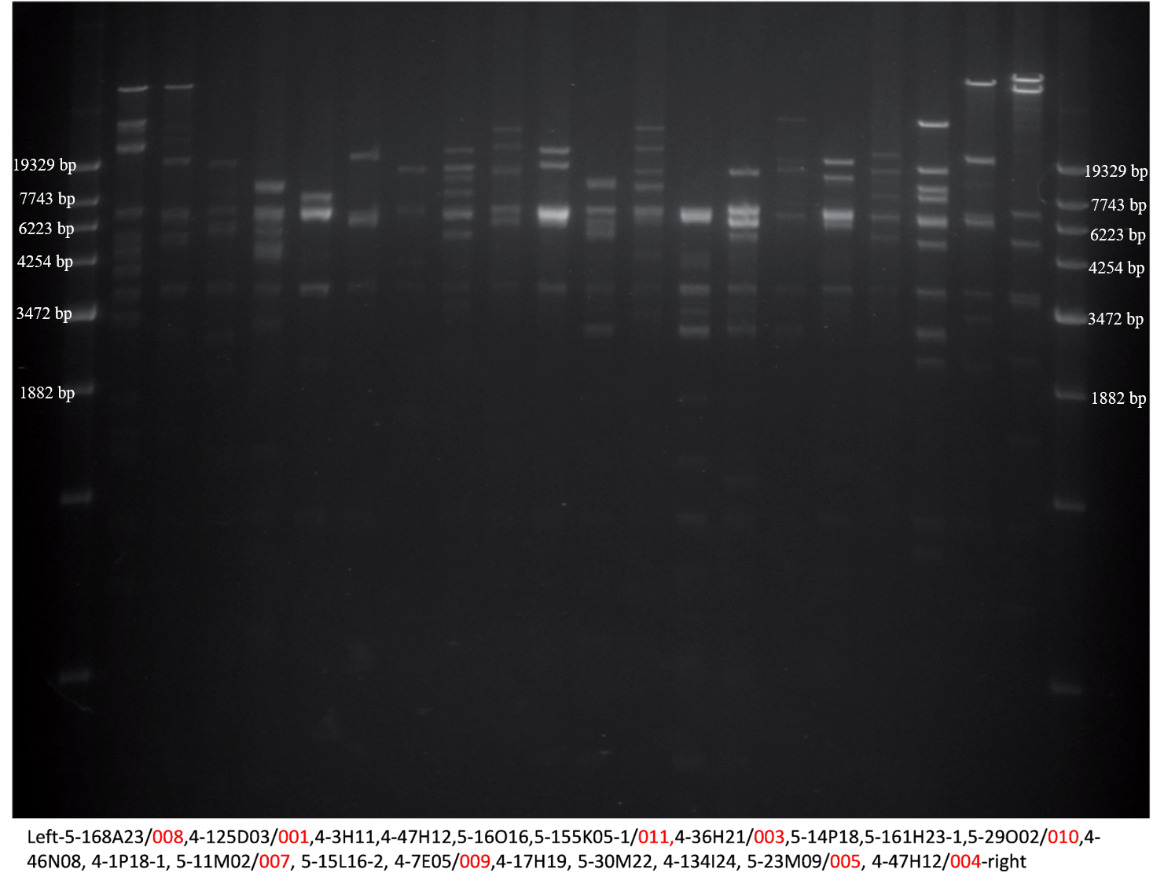


**Supplementary Figure S4d.** Fragment analysis of junction sequence PCR results using 20 BACs as the templates (from different gels).


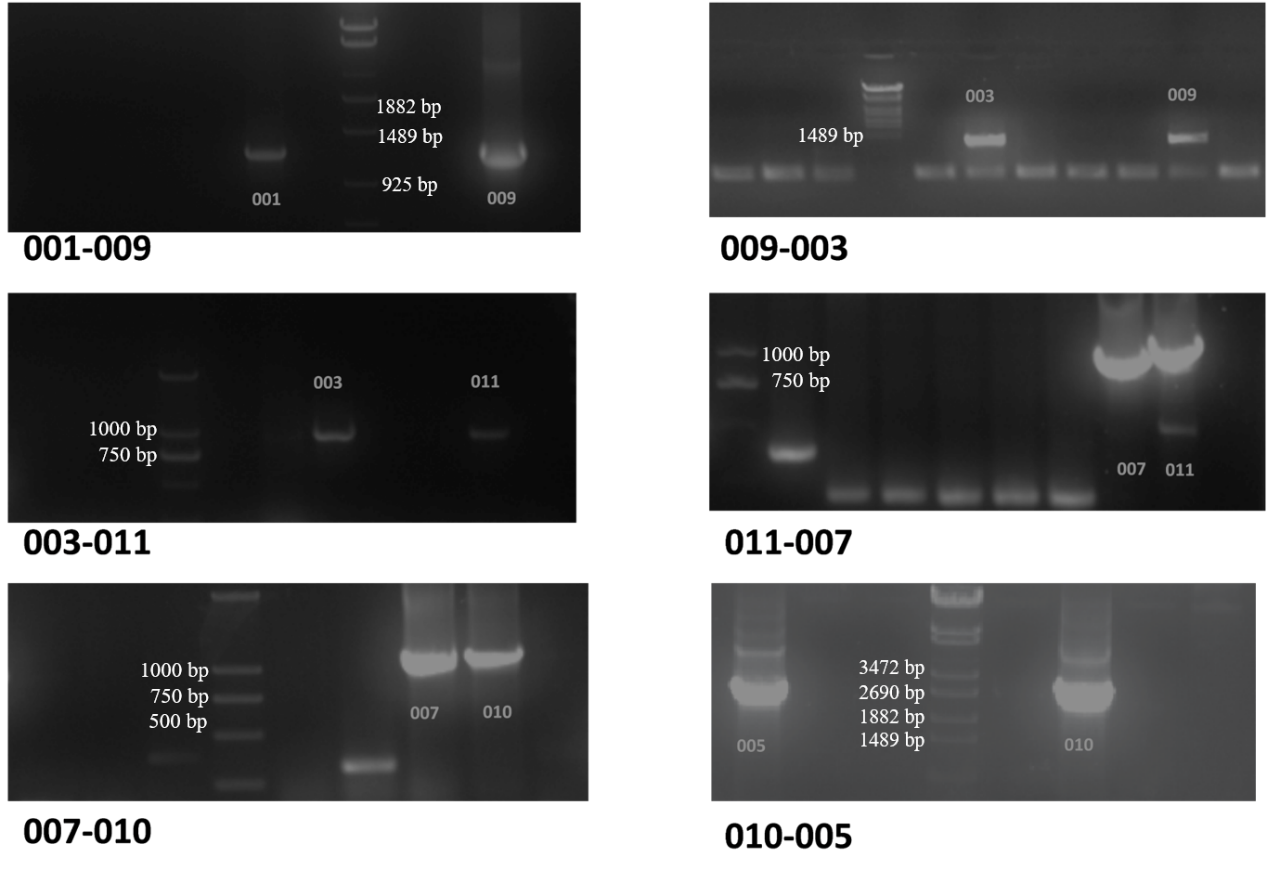


**Supplementary Figure S4e.** FISH localized the AFPIII gene family to a one position of one chromosome in *L. dearborni*.

**Supplementary Figure S5.** Nucleotide alignment of two 8kb units with AFPIII-7 and AFPIII-11.

AFPIII-7 ATGTGGTCCGAGCCATCAGACCTCAACGCGTCCTCAGTGGGATCTGATTTCATTCCCAAA

AFPIII-11 ATGTGGTCCGAGCCATCAGACCTCAACGCGTCCTCAGTGGGATCTGATTTCATTCCCAAA

************************************************************

AFPIII-7 CTGGAGTCGGACTAATGTTCCAGAACTCTCGTGCTGATAACTGATTGATGATGAAATCAT

AFPIII-11 CTGGAGTCGGACTAATGTTCCAGAACTCTCGTGCTGATAACTGATTGATGATGAAATCAT

************************************************************

AFPIII-7 GAACGTGAGGTCCTGATGTTGGCAGATGTGAAGAGTTTTGGAAAGAGAGGATGTTGCTCT

AFPIII-11 GAACGTGAGGTCCTGATGTTGGCAGATGTGAAGAGTTTTGGAAAGAGAGGATGTTGCTCT

************************************************************

AFPIII-7 GCGGGAACAGACCGGCCTGTGACTCCTGACGATTWTCAGATTWTTA-TWTCAGGA-GAAG

AFPIII-11 GCGGGAACAGACCGGCCTGTGACTCCTGACGATTATCAGATTATTAATATCAGGAAGAAG

********************************** ******* *** * ****** ****

AFPIII-7 AGATGTTCCAGAATGCAGGCGGTCCCGGGGGAGTTCCCCT-CTTTCTAACTGCAGCTRGG

AFPIII-11 AGATGTTCCAGAATGCAGGCGGTCCCGGGGGAGTTCCCCTTCTTTCTAACTGCAGCTCG-

**************************************** **************** *

AFPIII-7 TTCTGCTGACTACGGCCCGGCGTGTTGCTCTGAGCCGGTAATGAGGCAACGAAGATATAT

AFPIII-11 TTCTGCTGACTTACGGCCGGCGTGTTGCTCTGAGCCGGTAATGAGGCAACGAAGATATAT

*********** * ********************************************

AFPIII-7 GAAATATGATGCTCCGAGGATATGCTGAGGGAGGTTTTTGGTGTTTTTCATTACAGGATC

AFPIII-11 GAAATATGATGCTCCGAGGATATGCTGAGGGAGGTTTTTGGTGTTTTTCATTACAGGATC

************************************************************

AFPIII-7 AACATGTGACACAATCTGCACGTCACCATTAATCTGTCCATGTTCCTCGTTCTTATGACA

AFPIII-11 AACATGTGACACAATCTGCACGTCACCATTAATCTGTCCATGTTCCTCGTTCTTATGACA

************************************************************

AFPIII-7 GTGCACGGAGATACGATCACGCAGTTAACTGAAGCGCTGGCGGTGATGATCAGACTTCAA

AFPIII-11 GTGCACGGAGATACGATCACGCAGTTAACTGAAGCGCTGGCGGTGATGATCAGACTTCAA

************************************************************

AFPIII-7 TTAGCATTTTGTAGCAGCGGTGCATCTTAACTTAGGTTACCGGCGATCACTGGACCTCCC

AFPIII-11 TTAGCATTTTGTAGCAGCGGTGCATCTTAACTTAGGTTACCGGCGATCACTGGACCTCCC

************************************************************

AFPIII-7 GGCTGCTTTAGGGGAGAATAGGATGAGAGTCAGAGTTCTGCAGGAACTCATCAGACACGG

AFPIII-11 GGCTGCTTTAGGGGAGAATAGGATGAGAGTCAGAGTTCTGCAGGAACTCATCAGACACGG

************************************************************

AFPIII-7 GGATGAAATGTACAATCGCTCCTGCAGCACGACTTCACTCTCAGCCCATCGGCAGCATTA

AFPIII-11 GGATGAAATGTACAATCGCTCCTGCAGCACGACTTCACTCTCAGCCCATCGGCAGCATTA

************************************************************

AFPIII-7 AAGGTGACGTGTGAGGAACCGGCTCATCCTGTTCCAGGCTGCAGTTGATGTTTGCACTAC

AFPIII-11 AAGGTGACGTGTGAGGAACCGGCTCATCCTGTTCCAGGCTGCAGTTGATGTTTGCACTAC

************************************************************

AFPIII-7 ATGACTTGTTTGGTTTTCAGTCTCACTGTGACAGACGCACCTGATGACTGAGCGTAAATG

AFPIII-11 ATGACTTGTTTGGTTTTCAGTCTCACTGTGACAGACGCACCTGATGACTGAGCGTAAATG

************************************************************

AFPIII-7 AAGTTATTGAGATAAAGTCTGACGCTGACGTCGTCTTTCACATCCAGCTCGAGTCTCTCT

AFPIII-11 AAGTTATTGAGATAAAGTCTGACGCTGACGTCGTCTTTCACATCCAGCTCGAGTCTCTCT

************************************************************

AFPIII-7 GACCTCCAACACGGCGGCTGAGTCTGCTCATCATTATTCCCGTCCTCCGTGCTTCTTTCC

AFPIII-11 GACCTCCAACACGGCGGCTGAGTCTGCTCATCATTATTCCCGTCCTCCGTGCTTCTTTCC

************************************************************

AFPIII-7 CCGTTTTCCTGTGTGTGCGTGTGTACACATATAGACGCCGAGATCGAAAAGTGTTCGATG

AFPIII-11 CCGTTTTCCTGTGTGTGCGTGTGTACACATATAGACGCCGAGATCGAAAAGTGTTCGATG

************************************************************

AFPIII-7 GTGTGCCACTGTTGTTCAACAGTATGTACCCATCCCTGGGGTGGTATGCCAGTACCTTTC

AFPIII-11 GTGTGCCACTGTTGTTCAACAGTATGTACCCATCCCTGGGGTGGTATGCCAGTACCTTTC

************************************************************

AFPIII-7 CAACGGCACACCGTTTCCTGAAGGCAGGTGCTGCTTGAAAGTTCATATTAGTGGTATGTG

AFPIII-11 CAACGGCACACCGTTTCCTGAAGGCAGGTGCTGCTTGAAAGTTCATATTAGTGGTATGTG

************************************************************

AFPIII-7 CCGTTGAATCAGAGTGGTAACCTATAATGCTTGTTGTTTCCATCCTCTTCTCCCTTTTAT

AFPIII-11 CCGTTGAATCAGAGTGGTAACCTATAATGCTTGTTGTTTCCATCCTCTTCTCCCTTTTAT

************************************************************

AFPIII-7 CGCTGTCTSTCTTCTCTTCTTGCTGCTCTGTCGTTTCGGGCGGATGTT-GTCCAGGGTGC

AFPIII-11 CGCTGTCTCTCTTCTCTTCTTGCTGCTCTGTCGTTTCGGGCGGATGTTTGTCCAGGGTGC

******** *************************************** ***********

AFPIII-7 TGGATGAAGACTCCTACAAGCAGCCCTCCTCTGAGCTCATTGGTTATGTGTATGTTGCTC

AFPIII-11 TGGATGAAGACTCCTACAAGCAGCCCTCCTCTGAGCTCATTGGTTATGTGTATGTTGCTC

************************************************************

AFPIII-7 CAAATAACATCCTCCCTTCTCATGTTTTTAGATTTTTTCCCCCTCCGTTGTCATCAACTC

AFPIII-11 CAAATAACATCCTCCCTTCTCATGTTTTTAGATTTTTTCCCCCTCCGTTGTCATCAACTC

************************************************************

AFPIII-7 CGAGCATCCAACCATTGTCATCATCTCATTACTAGTTAGGGCAGTACAGTTACCTCCCCT

AFPIII-11 CGAGCATCCAACCATTGTCATCATCTCATTACTAGTTAGGGCAGTACAGTTACCTCCCCT

************************************************************

AFPIII-7 TGCATTTTTTAGTTCCATATTCATGTTTTAACTACTTTGATTTTGAATTGTTACATGCTT

AFPIII-11 TGCATTTTTTAGTTCCATATTCATGTTTTAACTACTTTGATTTTGAATTGTTACATGCTT

************************************************************

AFPIII-7 CACCTAGCTTTTTCTGTCTTTTTCAAATGTCAACCACAGTAATAATTATTCCCCCATATG

AFPIII-11 CACCTAGCTTTTTCTGTCTTTTTCAAATGTCAACCACAGTAATAATTATTCCCCCATATG

************************************************************

AFPIII-7 TCAAATACATGACACACATTTCCAACAATTAACACTTACAATAATTGTCTGAAGTGTTTT

AFPIII-11 TCAAATACATGACACACATTTCCAACAATTAACACTTACAATAATTGTCTGAAGTGTTTT

************************************************************

AFPIII-7 GTTTCTACTTAACACCTTTTTATTTCTTTCCCCAAATTAATCTTCAAAATATATTATTCA

AFPIII-11 GTTTCTACTTAACACCTTTTTATTTCTTTCCCCAAATTAATCTTCAAAATATATTATTCA

************************************************************

AFPIII-7 GGAGTAGGATGCGTATAGATTTGTTTAAAGCAAGTGTGTTTTAATAATTGAACTTGGGTG

AFPIII-11 GGAGTAGGATGCGTATAGATTTGTTTAAAGCAAGTGTGTTTTAATAATTGAACTTGGGTG

************************************************************

AFPIII-7 TTTTTGCCATTAATAATATGATAAATTGAACTTATAGCTGCACTAATCAACATTTTGTAA

AFPIII-11 TTTTTGCCATTAATAATATGATAAATTGAACTTATAGCTGCACTAATCAACATTTTGTAA

************************************************************

AFPIII-7 CAGTGCGTCACGATTGTGTAATATGAAAGAGGCCGGTGTGGTGGTGTGGTTTGATGCACC

AFPIII-11 CAGTGCGTCACGATTGTGTAATATGAAAGAGGCCGGTGTGGTGGTGTGGTTTGATGCACC

************************************************************

AFPIII-7 AGATGCAGACAACCGTGCACAGTCAAATGTGGGATGTGACGACATGTGCAGTCTTTCAGG

AFPIII-11 AGATGCAGACAACCGTGCACAGTCAAATGTGGGATGTGACGACATGTGCAGTCTTTCAGG

************************************************************

AFPIII-7 TCAGATCACGGCAAATATAAGCACTTCCTGTTCTCTTTGAGATATGCACTATGGCAGGCT

AFPIII-11 TCAGATCACGGCAAATATAAGCACTTCCTGTTCTCTTTGAGATATGCACTATGGCAGGCT

************************************************************

AFPIII-7 TGTAAAATGTCCTCACTCAATGGCAACATCTCTCATATGTGATATGAACATCACCATCTC

AFPIII-11 TGTAAAATGTCCTCACTCAATGGCAACATCTCTCATATGTGATATGAACATCACCATCTC

************************************************************

AFPIII-7 TGTTTGAAAACAAATTCACCCCTGACAACTTTTCCTA-----TATTAAAATTGCGTTTGT

AFPIII-11 TGTTTGAAAACAAATTCACCCCTGACAACTTTTCCTAGCACTTATTAAAATTGCGTTTGT

************************************* ******************

AFPIII-7 AGTCTCTACCGAGATACATGTAGGCCTGCTGTATTTGAGGAAGGTTACAGTTTTTCGAGG

AFPIII-11 AGTCTCTACCGAGATACATGTAGGCCTGCTGTATTTGAGGAAGGTTACAGTTTTTCGAGG

************************************************************

AFPIII-7 GTGCTACGTGTGGCCCTTTAATAGTCATAGTTTCTATGCAATTGAAGGCTATATGCAAAC

AFPIII-11 GTGCTACGTGTGGCCCTTTAATAGTCATAGTTTCTATGCAATTGAAGGCTATATGCAAAC

************************************************************

AFPIII-7 AGGTGCACAGTCTGTTTATAAGCATCATGGAAAAGTACAAGCATTTTGCACACACTCTGT

AFPIII-11 AGGTGCACAGTCTGTTTATAAGCATCATGGAAAAGTACAAGCATTTTGCACACACTCTGT

************************************************************

AFPIII-7 ATTTTTCCAATCGCTAACAATGTCATCGTGACATTGTGCTATTGGAAAGAGACCAGCTGA

AFPIII-11 ATTTTTCCAATCGCTAACAATGTCATCGTGACATTGTGCTATTGGAAAGAGACCAGCTGA

************************************************************

AFPIII-7 TCTAGACAGTTGATATCATGATCAACAGCCCCAAACAAGTGCGCATGCGTGAGGAGTGAT

AFPIII-11 TCTAGACAGTTGATATCATGATCAACAGCCCCAAACAAGTGCGCATGCGTGAGGAGTGAT

************************************************************

AFPIII-7 TGGCAGATGTATGAGAACTGAACCACGGGCTTAGGCATGCAAGAGTAGTCTTCTTGACTG

AFPIII-11 TGGCAGATGTATGAGAACTGAACCACGGGCTTAGGCATGCAAGAGTAGTCTTCTTGACTG

************************************************************

AFPIII-7 AACTTGCGCTAGAGCTTCATTGGAGGCATCTATTTTGTCTTTTCTCGTATGATTTTGGGA

AFPIII-11 AACTTGCGCTAGAGCTTCATTGGAGGCATCTATTTTGTCTTTTCTCGTATGATTTTGGGA

************************************************************

AFPIII-7 TGGACGCATGGAAGAGTTTTTCCCGTCTCAGCTTGCTTTTTACCCTAAATATTGTATACC

AFPIII-11 TGGACGCATGGGAGAGTTTTTCCCGTCTCAGCTTGCTTTTTACCCTAAATATTGTATACC

*********** ************************************************

AFPIII-7 TATTAGAACCGTTGTCACAGGGTTCAAATTAACATTTTCGTTTAGTTTTGATCATGATAT

AFPIII-11 TATTAGAACCGTTGTCACAGGGTTCAAATTAACATTTTCGTTTAGTTTTGATCATGATAT

************************************************************

AFPIII-7 ATACGTTTTATCCATAAGGCATAGAACATGTGCATATACAGTAAGGGCTTGTTATACGAC

AFPIII-11 ATACGTTTTATCCATAAGGCATAGAACATGTGCATATACAGTAAGGGCTTGTTATACGAC

************************************************************

AFPIII-7 AGAAAGAAGAAGAGGATATGCGTGCAGGCTGTCAGCTAATAAATGGATCACAAGTTATAG

AFPIII-11 AGAAAGAAGAAGAGGATATGCGTGCAGGCTGTCAGCTAATAAATGGATCACAAGTTATAG

************************************************************

AFPIII-7 AATGCAAGCTGGTGATAGTTTGCACAAAAACAAGTTATACTTTACTTATAAGAATATAAA

AFPIII-11 AATGCAAGCTGGTGATAGTTTGCACAAAAACAAGTTATACTTTACTTATAAGAATATAAA

************************************************************

AFPIII-7 ATTTCCATTACAATTGGCATAAGGAGGTGTGATACAGTGACCTACTTTCAGGCCAATAGG

AFPIII-11 ATTTCCATTACAATTGGCATAAGGAGGTGTGATACAGTGACCTACTTTCAGGCCAATAGG

************************************************************

AFPIII-7 AAACGGGATATGCCGGTTAAGTCCTCCCACATACTGTATATTAGATGCAGCACATGCACC

AFPIII-11 AAACGGGATATGCCGGTTAAGTCCTCCCACATACTGTATATTAGATGCAGCACATGCACC

************************************************************

AFPIII-7 TGTCCTGTCAGAAGTCTCAGCTACAGCTTTCACTTCGTTCTCCGCTAATTAATTAATTAC

AFPIII-11 TGTCCTGTCAGAAGTCTCAGCTACAGCTTTCACTTCGTTCTCCGCTAATTAATTAATTAC

************************************************************

AFPIII-7 TAATTAATTAAGTCTCAGCCACAGCCATGAAGTCAGTTGTTTTAACTGGTTTGCTGTTCG

AFPIII-11 TAATTAATTAAGTCTCAGCCACAGCCATGAAGTCAGTTGTTTTAACTGGTTTGCTGTTCG

************************************************************

AFPIII-7 TCCTCCTTTGTGTCGACCACATGAGTTCAGCCAACAAGGTGAGATATTCTTGCTCCACAA

AFPIII-11 TCCTCCTTTGTGTCGACCACATGAGTTCAGCCAACAAGGTGAGATATTCTTGCTCCACAA

************************************************************

AFPIII-7 AAAAATATTCAAAAATGTTAGCCACAGTAAAATTCAATTGTTTTCTGTTCAGAAAGACAG

AFPIII-11 AAAAATATTCAAAAATGTTAGCCACAGTAAAATTCAATTGTTTTCTGTTCAGAAAGACAG

************************************************************

AFPIII-7 AGAACCTTTAAGAAAACATTTTTGAAATAGTTTTTTCAACTGTGCCATGAGAACATTAAT

AFPIII-11 AGAACCTTTAAGAAAACATTTTTGAAATAGTTTTTTCAACTGTGCCATGAGAACATTAAT

************************************************************

AFPIII-7 AATGTCTGACCTTTTATTTTCCATTCTTCAAGGCGTCCGTGGTGGCCAACCAGCTGATCC

AFPIII-11 AATGTCTGACCTTTTATTTTCCATTCTTCAAGGCGTCCGTGGTGGCCAACCAGCTGATCC

************************************************************

AFPIII-7 CCATAAATACTGCCCTGACTCTGATAATGATGAAGGCGGAGGTGGTCACCCCAATGGGCA

AFPIII-11 CCATAAATACTGCCCTGACTCTGATAATGATGAAGGCGGAGGTGGTCACCCCAATGGGCA

************************************************************

AFPIII-7 TCCCCGCCGAGGACATTCCCCGAATAATCGGAATGCAAGTGAACAGGGCAGTGCCGTTGG

AFPIII-11 TCCCCGCCGAGGACATTCCCCGAATAATCGGAATGCAAGTGAACAGGGCAGTGCCGTTGG

************************************************************

AFPIII-7 GCACAACCCTCATGCCAGACATGGTGAAAAACTATGAGAAGTAGTTCTGAGCGTGCCAAG

AFPIII-11 GCACAACCCTCATGCCAGACATGGTGAAAAACTATGAGAAGTAGTTCTGAGCGTGCCAAG

************************************************************

AFPIII-7 GAGTTTCTTCCCAAAACCAAAAGAAGAAATGCCTCCTCTCACAATTAACCTTGTTTTTGT

AFPIII-11 GAGTTTCTTCCCAAAACCAAAAGAAGAAATGCCTCCTCTCACAATTAACCTTGTTTTTGT

************************************************************

AFPIII-7 CACAAACCCAAGTCTGTCCGTATGTCAACTGAACATGTCAAAACCTGTGGAGACTGTTGA

AFPIII-11 CACAAACCCAAGTCTGTCCGTATGTCAACTGAACATGTCAAAACCTGTGGAGACTGTTGA

************************************************************

AFPIII-7 GATTTGATGTTCTGAAAAGATAAAGCCTATATAAAATGTTGCCCAAATTTCCTGCCTGAG

AFPIII-11 GATTTGATGTTCTGAAAAGATAAAGCCTAT--AAAATGTTGCCCAAATTTCCTGCCTGAG

****************************** ****************************

AFPIII-7 GTTTTTCCTTGTCTTTGATACATCGCTTTGCTGCTCGGATCGACTCACTCTGTGTATGCC

AFPIII-11 GTTTTTCCTTGTCTTTGATACATCGCTTTGCTGCTCGGATCGACTCACTCTGTGTATGCC

************************************************************

AFPIII-7 ACATTCACTTTGTACTCTCCTTCTCACGGTAGGTTTATTATTTTTAGATGTGCAGTTAGT

AFPIII-11 ACATTCACTTTGTACTCTCCTTCTCACGGTAGGTTTATTATTTTTAGATGTGCAGTTAGT

************************************************************

AFPIII-7 TTCTGTGAAATAACATACCACACACTGATATTGTCTGTGCATTGACTTGGTGAGTGCACA

AFPIII-11 TTCTGTGAAATAACATACCACACACTGATATTGTCTGTGCATTGACTTGGTGAGTGCACA

************************************************************

AFPIII-7 TTGTTTTTGATCTGGACATATTTATATTTGATTGATCAGGTGATGTGTGAATCTAAAGTG

AFPIII-11 TTGTTTTTGATCTGGACATATTTATATTTGATTGATCAGGTGATGTGTGAATCTAAAGTG

************************************************************

AFPIII-7 CTCCATACAGATGTTCTGCATTGAAAATATTCAATTGTGAGTAAATAGACGGCTTCATTC

AFPIII-11 CTCCATACAGATGTTCTGCATTGAAAATATTCAATTGTGAGTAAATAGACGGCTTCATTC

************************************************************

AFPIII-7 CAGTGGCGGCTGGTGGAAAATGTTCTAGGTAGGGCTGTGCAACATCCAATCAATTTCAGC

AFPIII-11 CAGTGGCGGCTGGTGGAAAATGTTCTAGGTAGGGCTGTGCAACATCCAATCAATTTCAGC

************************************************************

AFPIII-7 AAACATCCCAGTATGATTTAATGCAAAGAACTAAGGTATCAAACATGCATCACTACTTTG

AFPIII-11 AAACATCCCAGTATGATTTAATGCAAAGAACTAAGGTATCAAACATGCATCACTACTTTG

************************************************************

AFPIII-7 CAGTTAAATATTTAACATTTAAACTAACACGAAAAAAAGCAGTAAATAACACTTTGACAA

AFPIII-11 CAGTTAAATATTTAACATTTAAACTAACACGAAAAAAAGCAGTAAATAACACTTTGACAA

************************************************************

AFPIII-7 ACACGTCAGGACATCTTATTTTTGTCACCCTCACAGGCAATTCAGGATACTATGATAATA

AFPIII-11 ACACGTCAGGACATCTTATTTTTGTCACCCTCACAGGCAATTCAGGATACTATGATAATA

************************************************************

AFPIII-7 TCTATATATCATATTATAATATTCAGTATAATATAAAACACGCACGTATATATATATATA

AFPIII-11 TCTATATATCATATTATAATATTCAGTATAATATAAAACACGCACGTATATATATATATA

************************************************************

AFPIII-7 TATATATATATATATTTAATTTAATATAATATAATAGAACATGTTTATTTATTGAGATGC

AFPIII-11 TATATATATATA--TTTAATTTAATATAATATAATAGAACATGTTTATTTATTGAGATGC

************ **********************************************

AFPIII-7 CCCTATGGACCTTTATAGGAGGTAAAGATCAGGAGAAGTAAACATGAAGTGTAATTATGA

AFPIII-11 CCCTATGGACCTTTATAGGAGGTAAAGATCAGGAGAAGTAAACATGAAGTGTAATTATGA

************************************************************

AFPIII-7 ATACTGGTGTTAAATTAAGCCATGATGAGTTTTCACTGTTAATTTACCATCTCTATTAAA

AFPIII-11 ATACTGGTGTTAAATTAAGCCATGATGAGTTTTCACTGTTAATTTACCATCTCTATTAAA

************************************************************

AFPIII-7 TGTTGATGCCTCCATGACCAAGTTAAGCAGATGAGACTCTAGAAGAAAAGATGTTCACTT

AFPIII-11 TGTTGATGCCTCCATGACCAAGTTAAGCAGATGAGACTCTAGAAGAAAAGATGTTCACTT

************************************************************

AFPIII-7 TGCTGAATATGGCTAGCTTGACAGAGTTATCCATGGATATATAAATATATATATATGTAT

AFPIII-11 TGCTGAATATGGCTAGCTTGACAGAGTTATCCATGGATATATAAATATATATATATGTAT

************************************************************

AFPIII-7 ATATATATTTATAAAATGATTTATTTATAACTATATATCCATTTCTCAGACAGGTGCTTC

AFPIII-11 ATATATATTTATAAAATGATTTATTTATAACTATATATCCATTTCTCAGACAGGTGCTTC

************************************************************

AFPIII-7 ATGTCCCTCACTTCCGTAACTGTCCATGCTGGATCTGTCCCCGTTGTTTTAAAAAGCTGG

AFPIII-11 ATGTCCCTCACTTCCGTAACTGTCCATGCTGGATCTGTCCCCGTTGTTTTAAAAAGCTGG

************************************************************

AFPIII-7 GATCAGCTCTTTCACTGTAGTTTCTCCGCCAAAGTTCTCCTCTCAAATGGGTCTTTTGAG

AFPIII-11 GATCAGCTCTTTCACTGTAGTTTCTCCGCCAAAGTTCTCCTCTCAAATGGGTCTTTTGAG

************************************************************

AFPIII-7 CTCGCGCTGCCTGTAGAGACTCGTTCTGACGAGCATGCGCAGTTTGGGGCAAAACAGATT

AFPIII-11 CTCGCGCTGCCTGTAGAGACTCGTTCTGACGAGCATGCGCAGTTTGGGGCAAAACAGATT

************************************************************

AFPIII-7 GTGCTCATTGGTTAAATCTATTTGAGGACACTGATTGGCTAAATTGTATGTCACACTTTT

AFPIII-11 GTGCTCATTGGTTAAATCTATTTGAGGACACTGATTGGCTAAATTGTATGTCACACTTTT

************************************************************

AFPIII-7 ATATCTTTATTCAGCAATTGGCTAAACTGCTTCATAGACCCGCCTCCTGTGAGAACTTCC

AFPIII-11 ATATCTTTATTCAGCAATTGGCTAAACTGCTTCATAGACCCGCCTCCTGTGAGAACTTCC

************************************************************

AFPIII-7 TCCAGAGCCACAGCGCTGCTGAGACATGCAGACAGGCAGAGAGGACGTGCAGGAAAAGCA

AFPIII-11 TCCAGAGCCACAGCGCTGCTGAGACATGCAGACAGGCAGAGAGGACGTGCAGGAAAAGCA

************************************************************

AFPIII-7 GATATTATGAGCAGATATCATATTTTACACATATTACTGGATGCATTCCATATTTCAAAA

AFPIII-11 GATATTATGAGCAGATATCATATTTTACACATATTACTGGATGCATTCCATATTTCAAAA

************************************************************

AFPIII-7 ACACAAATATTGACAATTTGTATCGTTTTTTATTATTATTACTTTTTGTGGCTCATGGGG

AFPIII-11 ACACAAATATTGACAATTTGTATCGTTTTTTATTATTATTACTTTTTGTGGCTCATGGGG

************************************************************

AFPIII-7 GGGGGCGGCGCCCTAGTGCCCTCTATTGACAAGCTGCATGCTTATGGTTCATCAGACACC

AFPIII-11 GGGG-CGGCGCCCTAGTGCCCTCTATTGACAAGCTGCATGCTTATGGTTCATCAGACACC

**** *******************************************************

AFPIII-7 AATCCAAAACGTTCAGTATTTTATTGCCATATCAATCACAGTGCCTTCTGAATTTGTTTT

AFPIII-11 AATCCAAAACGTTCAGTATTTTATTGCCATATCAATCACAGTGCCTTCTGAATTTGTTTT

************************************************************

AFPIII-7 GTTGCGGTCAACCACAAAAACGTATAATCACAGAGCACACAGAACAAAAACACACTGCTA

AFPIII-11 GTTGCGGTCAACCACAAAAACGTATAATCACAGAGCACACAGAACAAAAACACACTGCTA

************************************************************

AFPIII-7 CAAGTACAATACAGTAAGCATACAATAGTGCAGCCAAATGGCTCACGCCCTTACAGTGAC

AFPIII-11 CAAGTACAATACAGTAAGCATACAATAGTGCAGCCAAATGGCTCACGCCCTTACAGTGAC

************************************************************

AFPIII-7 AAGTGGAAGTGCATGTGCTTGATTGCACAACCAGTGAAGCGAGGTATTGTAGTCTAAATA

AFPIII-11 AAGTGGAAGTGCATGTGCTTGATTGCACAACCAGTGAAGCGAGGTATTCTAGTCTAAATA

************************************************ ***********

AFPIII-7 CTGCGTAGTCTTTTACCCGCAGGTTGAAGGCTGCATAGAGGGTTTGCTGGGTGTGAGGAG

AFPIII-11 CTGCGTAGTCTTTTACCCGCAGGTTGAAGGCTGCATAGAGGGTTTGCTGGGTGTGAGGAG

************************************************************

AFPIII-7 TCCTTTTGAATTCACAAACCTCTACGCTGAATGCAGACATGGTATAGTTCAGAACTAGGG

AFPIII-11 TCCTTTTGAATTCACAAACCTCTACGCTGAATGCAGACATGGTATAGTTCAGAACTAGGG

************************************************************

AFPIII-7 GGAGCGTACACAGCCAACTGTTTTTAAAGTTCTGTCCATGAGTTTTTTTCTCGTAGGCTG

AFPIII-11 G-AGCGTACACAGCCAACTGTTTTTAAAGTTCTGTCCATGAGTTTTTTTCTCGTAGGCTG

* **********************************************************

AFPIII-7 TAGAGTTTGCCATTGCACACAGTTATTGAGAAGTGAGCACTTTCTATTACCGCTCTGTAG

AFPIII-11 TAGAGTT-GCCATTGCACACAGTTATTGAGAAGTGAGCACTTTCTATTACCGCTCTGTAG

******* ****************************************************

AFPIII-7 AACTTTACCATCGCAACCTTTTACACATCAAATGTCCTAAGCTGTTGTTTATCGCCCCTC

AFPIII-11 AACTTTACCATCGCAACCTTTTACACATCAAATGTCCTAAGCTGTTGTTTATCGCCCCTC

************************************************************

AFPIII-7 TTGAATGCATTATTTGTTCTTCCTGCATAGCATCTTAATTAACATGCAACTATTTACTAA

AFPIII-11 TTGAATGCATTATTTGTTCTTCCTGCATAGCATCTTAATTAACATGCAACTATTTACTAA

************************************************************

AFPIII-7 ATCAAATCTCAGTTTAACAAGGGGAAGATAGATAGATAGATATACACTTTATTCATCCCC

AFPIII-11 ATCAAATCTCAGTTTAACAAGGGGAAGATAGATAGATAGATATACACTTTATTCATCCCC

************************************************************

AFPIII-7 AAGAAAAAAATTAGTTTGTAGCAGCAGCAATTTACAGAGTAAATAAAATCTGCAAGATGC

AFPIII-11 AAGAAAAAAATTAGTTTGTAGCAGCAGCAATTTACAGAGTAAATAAAATCTGCAAGATGC

************************************************************

AFPIII-7 TTTCTTAACAACACTTGCCACAGTTCAATTTAGAAGCTTATTTGGTGAGGACAGTACGAA

AFPIII-11 TTTCTTAACAACACTTGCCACAGTTCAATTTAGAAGCTTATTTGGTGAGGACAGTACGAA

************************************************************

AFPIII-7 AAGGTCTTTAGACATTTGGTCAGTTAATACTTTCTGCCAACACGTTTATATTTTGACACG

AFPIII-11 AAGGTCTTTAGACATTTGGTCAGTTAATACTTTCTGCCAACACGTTTATATTTTGACACG

************************************************************

AFPIII-7 TTAAGATTTCTGGTATTTCTTAGGGAAAACACAAATAGAAGTAATGTTGTACTTGAACCA

AFPIII-11 TTAAGATTTCTGGTATTTCTTAGGGAAAACACAAATAGAAGTAATGTTGTACTTGAACCA

************************************************************

AFPIII-7 CTAGATAGAGCTGTTGTACAGCTGGATTTATCCAAGCGAGCGTTTCAACTCATCTCTGAC

AFPIII-11 CTAGATAGAGCTGTTGTACAGCTGGATTTATCCAAGCGAGCGTTTCAACTCATCTCTGAC

************************************************************

AFPIII-7 TGAATACAGATAGGAACGTTGTTTTACCTCTCCTCAAAGGTTTCCATGGAGCATGGTTTC

AFPIII-11 TGAATACAGATAGGAACGTTGTTTTACCTCTCCTCAAAGGTTTCCATGGAGCATGGTTTC

************************************************************

AFPIII-7 ATCTGGACCCTGGTCCTTCCTCCTACCATGGCCCTGCTGACTCCAACTGCTAATATCACT

AFPIII-11 ATCTGGACCCTGGTCCTTCCTCCTACCATGGCCCTGCTGACTCCAACTGCTAATATCACT

************************************************************

AFPIII-7 TTTATTATTACTCACATTACTATTTCATACTACCGATACCATTAATGCACATTATTGACC

AFPIII-11 TTTATTATTACTCACATTACTATTTCATACTACCGATACCATTAATGCACATTATTGACC

************************************************************

AFPIII-7 TGCTTCTTCACCAGAGTCTCTGTGCTTTCTCGCCTCACAGGTTCCTGTGTATCCGGGTTT

AFPIII-11 TGCTTCTTCACCAGAGTCTCTGTGCTTTCTCGCCTCACAGGTTCCTGTGTATCCGGGTTT

************************************************************

AFPIII-7 ACCTGGACCAGGTGTTGCCTCCTGTCGTGGTAGTGTCATACATTGAATGTGCTGTAATTA

AFPIII-11 ACCTGGACCAGGTGTTGCCTCCTGTCGTGGTAGTGTCATACATTGAATGTGCTGTAATTA

************************************************************

AFPIII-7 CATTGAATGTGTTGTAATTCACTATCCTGTTCTACACATGACATCATTGCACTTCTGTCC

AFPIII-11 CATTGAATGTGTTGTAATTCACTATCCTGTTCTACACATGACATCATTGCACTTCTGTCC

************************************************************

AFPIII-7 ATCCTGGGAGAGGGATCCCTCCTCTATTGCTCTCCCACAGGTTTCTTCCTCTTTCCTCCC

AFPIII-11 ATCCTGGGAGAGGGATCCCTCCTCTATTGCTCTCCCACAGGTTTCTTCCTCTTTCCTCCC

************************************************************

AFPIII-7 CGTTAAAGGGGTTTTTTGGGGAGTTTTTCCTGTTCTGATGTGAGGGTTCCGGGACAAGAG

AFPIII-11 CGTTAAAGGGGTTTTTTGGGGAGTTTTTCCTGTTCTGATGTGAGGGTTCCGGGACAAGAG

************************************************************

AFPIII-7 GATGTCAAATGTGTACATATTGTAAAGCCCTCTGAGGCAAATTTGTAATTTGTGATTTTG

AFPIII-11 GATGTCAAATGTGTACATATTGTAAAGCCCTCTGAGGCAAATTTGTAATTTGTGATTTTG

************************************************************

AFPIII-7 GGCTATACAAAAATAAATTGAATTGAATTGAATTGAATTGTTTAACTCCAGCCAGCCAGT

AFPIII-11 GGCTATACAAAAATAAATTGAATTGAATTGAATTGAATTGTTTAACTCCAGCCAGCCAGT

************************************************************

AFPIII-7 ATTGTAATGACCTGTAAACTGTTTGTTGTGTACATTAATGTCCCAAATGTCCAAAAACGC

AFPIII-11 ATTGTAATGACCTGTAAACTGTTTGTTGTGTACATTAATGTCCCAAATGTCCAAAAACGC

************************************************************

AFPIII-7 TCTCCCTCGTGGAATTAGTTTTGTATGTGAAGAGCTGATGTGTTACGGAACACCACAGAT

AFPIII-11 TCTCCCTTGTGGAATTAGTTTTGTATGTGAAGAGCTGATGTGTTACGGAACACCACAGAT

******* ****************************************************

AFPIII-7 CTGTAACGAGGACTCAGAGCGACGTGTCGGTGAGGCTGCCTGGAGACTTCACGTCTTCTG

AFPIII-11 CTGTAACGAGGACTCAGAGCGACGTGTCGGTGAGGCTGCCTGGAGACTTCACGTCTTCTG

************************************************************

AFPIII-7 GACGCCTTCGACCGAACGCTAACGCGTCTGTTCACGGCTCCACAGACGACACGATGCTGA

AFPIII-11 GACGCCTTCGACCGAACGCTAACGCGTCTGTTCACGGCTCCACGGACGACACGATGCTGA

******************************************* ****************

AFPIII-7 AGATCAAACGCAGTAACGACGTTACCGGCTGACTCCTGCTTCCCTTCGTGTCCTATGAGT

AFPIII-11 AGATCAAACGCAGTAACGACGTTACCGGCTGACTCCTGCTTCCCTTCGTGTCCTATGAGT

************************************************************

AFPIII-7 GCAATATCTGGGCCTTTGGAGGTTTTTACATTTTTAACGGCATAATAAAAACTAATGTAT

AFPIII-11 GCAATATCTGGGCCTTTGGAGGTTTTTACATTTTTAACGGCACAATAAAAACTAATGTAT

****************************************** *****************

AFPIII-7 TCCTTTAGAGCGTCGTGTAGCTTGGCTGCATTTTAATAAAACAACATCTTCAAGTGATGT

AFPIII-11 TCCTTTAGAGCGTCGTGTAGCTTGGCTGCATTTTAATAAAACAACATCTTCAAGTGATGT

************************************************************

AFPIII-7 GTWRSWGTTGATCGGGTCATTGTCTGGAGGGCTGGTGCATGTTTGGGTTTGTCTGTCATG

AFPIII-11 GTTCCTGTTGTTCGGGTCATTGTCTGGAGGGCTGGTGCATGTTTGGGTTTGTCTGTCATG

** **** *************************************************

AFPIII-7 TTAGACACTCAAACACGTCTTTAAACCTTTTATAATGTTTAAATGTCTTTTAAACTGGTA

AFPIII-11 TTAAACACTCAAACACGTCTTTAAACCTTTTATAATGTTTAAATGTCTTTTAAACTGGTA

*** ********************************************************

AFPIII-7 ACGACACTAAAGAAGTTAAATCAAGCTGTTGCAGGAGGGAGACGGGAACGCAACACGAGT

AFPIII-11 ACGACACTAAAGAAGTTAAATCAAGCTGTTGCAGGAGGGAGACGGGAACGCAACACGAGT

************************************************************

AFPIII-7 TATGAAACCAAAGGGAGATGTTTCCCGCTGGTAAACCTCCGACTTCACAGGCAGTTACGT

AFPIII-11 TATGAAACCAAAGGGAGATGTTTCCCGCTGGTAAACCTCCGACTTCACAGGCAGTTACGT

************************************************************

AFPIII-7 CTCGTCAAAAAAACCACTCAGTGAGTTCAGGAGACGAATGAGTTCGACGTTTCACCGTGT

AFPIII-11 CTCGTCAAAAAAACCACTCAGTGAGTTCAGGAGACGAATGAGTTCGACGTTTCACCGTGT

************************************************************

AFPIII-7 TCACCACCAACACGCCGATTGTTGCATTAATCTCCAAAACACCAGAAGACGTACAAACTA

AFPIII-11 TCACCACCAACACGCCGATTGTTGCATTAATCTCCAAAACACCAGAAGACGTACAAACTA

************************************************************

AFPIII-7 AATGAACCGCGAAGAGTCAAACAGACCTAAATCAAACTCATCTCATATTCATGGACTCAT

AFPIII-11 AATGAACCGCGAAGAGTCAAACAGACCTAAATCAAACTCATCTCATATTCATGGACTCAT

************************************************************

AFPIII-7 TTATTACCTTTATATAAACCCTGTAAAGAAAGAAGTCAAGTACTAAATGAATTATACAGA

AFPIII-11 TTATTACCTTTATATAAACCCTGTAAAGAAAGAAGTCAAGTACTAAATGAATTATACAGA

************************************************************

AFPIII-7 AACAAAGGTTGAATATCTTCTGTTAAACTGTTTGATTTGGCTGATAAATATTTACTCAGA

AFPIII-11 AACAAAGGTTGAATATCTTCTGTTAAACTGTTTGATTTGGCTGATAAATATTTACTCAGA

************************************************************

AFPIII-7 TGGAGATTTTTAACGCTTCCTTCTCTGTGAAATCCTCAGACACGTGAACAGTGAACTATG

AFPIII-11 TGGAGATTTTTAACGCTTCCTTCTCTGTGAAATCCTCAGACACGTGAACAGTGAACTATG

************************************************************

AFPIII-7 AGACATTCACATCTGAAGAGATCCGAGCGCTGGTTAGAAACTCTAAACGTGCAGATCCAG

AFPIII-11 AGACATTCACATCTGAAGAGATCCGAGCGCTGGTTAGAAACTCTAAACGTGCAGATCCAG

************************************************************

AFPIII-7 CACCACGACCACCTGTGGAGCCTTCAAGCACGACCGACAGAAAATACAAGTCAGAGGAGC

AFPIII-11 CACCACGACCACCTGTGGAGCCTTCAAGCACGACCGACAGAAAATACAAGTCAGAGGAGC

************************************************************

AFPIII-7 GACGATGGTCGACACACAACCGCGGTGAGAGCCGGAGATATGTCGTCATGGAAACATTAA

AFPIII-11 GACGATGGTCGACACACAACCGCGGTGAGAGCCGGAGATATGTCGTCATGGAAACATTAA

************************************************************

AFPIII-7 AGTAAGTGCTGGGATGAAAGAACGTGGCCTC

AFPIII-11 AGTAAGTGCTGGGATGAAAGAACGTGGCCTC

*******************************

**Supplementary Figure S6.** The retrotransposable element Tf2 155 kDa protein type 1-like inserted 8 kb unit of the AFPIII-15.

>AFPIII-15

ATGTGGTCCGAGCCATCAGACCTCAACGCGTCCTCAGTGGGATCTGATTTCATTCCCAAACTGGAGTCGGACTAATGTTCCAGAACTCTCGTGCTGATAACTGATTGATGATGAAATCATGAACGTGAGGTCCTGATGTTGGCAGATGTGAAGAGTTTTGGAAAGAGAGGATGTTGCTCTGCGGGAACAGACCGGCCTGTGACTCCTGACGATTATCAGATTATTAATATCAGGAAGAAGAGATGTTCCAGAATGCAGGCGGTCCCGGGGGAGTTCCCCTTCTTTCTAACTGCAGCTCGTTCTGCTGACTTACGGCCGGCGTGTTGCTCTGAGCCGGTAATGAGGCAACGAAGATATATGAAATATGATGCTCCGAGGATATGCTGAGGGAGGTTTTTGGTGTTTTTCATTACAGGATCAACATGTGACACAATCTGCACGTCACCATTAATCTGTCCATGTTCCTCGTTCTTATGACAGTGCACGGAGATACGATCACGCAGTTAACTGAAGCGCTGGCGGTGATGATCAGACTTCAATTAGCATTTTGTAGCAGCGGTGCATCTTAACTTAGGTTACCGGCGATCACTGGACCTCCCGGCTGCTTTAGGGGAGAATAGGATGAGAGTCAGAGTTCTGCAGGAACTCATCAGACACG

GGCAGTGGCGGCTGGTGGAAAATGTTCTAGGTGGGGCCATCCTATCAATTTCAGCAAACATCCCAGTATGATTTAATGCAAAAAAAATGTAAGTATCAAACACGCATTATTACTTGGCAGTTAAATATTTAACATGTATTCCAACACGAAAACAATCAGTAAATAACACTTATATAATATAATATGACATGTTTATTTATTGAGATGCCCCTATGGACCTTGTTATAGGAGGTAAAGATCAGGAGAAGTAAACATGAAGTGTAATTATGAATACTGATGTTAAATTAAGCTATGATGAGTTTTCACTGTTAATTTAACATCTCTATTACATGTTGATGCCTCCATGACCAAGTTAAGCAGATGAGACTGAGGCTATGTAGAAGAAAAGATGTTCACTTTGCCGAATATGTAGCTTGACAGAGTTCTCCATGTGTGTCCTGGTGTTCTCATGTTTCCTTACCTCCTCACTCCCGTAACTGTCCGTGCTGTCCGTGTTGTTTTAAAAAGCTAAATAAATGATTAGCATGACGTCATCCAGCTAGCCAAACCTGTCTGGTGTACAGCTTGTTATGTGGTGGTGTAGTAGATGGACCCAAACGCACGCTCACACAGCAGATTAATCCTTCAAGATCTTTATTATCTCTATATATATATATATATATATATATATATAGCTGCAGACTTAAACAGACAACAATATCCAAACAGACTACGGCACCGTGTCCAGTGGCCAAAGCCTGTCCGTTCTCCCTGAGTCGGGAGGGAAACTCAACCGAGGAAGGATGATGCAGCCTCCAGAGTCGGGAGGGAAACTCAACCTTACTCACGGCTGGAAACGGTCCGGGATGAAAACAAGGAATGCCGACCGGTGAACTCCGTGGTACTACTCGGGTCGGCCTCTGCGCACAGGTGAATTCCCGGGGATCGACTCTGCGCGGCAGGAGTGCTGCAGACAGCCAGAAAAGGTTAGAAATAACTATTCATGAAAACACTGTTCAAACTCACGGAGACTAGAACCCGACTGGTAACCGTACGGACTGACAACGCGCCGGAGGACAGCTGGGGGTATTTAAGTCCGTCGGCGCTGATCACCGGAATTCCCCACAGCTGAGCCTGGGGAGAGGGGCGTGGTTGGAGATGGAACTCGGGGGTGGAAACTCTAGGCTGACACAAAAACAAAACATAAGCACATGGCCTTCAAACAACAACACTGACAGGGGGAAAACAGAGGAGCACACACTAGGTCAGACAGAGATCCTAACAGTACCCCCCCCTCAACGGGAGCCTCTTGGCGAACCGCCCGGCTTACCGGGGTGCTGTCGGTGGTAGGCGCATATGAGCCCCGGATCAACGATCCTGGACCGTGGCACCCAGCACCGGTCCTCAGGTCCATAACCCTCCCAGTCCACCAGGTACTGAAACCCCCGACCTCGACGGCGGACATCCAGGAGGCGATTGACCGTGTAGACGGGGCCCCCCTCGATGAGGCGGGGGGGTGGAGGGGGTGCAGGCGGAGGATTTAGGGGACTGGAGACAACAGGCTTGAGGAGGGAGACATGGAACACCGGGTGAATCCTCATGGAGCGAGGCAACAGCAGCTTGACCGACACCGGGTTGACGATGGAGATGATGGGAAAAGGACCGATGTATCTGGGGGAGAGCTTCCTCGACTCGGTGCGTAGGGGGATGTCTCTGGTCGAAAGCCAGACCCGGTGTCCAATACGGTATGCCGGAGCTGGGACACGACGCCGATCCGCCACTCTCCTATTCTGCTCAGCCGTGCGGAGCAGAGCCACCCGAGCCGCTCTCCACACCCGTCTACAACGACGGGCATTGTGTTGGACTGATGGAACTGCCACCTCGACCTCCTGGCTAGGGAACAGGGGAGGCTGATAGCCCAAGGAGGCCTCAAAAGGAGACAGGCCGGTGGCAGTAGACTTGTGGGAATTGTGGGCGTACTCAACCCACGGGAGGTGATGGCTCCAAGAGGAGGGATCAGTGGCGGTCACACACCTGAGGATGGCCTCGAGCTCCTGGTTCAGCCTCTCCGTCTGGCCGTTAGTTTGCGGATGGAATCCCGAGGAGAGACTGGCCGTGGCGCCGAGGGTGTGGCAGAACGCCTTCCATAACTGGGATGAGAACTGCGGGCCACGGTCCGAGACTATGTCCGCAGGAATGCCATGAAGCCTAAAGACGTGTTGGACCAGTAGGTCAGCGGTCTCACGGGCCGATGGAAGCTTGGGCAGGGGAATGAAGTGGGCCGCCTTTGAGAACCGGTCCACCACAGTCAGAATGACGGTGTTACCGTCTGACGGGGAGAGACCCGTTACGAAATCCACCGCAATGTGGGACCACGGACGTTGTGGAACAGGGAGTGGCCGTAGTAGCCCCATGGGGGGTCGGTGCGAGGCCTTGTTCCGGGCGCACGTAGTGCAGGCCCGCACATACGTACGGGTCTCCTCTTCGAGGGACGGCCACCAAAAGGTGCGGCGGAGCCAGTCTATCGTCCGCCGGATGCCGGGGTGACCAGAGAGCCGCGAGTCGTGGGCCCACCGCAGGACTTGGGAGCGGGCGGATGGAGGGACAAACAGACGATTCGGAGGGCCCCCTCCAGGGTCAGGTTGAGCCTGCTGGGCCTCACGTACCACCCTCTCGATCTCCCAGGTGACAGCACCGAGCACGTACGCCGCAGGCAGGATGGTGTCGGGAGTAGAGCTCCCCCCTTCCGGGGCAAACTGACGGGAGAGTGCATCCGGCTTGGTGTTCTTGTGACCCGGCCGATAGGTGATGGTGAAATTGAAGCGGGCGAAGAACAAAGCCCAACGGGCCTGGCGAGAATTGAGGCGCCTCGCCGTGTGGATATACTCCAGATTTCGGTGGTCGGTCCAAACGATGAAGGGGACCACCGATCCCTCCAGCCAGTGCCTCCACTCCTCCAAGGCGAACTTGACAGCCAACAGCTCACGGTTGCCCACGTCGTAGTTTCTCTCAGCAGGGGAAAGTCTACGGGAGAAGAAAGCACAAGGATGGAGTTTAGAGTCGGAAACTGCCCGCTGAGACAGTACGGCTCCGACTCCGGTGTCGGAGGCGTCCACCTCGACGACAAACTGCCGTGTCTCGTCTGGGTGGACCAGGATCGGAGCCGAGGCGAACCGCTCCTTGAGAGCAGAAAAAGCCCTGTCGGCCTCCGGGGTCCACGAAAACAACACCTTGGAGGAGGTGAGTCGCGTCAGCGGTTCTGCCGTTCGGCTGTAGTCCCGGATGAACCGTCTGTAGAAGTTCGCGAACCCCAAAAACTGCTGGAGCTTCTTTCGTGAGGTGGGCACCGGCCACTCCGTTACGGCTTTAACCTTCTCAGGGTCCTTTCTCACCTGACCCTCCTCAATGAAGACGCCCAGGAACTGCACAGAGGAGACGTGGAACTCGCACTTCTCAGCCTTAACGAAGAGCTGATTCTCCAACAGTCGTTGCAGCACCTGGCGGACATGGTTAGTGTGCTCCTGGGGATTCGGGGAGAAGATCAGAATGTCGTCAATATACACAAACACGAAACGGTTGAGGAAGTCGCGGAGGACATCATTAACCAGTGCCTGAAAGACGGCAGGGGCATTGGTCAATCCAAACGGCATCACCAGATATTCAAAGTGTCCCAAGGGTGTGTTAAAGGCCGTCTTCCACTCGTCTCCCTCGCGAATTCGGACCAGATGATACGCGTTGCGTAGGTCCAGCTTGGTGAATATCTTAGCTCCAGCTAAGGAGTCGAAAACAGACGAGATGAGTGGCAAAGGATATTTATTCTTGATAGTAATTTGATTCAAACCTCTGAAATCGATGCAGGGACGGAGGCTCTTGTCCTTCTTGGAGACGAAGAAGAAACCCGCTCCCACAGGCGAAGATGAGGGACGTATGATTCCTGCGGCGACCGACTCAGTTATATATTTCTCCATAGACTCGCGTTCTGGCCGGGAGAGATTATATAGCCGACTAGTGGGAAGTGGGGCGCCCGGGAGCAGGTCAATAGCACAATCGTAGGGGCGATGAGGGGGGAGTGAAAGAGCCCGCTCCTTGCTGAATGCCTCTCCTAAATCCAAATACTCCCGGGGCACGGCGGAGAGGCTCGGACCGGTGTCAGGTCTGCTGGGGGGAGGAGCTTGCCGGGGGACCGCAGATCTGAGGCAGCGAGAGTGACAGGTGGAGCTCCAGCCGGTGATCCTACCCCCAGCCCAATCGATCTGTGGATTATGATCCTTAAGCCAGGGAAGTCCCAGGACCAGGGGTGTCTGAGGGGTAGGAAACACGTAGAACCGGGTGTTCTGGCTGTGATTACCGGACACGAGCAGGGAAATTGGCTGAGTCTGGTGTGTTATCGCTGCCAGTGAACTGCCATCCAGTGCGTTCACTAGCTGAGCGCACTCCAACGCCTCCACGGGAATCCCATTACTCCTAACAAACTCGGTGTCCAAAAAGTTCTCTTCAGCCCCTGAATCAATGAGTGCCGACAGGTGCACAGGCCCAGAAGCTAAACTCAGGGTGGCAGGAAACAGTGCCCGAGGCTTAGAAGGGGGAATTCGCTCTGCATCGCTCACCAGCATCCCCACTCTTACTGGCGAGCGCTCTCTTTTGGTCGAACAGGACAGACGGCCCTGCGGTGTCCCGACTCACCACAGTACAGACAGCTTCCTGAGCGGATGCGTCGCTCTCTCTCCTCCGGAGAGAGATTAGTCCCCCCGAGCTGCATCAGCTCCTCCGGCTCGAAAGGGGGAGGGGCCGAGGTAGGAGAACGGGGGCGCGCAGAGCTGTACCGTCCGGACGGGCTCGGGAACTGAAGGCTGGGACGGAAACCCAACTGACGCACCGGATTGGACGGCCGGAAGTTCCTCTCCCGGCGGCGTTCCCGGATACGGTTATCCATCTTGATGGCCAGAGTGTAGAGCTCCTCTAATGACTCTGGCTCCTCCCGGACTGCGAGCTCATCCTTCAACACATCCGAAAGGCTGTGGCGATATATCCCCTTAAGCGCCTCCTTGTCCCAACCGGACTCCGTGGCCAGAATCTGGAAGTCCACCGTGTACTCCGCTGCGCTGATGGAACCCTGACGAAGACTGAGCAGGCGCTGAGCAACGTCCCTGGCGTAAACTGGGTGGTCAAATACCCTCCTGAACTCCTCCGCGAAGAGCGCGTAGGGAACCCGGTCTGGGCTGCGCCTGTTGAGCCAAGCCTCCGCCCAAACCAGCGCCTTGCCGCGCAGCAACCCCACCGTAAAGGCCACCCTCGCATTGTCCGAAGCAAATGTGCGGGGCTGCTGGTTAAAAACAAATCCACACTGGAGCATGAACCCTCTACACTCCCCGGACGCTCCATTATAGGGCACCGGTGAGGGAACAAAGGACTCACGGGTGCTGGATGCGTCCTCAGGGACCGGCTCCGAGGGAGGAGAATGGCGTAGCGTGAAGTCCTGCAGCAGGCCTCCTATCTCCGCGACCTGGGCCCCGATAGATCGCTGCGAGTCCGCTAGGTCCCGCAGAACCTGATGGTGTTGCCCGAGGAGCGCGCCGTGGCTGGCCATCGCCATCTGCAGCTGATCCGCAGAAAGAAACTCTGCTGCGGCGGCTGGGTCCGACATGGTCAGTCCGTTCTGTTATGTGGTGGTGTAGTAGATGGACCCAAACGCACGCTCACACAGCAGATTAATCCTTCAAGATCTTTATTATCTCTATATATATATATATATATATATAGCTGCAGACTTAAACAGACAACAATATCCAAACAGACTACGGCACCGTGTCCAGTGGCCAAAGCCTGTCCGTTCTCCCTGAGTCGGGAGGGAAACTCAACCGAGGAAGGATGATGCAGCCTCCAGAGTCGGGAGGGAAACTCAACCTTACTCACGGCTGGAAACGGTCCGGGATGAAAACAAGGAATGCCGACCGGTGAACTCCGTGGTACTACTCGGGTCGGCCTCTGCGCACAGGTGAATTCCCGGGGATCGACTCTGCGCGGCAGGAGTGCTGCAGACAGCCAGAAAAGGTTAGAAATAACTATTCATGAAAACACTGTTCAAACTCACGGAGACTAGAACCCGACTGGTAACCGTACGGACTGACAACGCGCCGGAGGACAGCTGGGGGTATTTAAGTCCGTCGGCGCTGATCACCGGAATTCCCCACAGCTGAGCCTGGGGAGAGGGGCGTGGTTGGAGATGGAACTCGGGGGTGGAAACTCTAGGCTGACACAAAAACAAAACATAAGCACATGGCCTTCAAACAACAACACTGACAGGGGGAAAACAGAGGAGCACACACTAGGTCAGACAGAGATCCTAACACAGCTAGTGAAGCTTCGCATGTAGTCTGCTATCTGATTGTGAGGTCAGGCTGATCTGATATCAGCTCTTTGGCTGCAGAATCTCCGCCAAAGTTCTCCTCTCAAACGGGTCTTGGAGGAGTGCTCGCGCTGGCGCTCGCGCGTAGAGACTTCTCCTGACGAGAACGCGCAGCGAGGGGGGCTTGATCGTTCTAGCCCCAAGCTGAATCTATTTGAGGGCGCTGATTGGCTAAATTGTATGTCACTCATTTATATCTTGAATCAGCAATTGGCTAAACTGCTTCATAGACCCGTCTCTTTTGGGCCAAAACGATCAAGCCCCCCTAGCTGCGCGTTCTCAGAGGTGTCGCGCACACAGGCAGAGAGCACGAGCAGAGAGCACACAGGCTGAGAGCACGAGCAGAGAGCACACAGGCGGAGAGGACGAGCAGGAAAAGCATATATTTTGCGCAGATATCATATTTTACACACATTACTGGGTACATTCCACAGTTCAAAAACACAAATATTGGCAATTTGTATAATTTATGATTATTATAATTTTTTTTTTTTTTTTTTTTTTTTTTTTGTGTGGCTCATGGTGGGGCCATGCCCCATGGCCCTCTATTGACCAGCCGCCACTG

GACACGGGGATGAAATGTACAATCGCTCCTGCAGCACGACTTCACTCTCAGCCCATCGGCAGCATTAAAGGTGACGTGTGAGGAACCGGCTCATCCTGTTCCAGGCTGCAGTTGATGTTTGCACTACATGACTTGTTTGGTTTTCAGTCTCACTGTGACAGACGCACCTGATGACTGAGCGTAAATGAAGTTATTGAGATAAAGTCTGACGCTGACGTCGTCTTTCACATCCAGCTCGAGTCTCTCTGACCTCCAACACGGCGGCTGAGTCTGCTCATCATTATTCCCGTCCTCCGTGCTTCTTTCCCCGTTTTCCTGTGTGTGCGTGTGTACACATATAGACGCCGAGATCGAAAAGTGTTCGATGGTGTGCCACTGTTGTTCAACAGTATGTACCCATCCCTGGGGTGGTATGCCAGTACCTTTCCAACGGCACACCGTTTCCTGAAGGCAGGTGCTGCTTGAAAGTTCATATTAGTGGTATGTGCCGTTGAATCAGAGTGGTAACCTATTAATGCTTGTTGTTTCCATCCTCTTCTCCCTTTTATCGCTGTCTCTCTTCTCTTCTTGCTGCTCTGTCGTTTCGGGCGGATGTTTGTCCAGGGTGCTGGATGAAGACTCCTACAAGCAGCCCTCCTCTGAGCTCATTGGTTATGTGTATGTTGCTCCAAATAACATCCTCCCTTCTCATGTTTTTAGATTTTTTCCCCCTCCGTTGTCATCAACTCCGAGCATCCAACCATTGTCATCATCTCATTACTAGTTAGGGCAGTACAGTTACCTCCCCTTGCATTTTTTAGTTCCATATTCATGTTTTAACTACTTTGATTTTGAATTGTTACATGCTTCACGTAGCTTTTTCCCTAGTTGCGCGTTTTCAGAGGTGTCGCGCACACAGGCAGAGAGCACGAGCAGAGAGCACACAGGTTGAGAGCACGAGCAGAGAGCACACAGGCGGAGAGGACGAGCAGGAAAAGCATATATTTTGCGCAGATATCATATTTTACACACATTACTGGGTACATTCCACAGTTCAAAAACACAAATATTGGCAATTTGTATAATTTATGATTATTATAATTTTTTTTTTTTTTTTTTTTTTTTTTTTGTGTGGCTCATGGTGGGGCCATGCCCCATGGCCCTCTATTGACCAGCCGCCACTGGACACGGGGATGAAATGTACAATCGCTCCTGCAGCACGACTTCACTCTCAGCCCATCGGCAGCATTAAAGGTGACGTGTGAAGAACCGGCTCATCCTGTTCCAGGCTGCAGTTGATGTTTGCACTACATGACTTGTTTGGTTTTCAGTCTCACTGTGACAAACGCACCTGATGACTGAACGTAAATGAAGTTATTGAGATAAAGTCTGACGCTGACGTCCTCTTTCACATCCAGCTCGAGTCTCTCTGACCTCCAACACGGCGGCTGAATCTGCTCATCATTATTCCCGTCCTCCGTGCTTCTTTCCCCGTTTTCCTGTGTGTGCGTGTGTACACATATAGACGCCGAGATCGAAAAGTGTTCGATGGTGTGCCACTGTTGTTCAACAGTATGTACCCATCCCTGGGGTGGTATGCCAGTACCTTTCCAACGGCACACCGTTTCCTGAAGGCAGGTGCTGCTTGAAAGTTCATATTAGTGGTATGTGCCGTTGAATCAGAGTGGTAACCTATAATGCTTGTTGTTTCCATCCTCTTCTCCCTTTTATCGCTGTCTCTCTTCTCTTCTTGCTGCTCTGTCGTTTCGGGCGGATGTTTGTCCAGGGTGCTGGATGAAGACTCCTACAAGCAGCCCTCCTCTGAGCTCATTGGTTATGTGTATGTTGCTCCAAATAACATCCTCCCTTCTCATGTTTTTAGATTTTTTCCCCCTCCGTTGTCATCAACTCCGAGCATCCAACCATTGTCATCATCTCATTACTAGTTAGGGCAGTACAGTTACCTCCCCTTGCATTTTTTAGTTCCATATTCATGTTTTAACTACTTTGATTTTGAATTGTTACATGCTTCACCTAGCTTTTTCTGTCTTTTTCAAATGTCAACCACAGTAATAATTATTCCCCCATATGTCAAATACATGACACACATTTCCAACAATTAACACTTACAATAATTGTCTGAAGTGTTTTGTTTCTACTTAACACCTTTTTATTTCTTTCCCCAAATTAATCTTCAAAATATATTATTCAGGAGTAGGATGCGTATAGATTTGTTTAAAGCAAGTGTGTTTTAATAATTGAACTTGGGTGTTTTTGCCATTAATAATATGATAAATTGAACTTATAGCTGCACTAATCAACATTTTGTAACAGTGCGTCACGATTGTGTAATATGAAAGAGGCCGGTGTGGTGGTGTGGTTTGATGCACCAGATGCAGACAACCGTGCACAGTCAAATGTGGGATGTGACGACATGTGCAGTCTTTCAGGTCAGATCACGGCAAATATAAGCACTTCCTGTTCTCTTTGAGATATGCACTATGGCAGGCTTGTAAAATGTCCTCACTCAATGGCAACATCTCTCATATGTGATATGAACATCACCATCTCTGTTTGAAAACAAATTCACCCCTGACAACTTTTCCTAGCACTTATTAAAATTGCGTTTGTAGTCTCTACCGAGATACATGTAGGCCTGCTGTATTTGAGGAAGGTTACAGTTTTTCGAGGGTGCTACGTGTGACCCTTTAATAGTCATAGTTTCTATGCAATTGAAGGCTATATGCAAACAGGTGCACAGTCTGTTTATAAGCATCATGGAAAAGTACAAGCATTTTGCACACACTCTGTATTTTTCCAATCGCTAACAATGTCATCGTGACATTGTGCTATTGGAAAGAGACCAGCTGATCTAGACAGTTGATATCATGATCAACAGCCCCAAACAAGTGCGCATGCGTGAGGAGTGATTGGCAGATGTATGAGAACTGAACCACGGGCTTAGGCATGCAAGAGTAGTCTTCTTGACTGAACTTGCGCTAGAGCTTCATTGGAGGCATCTATTTTGTCTTTTCTCGTATGATTTTGGGATGGACGCATGGGAGAGTTTTTCCCGTCTCAGCTTGCTTTTTACCCTAAATATTGTATACCTATTAGAACCGTTGTCACAGGGTTCAAATTAACATTTTCGTTTAGTTTTGATCATGATATATACGTTTTATCCATAAGGCATAGAACATGTGCATATACAGTAAGGGCTTGTTATACGACAGAAAGAAGAAGAGGATATGCGTGCAGGCTGTCAGCTAATAAATGGATCACAAGTTATAGAATGCAAGCTGGTGATAGTTTGCACAAAAACAAGTTATACTTTACTTATAAGAATATAAAATTTCCATTACAATTGGCATAAGGAGGTGTGATACAGTGACCTACTTTCAGGCCAATAGGAAACGGGATATGCCGGTTAAGTCCTCCCACATACTGTATATTAGATGCAGCACATGCACCTGTCCTGTCAGAAGTCTCAGCTACAGCTTTCACTTCGTTCTCCGCTAATTAATTAATTACTAATTAATTAAGTCTCAGCCACAGCCATGAAGTCAGTTGTTTTAACTGGTTTGCTGTTCGTCCTCCTTTGTGTCGACCACATGAGTTCAGCCAACAAGGTGAGATATTCTTGCTCCACAAAAGAATATTCAAAAATGTTAGCCACAGTAAAATTCAATTGTTTTCTGTTCAGAAAGACAGAGAACCTTTAAGAAACATTTTTGAAATAGTTTTTTCAACTGTGCCATGAGAACATTAATAATGTCTGACCTTTTATTTTCCATTCTTCAAGGCGTCCGTGGTGGCCAACCAGCTGATCCCCATAAATACTGCCCTGACTCTGATAATGATGAAGGCGGAGGTGGTCACCCCAATGGGCATCCCCGCCGAGGACATTCCCCGAATAATCGGAATGCAAGTGAACAGGGCAGTGCCGTTGGGCACAACCCTCATGCCAGATATGGTGAAAAACTATGAGAAGTAGTTCTGAGCGTGCCAAGGAGTTTCTTCCCAAAACCAAAAGAAGAAATGCCTCCTCTCACAATTAACCTTGTTTTTGTCACAAACCCAAGTCTGTCCGTATGTCAACTGAACATGTCAAAACCTGTGGAGACTGTTGAGATTTGATGTTCTGAAAAGATAAAGCCTATAAAATGTTGCCCAAATTTCCTGCCTGAGGTTTTTCCTTGTCTTTGATACATCGCTTTGCTGCTCGGATCGACTCACTCTGTGTATGCCACATTCACTTTGTACTCTCCTTCTCACGGTAGGTTTATTATTTTTAGATGTGCAGTTAGTTTCTGTGAAATAACATACCACACACTGATATTGTCTGTGCATTGACTTGGTGAGTGCACATTGTTTTTGATCTGGACATATTTATATTTGATTGATCAGGTGATGTGTGAATCTAAAGTGCTCCATACAGATGTTCTGCATTGAAAATATTCAATTGTGAGTAAATAGACGGCTTCATTCCAGTGGCGGCTGGTGGAAAATGTTCTAGGTAGGGCTGTGCAACATCCAATCAATTTCAGCAAACATCCCAGTATGATTTAATGCAAAGAACTAAGGTATCAAACATGCATCACTACTTTGCAGTTAAATATTTAACATTTAAACTAACACGAAAAAAAGCAGTAAATAACACTTTGACAAACACGTCAGGACATCTTATTTTTGTCACCCTCACAGGCAATTCAGGATACTATGATAATATCTATATATCATATTATAATATTCAGTATAATATAAAACACGCACGTATATATATATATATATTTAATTTAATATAATATAATAGAACATGTTTATTTATTGAGATGCCCCTATGGACCTTTATAGGAGGTAAAGATCAGGAGAAGTAAACATAAAGTGTAATTATGAATACTGGTGTTAAATTAAGCCATGATGAGTTTTCACTGTTAATTTACCATCTCTATTAAATGTTGATGCCTCCATGACCAAGTTAAGCAGATGAGACTCTAGAAGAAAAGATGTTCACTTTGCTGAATATGGCTAGCTTGACAGAGTTATCCATGGATATATAAATATATATATATGTATATATATATTTATTAAATGATTTATTTATAACTATATATCCATTTCTCAGACAGGTGCTTCATGTCCCTCACTTCCGTAACTGTCCATGCTGGATCTGTCCCCGTTGTTTAAAAAGCTGGGATCAGCTCTTTCACTGTAGTTTCTCCGCCAAAGTTCTCCTCTCAAATGGGTCTTTTGAGCTCGCGCTGCCTGTAGAGACTCGTTCTGACGAGCATGCGCAGTTTGGGGCAAAACAGATTGTGCTCATTGGTTAAATCTATTTGAGGACACTGATTGGCTAAATTGTATGTCACACTTTTATATCTTTATTCAGCAATTGGCTAAACTGCTTCATAGACCCGCCTCCTGTGAGAACTTCCTCCAGAGCCACAGCGCTGCTGAGACATGCAGACAGGCAGAGAGGACGTGCAGGAAAAGCAGATATTATGAGCAGATATCATATTTTACACATATTACTGGATGCATTCCATATTTCAAAAACACAAATATTGACAATTTGTATAGTTTTTTATTATTATTACTTTTTGTGGCTCATGGGGGGGGCGGCGCCCTAGTGCCCTCTATTGACAAGCTGCATGCTTATGGTTCATCAGACACCAATCCAAAACGTTCAGTATTTTATTGCCATATCAATCACAGTGCCTTCTGAATTTGTTTTGTTGGGGTCAACCACAAAAACGTATAATCACAGAGCACACAGAACAAAAACACACTGCTACAAGTACAATACAGTAAGCATACAATAGTGCAGCCAAATGGCTCACGCCCTTACAGTGACAAGTGGAAGTGCATGTGCTTGATTGCACAACCAGTGAAGCGAGGTATTCTAGTCTAAATACTGCGTAGTCTTTTACCCGCAGGTTGAAGGCTGCATAGAGGGTTTGCTGGGTGTGAGGAGTCCTTTTGAATTCACAAACCTCTACGCTGAATGCAGACATGGTATAGTTCAGAACTAGGGGAGCGTACACAGCCAACTGTTTTTAAAGTTCTGTCCATGAGTTTTTTTCTCGTAGGCTGTAGAGTTGCCATTGCACACAGTTATTGAGAAGTGAGCACTTTCTATTACCGCTCTGTAGAACTTTACCATCGCAACCTTTTACACATCAAATGTCCTAAGCTGTTGTTTATCGCCCCTCTTGAATGCATTATTTGTTCTTCCTGCATAGCATCTTAATTAACATGCAACTATTTACTAAATCAAATCTCAGTTTAACAAGGGGAAGATAGATAGATAGATATACACTTTATTCATCCCCAAGAAAAAAATTAGTTTGTAGCAGCAGCAATTTACAGAGTAAATAAAATCTGCAAGATGCTTTCTTAACAACACTTGCCACAGTTCAATTTAGAAGCTTATTTGGTGAGGACAGTACGAAAAGGTCTTTAGACATTTGGTCAGTTAATACTTTCTGCCAACACGTTTATATTTTGACACGTTAAGATTTCTGGTATTTCTTAGGGAAAACACAAATAGAAGTAATGTTGTACTTGAACCACTAGATAGAGCTGTTGTACAGCTGGATTTATCCAAGCGAGCGTTTCAACTCATCTCTGACTGAATACAGATAGGAACGTTGTTTTACCTCTCCTCAAAGGTTTCCATGGAGCATGGTTTCATCTGGACCCTGGTCCTTCCTCCTACCATGGCCCTGCTGACTCCAACTGCTAATATCACTTTTATTATTACTCACATTACTATTTCATACTACCGATACCATTAATGCACATTATTGACCTGCTTCTTCACCAGAGTCTCTGTGCTTTCTCGCCTCACAGGTTCCTGTGTATCCGGGTTTACCTGGACCAGGTGTTGCCTCCTGTCGTGGTAGTGTCATACATTGAATGTGCTGTAATTACATTGAATGTGTTGTAATTCACTATCCTGTTCTACACATGACATCATTGCACTTCTGTCCATCCTGGGAGAGGGATCCCTCCTCTATTGCTCTCCCACAGGTTTCTTCCTCTTTCCTCCCCGTTAAAGGGGTTTTTTGGGGAGTTTTTCCTGTTCTGATGTGAGGGTTCCGGGACAAGAGGATGTCAAATGTGTACATATTGTAAAGCCCTCTGAGGCAAATTTGTAATTTGTGATTTTGGGCTATACAAAAATAAATTGAATTGAATTGAATTGAATTGTTTAACTCCAGCCAGCCAGTATTGTAATGACCTGTAAACTGTTTGTTGTGTACATTAATGTCCCAAATGTCCAAAAACGCTCTCCCTTGTGGAATTAGTTTTGTATGTGAAGAGCTGATGTGTTACGGAACACCACAGATCTGTAACGAGGACTCAGAGCGACGTGTCGGTGAGGCTGCCTGGAGACTTCACGTCTTCTGGACGCCTTCGACCGAACGCTAACGCGTCTGTTCACGGCTCCACGGACGACACGATGCTGAAGATCAAACGCAGTAACGACGTTACCGGCTGACTCCTGCTTCCCTTCGTGTCCTATGAGTGCAATATCTGGGCCTTTGGAGGTTTTTACATTTTTAACGGCACAATAAAAACTAATGTATTCCTTTAGAGCGTCGTGTAGCTTGGCTGCATTTTAATAAAACAACATCTTCAAGTGATGTGTTCCTGTTGTTCGGGTCATTGTCTGGAGGGCTGGTGCATGTTTGGGTTTGTCTGTCATGTTAAACACTCAAACACGTCTTTAAACCTTTTATAATGTTTAAATGTCTTTTAAACTGGTAACGACACTAAAGAAGTTAAATCAAGCTGTTGCAGGAGGGAGACGGGAACGCAACACGAGTTATGAAACCAAAGGGAGATGTTTCCCGCTGGTAAACCTCCGACTTCACAGGCAGTTACGTCTCGTCAAAAAAAACACTCAGTGAGTTCAGGAGACGAATGAGTTCGACGTTTCACCGTGTTCACCACCAACACGCCGATTGTTGCATTAATCTCCAAAACACCAGAAGACGTACAAACTAAATGAACCGCGAAGAGTCAAACAGACCTAAATCAAACTCATCTCATATTCATGGACTCATTTATTACCTTTATATAAACCCTGTAAAGAAAGAAGTCAAGTACTAAATGAATTATACAGAAACAAAGGTTGAATATCTTCTGTTAAACTGTTTGATTTGGCTGATAAATATTTACTCAGATGGAGATTTTTAACGCTTCCTTCTCTGTGAAATCCTCAGACACGTGAACAGTGAACTATGAGACATTCACATCTGAAGAGATCCGAGCGCTGGTTAGAAACTCTAAACGTGCAGATCCAGCACCACGACCACCTGTGGAGCCTTCAAGCACGACCGACAGAAAATACAAGTCAGAGGAGCGACGATGGTCGACACACAACCGCGGTGAGAGCCGGAGATATGTCGTCATGGAAACATTAAAGTAAGTGCTGGGATGAAAGAACGTGGCCTCATGTGGTCCAGACCGCCTCCAGGATGTGGCCCGGACCGCCTCCAGGATGTGGTCCGGACCGCCTCCAGG

**Supplementary Figure S7. (a)** Phylogenetic tree of 30 AFP III. **(b)** Four groups of AFPIII with one, two, three, four ice binding domains (IBDs) are synthesized as monomer, dimer, trimmer, and tetramer AFPIII respectively. **(c)** Some IBDs show the ratios with the ω1(rates of non-synonymous substitutions (Ka) to the rates of synonymous substitutions (Ks))>1 (positive selection).

**
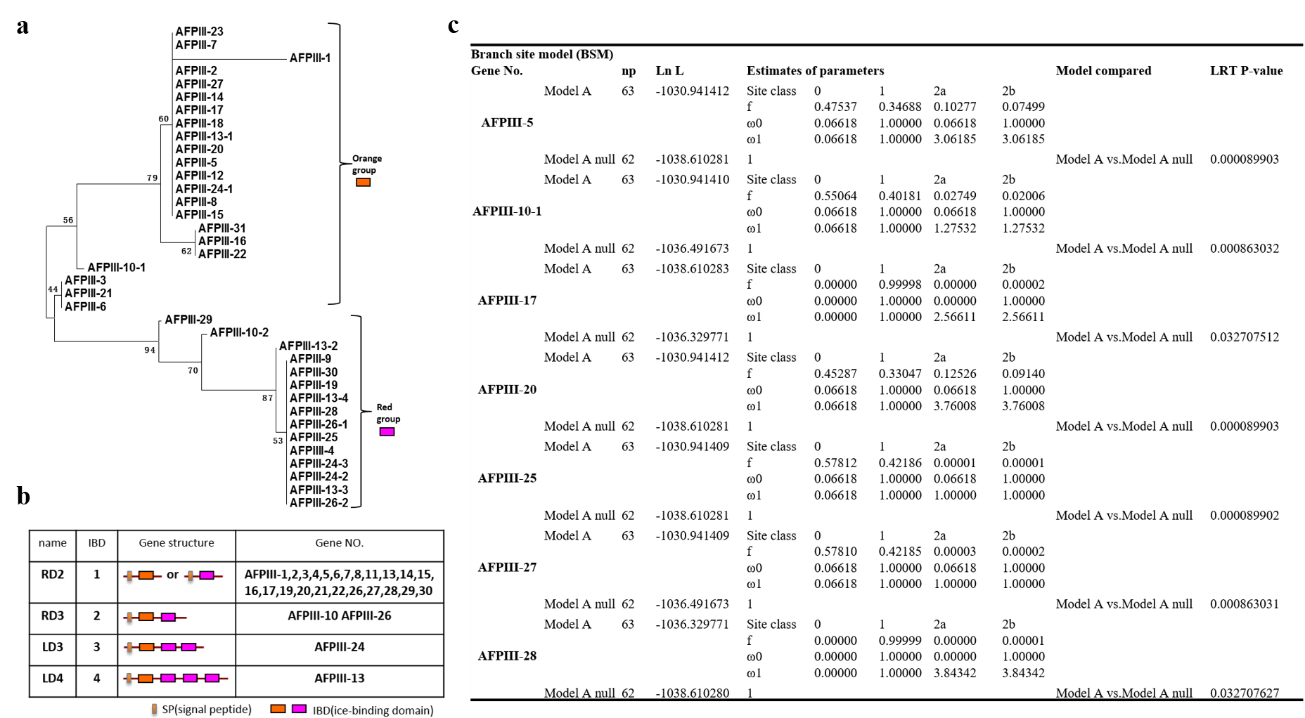
**

**Supplementary Figure S8.** Tandem 5S rRNA cluster. Repeat units with different colors represent different repeat units. Each vertical short line represents an SRS, and the ellipsis represents the variable copy number of SRS.


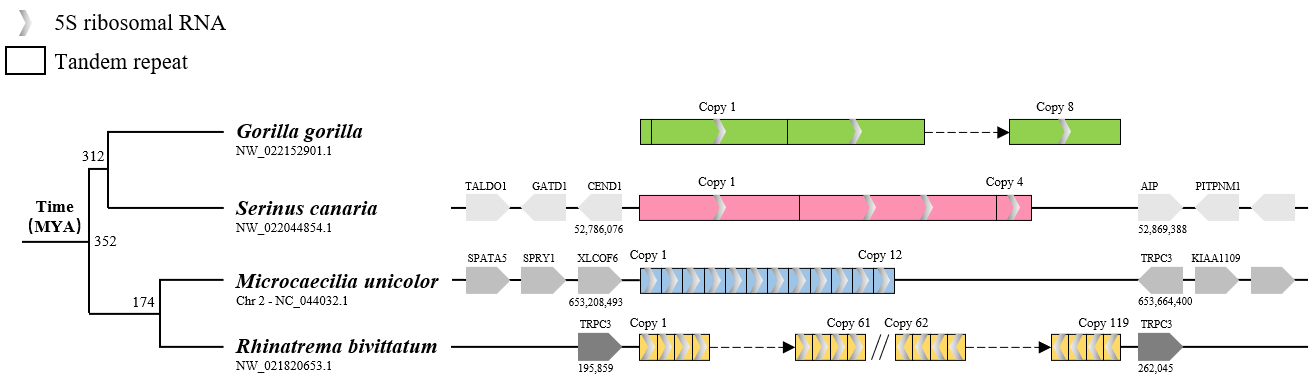


**Supplementary Figure S9.** The subspecies of *Xenopus* have high-speed rail model sequences whose repeat unit encodes interferon alpha-1. Each vertical short line represents an SRS, and the ellipsis represents the variable copy number of SRS. SRS and repeat units with different colors represent different SRS and repeat units, respectively.


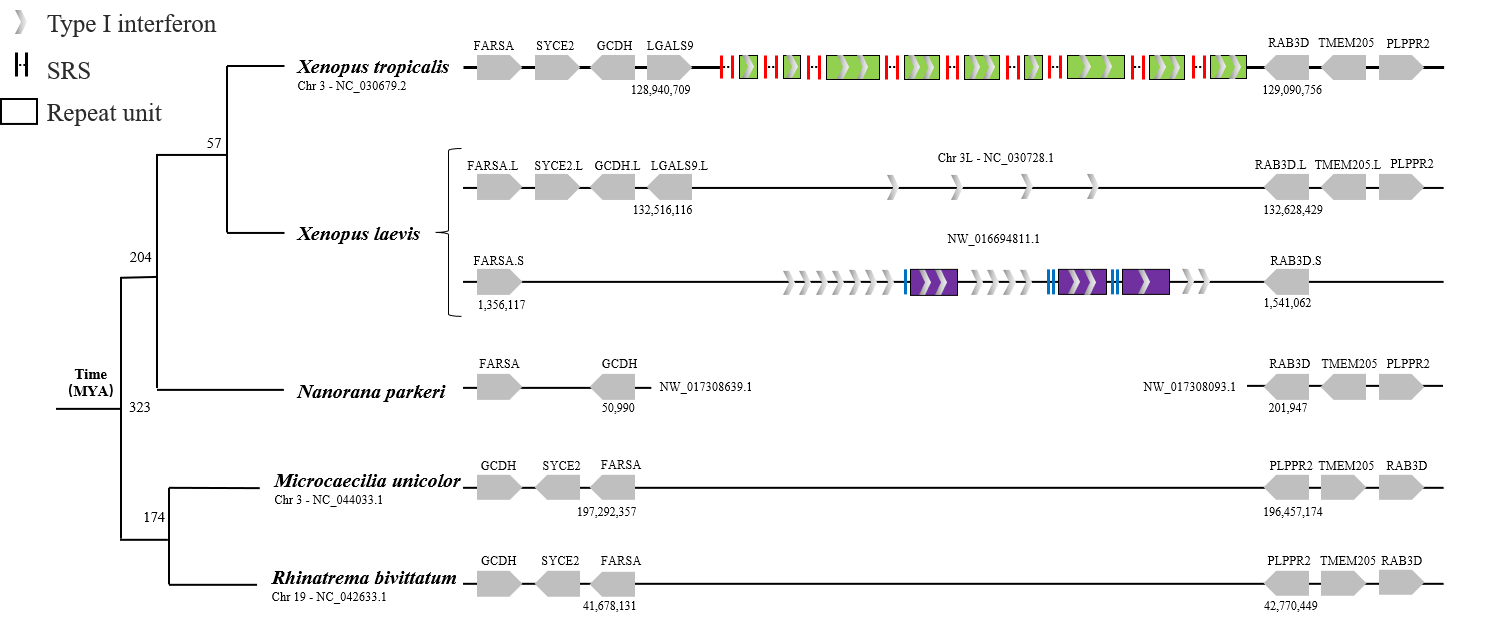


**Supplementary Figure S10.** Alignment of high-speed rail model sequences of different ecotypes of *Arabidopsis thaliana* between long-range PCR and NCBI.

(a) High-speed rail model sequences of Ws-0_seq.

TTTTGTGTTTCACGAGCGAACTAAACACTAGAGCGTGATTGTCACAAGGTCAGAGAATGTATTGAAGCTGGGATTCTCAAGACTATGTTTGTTCGATCAGATAATCAGCTTGCAGACATGTTTACTAAACCTCTTTATCCGGTGCTCTTCAGAACTAACAATAGCAAGCTTGGAGTTCTTAACATCTTTGAAAACTCAAGCTTGAGGGGGGCTATTAGACTGTATATATAGATTCGGTTTAGACCGGTTAACTTAGATTGTATTTACAATGTATATGGTTTAGATTGGTTTAGTGTAGAGTATATAAACATCCAATTGCACATTCTGTTTCGATTGAGAAATATAGAAAAGTTTCACATATCTCTTTGATTCATGTTCTCTATCAGTATTCGCTTGCAAAGTCGAAAGTATTTCAACACTTTTACCTTGACGGTTTTGTCATCGTACATGATCCAACAATCCACATATATAACTCAACTGAAATGGAATTAGGGCATCAATTGGATAAAGAAAACTATGAGTACAGATTTATATCCCCAATGAATACATGAAGGAACCATGGTGAAATATCATTGAGGATCTCGTTCCTGTTTCTGGCTTACTTCCGGATGCACTAACCTTTCCAACTCATGCGCTCACTTGATCACCTCTTCTCTTGATTTGGTCAGCATGAAGATCTTGTTTTATCACCATGCTCTGTGATTTTTCATAACCACACAGAAGATAATGAATCAAATTATGTAAGACCTAAGGAAATGAAAGTAGCTTCAAACCGAAGGAAATGAGAAGAATGAATGCTTGAGAAGCGTACCTAAATTCTGCATCAAAAAGGACAATTGTTCATCAAAAAGGTTAGGGAAAAACCTTCGGTGATGCACTTAAGGGAGATGTGGAGAGGTGTCGTTTATGAGATAAATGGTAGGAAGGGGAAACGATTGAACTTTCTTGGACATTATTGAAAACCCTAATTTCTTAGACTAAAGATTCTATGTCGAGAGAAGTCTAAAAGTTAATGACGTGGCTGTACTTGGTAAGAATTTTGAGTTAGAAAGAGAGTAATATGACGTGGCTTTTTCATTAAGTTAACTCAATAATACATAAATATTGTTTCATGTCGTTAATTTACTGAATGTATAATTATGAATTTGCTGATGTGGCATGATTTTAGGTAGTCTTGAATTTGCTGATGTAGATGGTTTGAGAAGCCACAATGAGCTTCTTTTATTAGTAAGAGATTGGTTTAGGAATATTTTCAAAGAGAAATTTCTAACAAAATCTTACATGTATGTTTCTTATCTCCTACCGCTACCACCGACTTTCCTAGATTTATTAGCACGTGACACTTTTAGGACACAAGGAATTGAACATGCATTTGTTAGAACCGTTGAATAACAATTCATAAATTATTTTTAAGTACTACATAATTTGTTTCCAGTGGGAAAAGTGATTAAAAAATAGAAAAAATAGAAAAAGGCGCCATTAAAAGTAGTAACCAACATATACTGTAACCGATGACTCAAAAACTTCGTTCTCTTAACAGGTGTGGGTTTCCTCGCAATTGTCTTGCAAATTTGCGAGGGAAATATTCGTCGCAAAGTTTGCGACGGAATAGCGAGTTGGTCCTGTTCTC

N1=4

TCGGAGAGATCTATTCGTCGGGGAAGAAGAGGTTTG

TCGGAGAGATCTATTCGTCGACGAATTCGAGGTTTG

TCGGAGAGAACAATTCATCTGCGAATTTGAGTTTTA

TCGGAGAGAACAATTCGTCGGCGAAAACGAAGAGGTTTG

TC

AAAATGAAACTGAAACCTTAATTTTTCAGTAAGGGTAAGATCGTTATTTACCACATTAGAAGGGGCAGTTTTAAAAATGGCCATCAGGAAAGGGCATTGTTGCAAAAGGTTATTAGAAAAGGCACTTTTGCAAATCTCCCTTTTGATTGTTATATCTAAGATTTCACCTGTACAATATCATCCCCTATTAATATCGACCCAAATCATTCTAACCATATAACCCCGTCCTATGAAATTAAACATTATTTTGTCTTAGTTTTATATAAACTTCAAATATTTATAATAATAAAACTAACGAATGTTACTGTAAATGATAACCAAAACATTAAACAAATAGTAAGGAAATTTAAAACGTGTTAACCTAACCCGAGAAGACTCGCAAACTCCGTCGCAAAAATTGCTACAGAATAAGTTATCGTCGCAAGTGCGTCGCAATTTGCGGGGGAACTCAGTTTCCCGCAAAGCCCCTCGCAGACTTGCGACGTTTTGCGGGGAATCATGGCTTCTACGCAGTTTCCTCGCAATTGTCTCGCAAATTTGCGAGGGATA

N2=4

TATTCGTCGTAAAGTTCGCGACGGAATAGCGAGTTGG

TCCTGTTCCCTCTATTTGTCGGCGAATAAGGGGTTTG

TCGGAGAGAACAATTCGTCGGCGAATTTGAGTTTTA

TTGGAGAGAACAATTTGTCGGCGAAAACAAAGAGGTTTG

TC

AAAATGAAAGTGAAACCCTAATTATTCAGTAAGGGTAACACCGTTATTTCCCATAATAGAAGGGGCAGTTTTAAAAATGGCCATTAGGAAAAGGCATTGTTGCAAAAAGTTATTAGAAAAGGGAACTTTCGCAAATGTCCCTTTTGATGGTTATATCTAAGATTTCACCTGTACAATATCATCCCCTATTAATATCGACCCAAATCATTCTAACCATATAGCCCCGTCCTATGAAATTAAACATCATTTTGTCTTAGTTTTATATAAACTTCAAATATTTATAATAAGAGAATTAACGAATGTTACTGTAAATGATAACCAAAACATAGAACAAATAGTAAGGAAATTTAAAACGTATTCACCTAACCCGTGAAAACTCGCAACTCCATCGGAAAATTTGCTACAGAATAAGTTATCGTCGCAAGTGCGTCGCAATTTGCGGGGGAACTCAGTTTCCACGCAATTGTCTCGCAAATTTGCGAGGGCAATATTCGTCACAAAGTTTGTGACGGATTAGCGAGTTGGTCCTGTTCCC

N3=5

TCGGAGAGATCTATTCGTCGGCGAAGAAGAAGTTTG

TCGGAGAGATCTATTCGTCGGCGAAGAAGAGGTTTG

TTGGAGAGATCTATTCGTTGGCGAATTTGAGGTTTG

TCGCAGAGAACAATTTGTCGACGAATTTGTGTTTTA

TTGGAGAGAACAATTCGTCGGCGAAAACGGAGAGGTTTG

TC

AAAATGAAAGTGAAACCCTAATTATTCAGTAAGGGTAAGACCGTTATTTTCCACATTAGAAGGGGCAGTTTTAAAAATGGCTATCAGGAAAGGGCATTATTGCAAAAGGTTATTATAAAATGGCACTTTTGCAAATCTCCCTTTTGATTGTTATATCTAAGATTTCACCTGTAGAATATCATCCCCTATTAATATCGACCCAAATCATTCTGACCACATAACCCCGTCCTATGAAATTAAACATCATTTTGTCTTAGTTTTATATAAACTTCAAATATTTATAATAAAAGAACTAACGAATGTTACTGTAAATGATAACCAAACATAGAACAAATAGTAAGGAAATTTAAAACGTGTTCACCTAACCCGCGAAGACTCCCAACTCCGTCGGAAAATTTGCTACAAAATAAGTTATCGTAGCAAGTGCGTCGCAATTTGCGGGGGAACTTAGTTTCCTCGCAATTGTCTCGCAAATTTACGAGGGAAATATTCGTCGCAAAGTTCGCGACAGAATAGCGAGTTGGTCCTGTTCCC

N4=4

TCGGAGAGATCAATTCGTTGGCGAAGAAGAGGTTTG

TCGGAGAGATCTGTTCATCAGCGAACTTGAGGTTTA

TTGGAGGCAACAATTCATCGGCAAATTTGAGTTTTA

TTGGAGAGAACAATTCGTCGGCGAAAAACAAAGAGGTTTG

TC

AAAATGAAAGTGAATCTCTAATTATTCAGTAAGCGTAAGACCGTTAGTTTCCACATTAGAAGGGGCAGTTTTAAAAATGGCCATTTGGAAAAGGCATTGTTGCAAAAGGTTATTAGAAATTGGCACTTTTGCAAATCTCCCTTTTGATAGTTTATATCTAAAATTTCACCTGTAGAATATCATCCCCTATTAATATCGACCCAAATCATTCTGACTATATAACCCCATCCTATGAAATTAAACATCATTTTGTCTTAGTTTTATATAAACTTCAAATATTTATAATAAGAGAACTAACGAATGTTACCGTAAATAATAACCAAAACATAGAACAAATAGTAAGGAAATTTAAATTGTGTTCACCTAACTCGCGAAGACTCGCAACTCCATCGGAAAATTTGCTAGAGAATAAGTTATCGTCGCAAGTGCGTCGCAATTTGCGGGGGAACTCTGTTTCCCGCAAAGCCCTCGCAAATTTGCGATGTTTTTGCGGGAATCATGACTTCCACGCAGTTTCCTCGCAATTGTCTTGCAAATTTGCGAAGGAAATATACGTCGCAAAGTTTGCGACGGAATAGCGAGTTGGTCATGTTCCC

N5=4

TCGGAGAGATCTATTTGTTGGCGAAGAAGAGGTTTG

TCAGAGAGATCTATTCCTCGGCGAATTTGAGGTTTG

TCGGAGAGAACAATTCGTCGGCGAATTTGAGTTTTA

TCAGAGAGAACAATTCGTCGACGAATTTGAGTAGGTTTG

TC

AAAATGAGAGTGAAACCCTAATTATTCAGTAAGGGTAAAACCATTATTTTCCCACACTAGAAAGGGCAGTTTTAAAAATGGCCATCAGGAAAGGGCATTGTTGCAAAAGGTTATTACAAAAATGGCACTTTTGCAAATCTCCCTTTTGATTGTTATATCTAAGATTTCACCTGTACAATATCATCCCCTATTAATATCGACCCAAATCATTCTGACCATATATCCCCGTCCTATAAAATTAAACATTATTTTCTCTTAGTTTTATATAAACTCAATTATTTATAATAATTAAATTAACGAATGTTACTGTAAATGATAACCAAAACATTAAACAAATAGTAAGGAAATTTAAAACGTGTTCACCTAACCTGCGAATACTCGCAACTCCATCGGAAAATTTGCTACAAAATAAGTTATCGTCGCAAGTGCGTCGCAATTTGCAGGGGGTCTCAGTTTCCTCGCAATTGTCTTTCAAATTTGAGAGGAAAATATTCGTCGCAAAGTTTGCGACGGAATAGCGAGTTGGTCCTGTTCCC

N6=5

TCGGAAAGATCTATTCGTTGGCGAAGAAGGGGTTTG

TCGGAGAGATCTATTCGTCGGCGAAGAAGAGGTTTG

TCGGATAGATCTATTCGTCGGCGAATTTTAGGTTTG

TCGCAGAGAACAATTCGCCGGCGAATTTGAGTTTTA

TTGGAGAGAACAATTCGTCGGCGAAAACAAAGAGGTTTG

TC

AAAATGAAAGTGAAACCCTAATTATTCAGTAGGGGTAAGACCGTTATTTTCCATATTCGAAGGGACAGTTTTAGAAATGGCCATCAGAAAAGGGCATTGTTGCAAAAGGTTATTACAAAAATGGCGTTTTTTGCAAATCTCCCTTTTGATTGTTATATCTATGATTTCACCTGTAGAATATCATCCCGTATTAATATCGATCCAAATCATTCTGACCATATAACCCCGTCCTATGAAATTAAACATTATTTTGTCTTAGTTTTATATAAACTTCAAATATTTATAATAAGAGAACCAACGAATGTTACTGTAAATGATAACCAAAACATAGAACAAATAGTAAGGAAATTTAAAACGTGTTCACTTAACCGGCGAAGACTCGCAACTCCATCGAAAAATTTGCCACAAAAGTATGTTATCGTCGCAAGTGCGTTGCAATTTGCAGGGGAACTCAGTTTCCCGCAAACCCTCCGCAAATTTGCGACGTTTTTGCAGGGAATCATGGCTTCCACGTAGTTTACTCGCAATTGTCTCGCAAATCTGCGAGAGAAATATTCGTCGCAAATT

N7=6

TGCGAGGGAAATACTCGTCGGCGAAGAAGAGGTTTG

TCGGAGAGATCTATTCGTCGGCGAAGAAGAGGTTTG

TCGGAGAGATCTATTCGTCCGGCGAAGAAGGAGGTTTG

TCGGAGAGAACAATTCGTCGGCGAATTTGAGTTTTTA

TCGGAGAGAATAATTCGTCTGAAAATTTTAGTTTTA

TCGGAGAGAACAATTCGTTGGCGAATTTGATTAGATTTG

TC

AAAATGAAAGTGAAACCCTAATTATTCAGTAAGGGTAAGACCGTTATTTTCGACATTAGAAGGGGCAGTTTTAAAAAAGGTCATAAGGAAAGGGCATTGCTGCAAAAGGTTATTATAAAAAGGGCACTTATACAAATCTTCCTTTTGATTGTTATATCTAAGATTTCATATGTAGAATATCATCCCGTATTAACATAGACCCAAATCATTCTGACCATATAACCCTGTCCTATGAAATTAAACATCATTTTGTCTTGGCTTTATAAACTTCAAATATTTATAATAAGACTCGCAATCATAGACCCTAACCCGCGAAGACTCGCAACTCCGTCGCAAAAATTGGTACAGAATAAGTTATCGTCGCAAGTGCGTCGCAATTTGCGGGGGAACTCAGTTTCCCGCAAAACCCTCACAAATTTGCGACGCTTTCGCGGGGAATCATGACTTCCACGCAATTTCCTCGCAATTGTGTAGCAAACGTGCGAGGGAAATATTCGTCGCAATATTTGCGACGGAATAGCGAGTTGGTCGTGTTCCC

N8=5

TCCGGGAGATCTATTCGTCGGCAAAGAAGAGGTTTG

TCGGAGAGATCTATTCGTCGGCGAAGAAGAGGTTTG

TCGGAGAGATCTATTCGTCGGCAAATTTGAGGTTTG

CGGAGTGAACAATTCTTCGTCGAATTTGAGTTTTAT

CAAAGGGAATAATTCGTCGGCAAATTTGAGTAAGTTTG

TC

AAAATGAAAGTGAAACCCTAATTATTCAGTAAGGGTAAGACCGTTATTTTCCACATTAGAAGGGGCAGTTTTAGAAATGGCCATCAGAAAATGGCATTGTTGCAAAAGGTTATTAGAAAAGGGCACTTTTGCAAATCTCCCTTTTGATTGTTATATATCTAAGATTTTACCTGTAGAATATCATCCCCTATTAATATCGACCCAAATCATTCTGACCATATAACCCTGTCCTATAAAATTAAACATCATTTTGTCTTAGTTTTATATAAACTTCAAATATTTATAATAAGAGAACTAATGAATGTTACTGTAAATGATAACCAAAACATAGAAAAAATAGTAAGGAAATTTAAAACGTGTTCACCTAACCCGCGAAGACTCGCAACTCCATCGGAAAATTTGCTACAGAATAAGTTATCGTCGCAAGTGCGTCGCAATTTACGGGGAAACTCAGTTTCCCGCAAAGCCCTCGCAAATTTGCAAAGTTTTTGCGGGGAATCATTGTCTCGCAAATCTGCGAGGGAAATATTCGTCTCAAAGTTTGCGATGGAATAGCGAGTTAGTCATGTTCCA

N9=5

TCGGAGAGATCTATTCGTCGGCGAAGAAGAGATTTG

TCAGAGAGATCTATTCGTCGGTGAAGAAGAGGTTTG

TCGAAGAGATCTATTTGTCGGCGAATTTGAGGTTTG

TCGGAGAGAATAATTCATCGGCGAATTTGAGTTTTA

TCGGACAGAACAATTCGTCGGCGAATTTGAGTAGGTTTG

TC

AAAATAAAAGTGAAACCCTAATTATTCAGTAAAAGGTAAGACCGTTATTTTCCACGTTCGAAGGGGCAATTTTCAAAAGGCTATCAGGAAAGGGCATTGTTGCAAAAGATTATTAGAAAAATGGCACTTTTGCAAATCTCCCTTTTGATGTTTGTATCTTAGATTTCACCTCTAGAATATCGTCTCGTATTAATATCGACACAAATCATTCTAACCATATAACCCCGTCCTATGAAATTAAACATCATTTTGTCTTAGTTTTATAAACTTGAAATATTTATAATAACACTCGCATCCTAGACCCTAACCCGCGAAGACTCGCAACTCCGTCGCAAAAATTGCTACACAATAAGTTATCGTCGCAAGTGCTTCGCAATTTGCGGGGGAACTCAGTTTCCCGCAAAGGCCTCATAAATTTGCTACGTTTTTGTCGGCGAATTTGAGGTTTGTCGGAGAGAACAATTCGTCGGCGAATTTGAGGTTTGTCGGAGAGAACAATTCGTCGGCGAATTTGAGTTTTATTAGAGAAAAAGATTCGCCGGCGAAAACGAAGAGGTTTGTCAAAGTGAAAGGAAAACCCTAATTATTCAGTAAGGGTAAGACCGTTATTTTCCTTATTAGAAGGGGCAATTCTAAAAATGGCCATTAGGAAAGGGCATTGTTGCAAAAGATTATTAGAAATAGGGCACTTTTGCAAATCTCCCTTTTGATTGTTATATCTAAGATTTCACCTGTAGAATATCATCACGTATTAATATCGACCCGAATCATTCTGACTATATAACCGCGTCTTATGAAATTAAATATCATTTTGTCTTAGTTAAAAAACTTCAAATATTTATAATAAGACTCGCAAACCCTAAACCCTAACCCGCGAAACTCGCAACTCCGTCGCAAAAATTGCTACAGAATAAGTTTTCGTCGCAAGTGCGTCGCAATTTGCGTGGGAACTCAGTTTCACGCAAATCCTTTGCAAATTTGCGACGTTTTTGCGGGGAATCATGACTTCCACGCAGTTTCCATGCAATTGTCTTGCAAATTTGCGAGGGAAATATTCGTCGCAATGTTTGCGACGGAATAGCGAGTTGGTCCTGTTCCCCTCGCAAACCTCTCGCAACTCTGTAACAATTTGCAAGGGATGTTTTCCATCGCAAATTTTTCTCGCAAATCGCATGTTTTCTTGTTGTCGAAAAAAAAAAAAAATCTAAACTATTTGCTTCATCATCGCTCTTCCTACAAACCAAACTGGCTTGAAAAAAGCTGTCATTGTTTTCATCATGTGTTATCCATCATTATCTGTCCATCTATATGTTTATATTTTGATAAAACTTGTATCTTTTGATATCATTTACATTGATTTCACTTGTTGGACACATAGAAGTGTAGAGAAATAATTTGCATCCCTTGATTTAGTGGACATAATACTGTGGAACTCAATTTGCGAGGAACTCAGTTTCCCGCAAAGCTCTCGCAACTCTGTAGCAATTTGCGAGGGATGTTTTCCATCGCAAATTTCCCTCGCAAATCGTCTGTTTTCTTGTAGCGAAAAAGAAAAAATCTAAACTATTTGCTTCATCATCGCTCTTCCTACAAAACAACCTGGCTTGAAAAGAGGTGTCATTGTTTTCATAATGTGTTATTTTATCATTATCTGTCCATCTATATGTTTATATTTTGCTAAACTTGTATCTTTTGATATCATTTACATGGATTTCACTTGTTGGACACATAGAAGTGTAGAGAAATAATTTGCATTCCTTGATTTAGTGGACATGATATTGTGGATACTATCTAACTTGCAGAAACTATTTCTTATATTTTTTCAGATCAGTTTCTTGTTTAAAGGGGATCAAGTCAAACCACGGCATGGACTCTTCTACGGTCAATACATTCTCTCTCTGGAACATTAAAGACATAAACACCATTTAGATTATGAAGATTATTTTTTTACGCTACAAGTGACAACAAAATTGTTAGCAAATAAAGAAGTAATGACCCCCAAAAAAAGAAAGAAAACAATACAACACGTTTCATCGAGAAACTTACAGTGTTAAAGAAACATATAGTTATTTCATCATTTGACTGATTGATAATATGAGTTTTGAGTGACTGAGCAATGCAACTAACATCAATGTGAAAATGACTTTTTGTTTGTCTTCTTTCTCTTGTCAAAAATCAATATAATACAAAAGGGCAAGTAGAGCTGAACATTTTGGGAGAAGCATCAATTAGATAGACTACAAATTCTTTGGAATTCTATGGAAGCCTCTTTTTCCTGTTGCACCAAAAGATTCATCACTAACCAATACTAATCAATGTTTCATTTCTTTTTTGGCTTTCACTAAAAAGTCCAAGAAGAAAAAAAGGTCAAAACTGAAATATATTATTTACATCAAACATAATCAATATAACCGGTTAAGCAATTAATGATACAGAAGACTCATCAATGGACAACTATGATATCATTTACATGAATTTGACTTGTTGGACACACAGAAGTGGACAACAATAGTTTGCAATCCTTGATTTAGTGGACATGATATTGTGGATACTATGTAATTTGCATAAACTATTTTTTTTATTTGGGTGGATATAAGGACAACTATGATATCATTTACATGGATTTGACTTGTTGGACACATAGAAGTGGACAACAATAGTTTGCAGTCCTTGATTTAGTGGACATGATATTGTGGATACTATCTAATTTGCGTAAACTATTTCTTCTATTTTTTTAGATCAGTCTCTTGTTTAATGGGGATCAAGTCAAACCACAGCATGGACTCTACTATTGTTGATAAAAAATTTTATTTTTAGAAAGCAACATGACTTTTCCTTTCTCAATTCTAGCCCATTATGATTTATGTACATAGATATCTTATATTTTTTCTGCTAACGATGCAGAATGCAGTTGAAATGGCAACATTCATCTGATTCATGGTAAGGCCACAGTCTAAATTATGTGCATCAATCCTTAAATCAGGTATGTAATGTTATTCAGAAAAAAACTTTCATTTAATATTTCGGTGGGGAATAAAAGAAATTTTTTGCTTTATGAAGAACCATACGATGATATAAAGCCGACTAAAGTCTGAGTGCGTTGGAAAAGTACACAATAATTTGCAGCAACACACTATCTCTAATCGTCACTCTCGTTAGTCTAAACTTTTCAGATATCTACAAGTTCCATAATCTTAAGCTTATCAAAGTCTATGATAAAACATCGCTCTGCCAGGTCAATACGCAGCAGCTGCTGAGTATCGGGCAATGGCTGTTGCCATCGCTCTGCCAGGTCAATACATCGCTCTGCCAGGTCAATAC

(b) High-speed rail model sequences of Ws-1_seq.

TTTCAAAGCATCGGCTTACTTATATTGTGATAATGAAGCAGTCTTGCACATTGTTAGCAATTTTGTGTTTCACGAGCGAACTAAACACTAGAGCGTGATTGTCACAAGGTCAGAGAATGTATTGAAGCTGGGATTCTCAAGACTATGTTTGTTCGATCAGATAATCAGCTTGCAGACATGTTTACTAAACCTCTTTATCCGGTGCTCTTCAGAACTAACAATAGCAAGCTTGGAGTTCTTAACATCTTTGAAACTCAAGCTTGAGGGGGGCTATTAGACTGTATATATAGATTCGGTTTAGACTGGTTAACTTAGATTGTATTTACAATGTATATGGTTTAGATTGGTTTAGTGTAGAGTATATAAACATCCAATTGCACATTCTGTTTCGATTGAGAAATATAGAAAAGTTTCACATATCTCTTTGATTCATGTTCTCTATCAGTATTTGCTTGCAAAGTCGAAAGTATTTCAACACTTTTACCTTAACGGTTTTGTCATCGTACATGATCCAACAATCCACATATATAACTCAACTGAAATGGAATTAGGGCATCAATTGGGTAAAGAAAACTATGAGTACAGATTTATATCCCCAATGAATACATGAAGGAACCATGGTGAAATATCATTGAGGATCTCGTTCCTGTTTCTGGCTTACTTCCGGACGCACTAACCTTTCCAACTCATGCGTTCACTTGATCACCTCTTCTCTTGATTTGGTCAGCATGAAGATCTTGTTTATCACCATGCTCTGTGATTTTTCATAACCACACAGAAGATAATGAATCAAATTATGTAAGACCTAAGGAAATGAAAGTAGCTTCAAACCGAAGGAAATGAGAAGAATGAATGCTTGAGAAGCGTACCTAAATTCTGCATCAAAAAGGACAATTGTTCATCAAAAAGGTTAGGGAAAAACCTTCGGTGATGCACTTAAGGGAGATGTGGAGAGGTGTCGTTTATGAGATAAATGGTAGGAAGGGGAAACGATTGAACTTTCTTGGACATTATTGGAAACCCTAATTTCTTAGACTAAAGATTCTATGTCGAGAGAAGTCTAAAAGTTAATGACGTGGCTGTACTTGGTAAGAATTTTGAGTTAGAAAGAGAGTAATATGACGGGGCTTTTTCATTAAGTTAACTCAATAATACATAAATATTGTTTCATGTCGTTAATTTACTGAATGTATAATTATGAATTTGCTGATGTGGCATGATTTTAGGTAGTCTTGAATTTGCTGATGTAGATGGTTTGAGAAGCCACAATGAGCTTCTTTTATTAGTAAGAGATTGGTTTAGAAATATTTTCAAAGAGAAATTTCTAACAAAATCTTACATGTATGTTTCTTATCTCCTACCGCTACCACCACTTTCCTAGATTTATTAGCACGTGACACTTTTAGGACACAAGGAATTGAACATGCATTTGTTAGAACCGTTGAATAACAATTCATAAATTATTTTTAAGTACTACATAATTTGTTTCCAGTGGGAAAAGTGATTAAAAAATAGAAAAAATAGAAAAAGGCGCCATTAAAAGTAGTAACCAACATATACTGTAACCGATGACTCAAAAACCTCGTTCTCTTAACAGGTGTGGGTTTCCTCGCAATTGTCTTGCAAATTTGCGAGGGAAATATTCGTCGCAAAGTTTGCGACGGAATAGCGAGTTGGTCCTGTTCTC

N1=4

TCGGAGAGATCTATTCGTCGGGGAAGAAGAGGTTTG

TCGGAGAGATCTATTCGTCGATGAATTTGAGGTTTG

TCGGAGAGAACAATTCATCTGCGAATTTGAGTTTTA

TCGGAGAGAACAATTCGTCGGCGAAAACGAAGAGGTTTG

TC

AAAATGAAACTGAAACCTTAATTTTTCAGTAAGGGTAAGATCGTTATTTACCACATTAGAAGGGGCAGTTTTAAAAATGGCCATCAGGAAAGGGCATTGTTGCAAAAGGTTATTAGAAAAGGCACTTTTGCAAATCTCCCTTTTGATTGTTATATCTAAGATTTTACCTGTACAATATCATCCCCTATTAATATCGACCCAAATCATTCTAACCATATAACCCCGTCCTATGAAATTAAACATTATTTTGTCTTAGTTTTATATAAACTTCAAATATTTATAATAATAAAACTAACGAATGTTACTGTAAATGATAACCAAAACATTAAACAAATAGTAAGGAAATTTAAAACGTGTTAACCTAACCCGAGAAGACTCGCAACTCCGTCGCAAAAATTGCTACAGAATAAGTTATCGTCGCAAGTGCGTCGCAATTTGCGGGGGAACTCAGTTTCCCGCAAAGCCCTCGCAGACTTGCGACGTTTTGCGGGGAATCATGGCTTCTACGCAGTTTCCTCGCAATTGTCTCGCAAATTTGCGAGGGATA

N2=4

TATTCGTCGTAAAGTTCGCGACGGAATAGCGAGTTGG

TCCTGTTCCCTCTATTTGTCGGCGAATAAGAGGTTTG

TCGGAGAGAACAATTCGTCGGCGAATTTGAGTTTTA

TTGGAGAGAACAATTTGTCGGCGAAAACAAAGAGGTTTG

TC

AAAATGAAAGTGAAACCCTAATTATTCAGTAAGGGTAACACCGTTATTTCCCATAATAGAAGGGGCAGTTTTAAAAATGGCCATTAGGAAAAGGCATTGTTGCAAAAAGTTATTAGAAAAGGGAACTTTCGCAAATGTCCCTTTTGATGGTCTATATCTAAGATTTCACCTGTACAATATCATCCCCTATTAATATCGACCCAAATCATTCTAACCATATAACCCCGTCCTATGAAATTAAACATCATTTTGTCTTAGTTTTATATAAACTTCAAATATTTATAATAAGAGAATTAACGAATGTTACTGTAAATGATAACCAAAACATAGAACAAATAGTAAGGAAATTTAAAACGTATTCACCTAACCCGTGAAAACTCGCAACTCCATCGGAAAATTTGCTACAGAATAAGTTATCGTCGCAAGTGCGTCGCAATTTGCGGGGGAACTCAGTTTCCACGCAATTGTCTCGCGAAATTTGCGAGGGCAATATTCGTCACAAAGTTTGTGACGGATTAGCGAGTTGGTCCCTGTTCCC

N3=5

TCGGAGAGATCTATTCGTCGGCGAAGAAGAAGTTTG

TCGGAGAGATCTATTCGTCGGCGAAGAAGAGGTTTG

TTGGAGAGATCTATTCGTTGGCGAATTTGAGGTTTG

TCGCAGAGAACAATTTGTCGACGAATTTGTGTTTTA

TTGGAGAGAACAATTCGTCGGCGAAAACGGAGAGGGTTTG

TC

AAAATGAAAGTGAAACCCTAATTATTCAGTAAGGGTAAGACCGTTATTTTCCACATTAGAAGGGGCAGTTTTAAAAATGGCTATCAGGAAAGGGCATTATTGCAAAAGGTTATTATAAAATGGCACTTTTGCAAATCTCCCTTTTGATTGTTATATCTAAGATTTCACCTGTAGAATATCATCCCCTATTAATATCGACCCAAATCATTCTGACCACATAACCCCGTCCTATGAAATTAAACATCATTTTGGTCTTAGGTTTTATATAAACTTCAAATATTTATAATAAAAGAACTAACGAATGTTACTGTAAATGATAACCAAAACATAGAACAAATAGTAAGGAAATTTAAAACGTGTTCACCTAACCCGCGAAGACTCCCAACTCCGTCGGAAAATTTGCTACAAAATAAGTTATCGTAGCAAGTGCGTCGCAATTTGCGGGGGAACTCAGTTTCCTCGCAATTGTCTCGCAAATTTACGAGGGAAGTATTCGTCGCAAAGTTCGCGACAGAATAGCGAGTTGGTCCTGTTCCC

N4=4

TCGGAGAGATCAATTCGTTGGCGAAGAAGAGGTTTG

TCGGAGAGATCTGTTCATCAGCGAACTTGAGGTTTA

TTGGAGACAACAATTCATCGGCAAATTTGAGTTTTA

TTGGAGAGAACAATTCGTCGGCGAAAACAAAGAGGTTTG

TC

AAAATGAAAGTGAATCTCTAATTATTCAGTAAGCGTAAGACCGTTAGTTTCCACATTAGAAGGGGCAGTTTTAAAAATGGCCATTTGGAAAAGGCATTGTTGCAAAAGGTTATTAGAAATTGGCACTTTTGCAAATCTCCCTTTTGATAGTTATGTCTAAAATTTCACCTGTAGAATATCATCCCCTATTAATATCGACCCAAATCATTCTGACTATATAACCCCATCCTATGAAATTAAACATCATTTTGTCTTAGTTTTATATAAACTTCAAATATTTATAATAAGAGAACTAACGAATGTTACCGTAAATAATAACCAAAACATAGAACAAATAGTAAGGAAATTTAAATTGTGTTCACCTAACTCGCGAAGACTCGCAACTCCATCGGAAAATTTGCTAGAGAATAAGTTATCGTCGCAAGTGCGTCGCAATTTGCGGGGGAACTCTGTTTCCCGCAAAGCCCTCGCAAATTTGCGATGTTTTTGCGGGAATCATGACTTCCACGCAGTTTCCTCGCAATTGTCTTGCAAATTTGCGAAGGAAATATACGTCGCAAAGTTTGCGACGGAATAGCGAGTTGGTCATGTTCCC

N5=4

TCGGAGAGATCTATTTGTTGGCGAAGAAGAGGTTTG

TCAGAGAGATCTATTCATCGGCGAATTTGAGGTTTG

TCGGAGAGAACAATTCGTCGGCGAATTTGAGTTTTA

TCAGAGAGAACAATTCGTCGACGAATTTGAGTAGGTTTG

TC

AAAATGAGAGTGAAACCCTAATTATTCAGTAAGGGTAAAACCATTATTTTCCACACTAGAAAGGGCAGTTTTAAAAATGGCCACAGAAAAGGGCATTGTTGCAAAAGGTTATTACAAAAATGGCGACTTTTGCAAATCTCCCTTTTGATTGTTATATCTAAGATTTCACCTGTACAATATCATCCCCTATTAATATCGACCCAAATCATTCTGACCATATATCCCCTGTCCTATAAAATTAAACATTATTTTCTCTTAGTTTTATATAAACTCAATTATTTATAATAATTAAATTAACGAATGTTACTGTAAATGATAACCAAAACATTAAACAAATAGTAAGGAAATTTAAAACGTGTTCACCTAACCTGCGAATACTCGCAACTCCATCGGAAAATTTGCTACAAAATAAGTTATCGTCGCAAGTCGCGTCGCAATTTGCAGGGGGTCTCAGTTTCCTCGCAATTGTCTTTCAAATTTGAGAGGAAAATATTCGTCGCAAAGTTTCGCGACGGAATAGCGAGTTGGTCCTGTTCCC

N6=5

TCGGAAAGATCTATTCGTTGGCGAAGAAGAGGTTTG

TCGGAGAGATCTATTCGTCGGCGAAGAAGAGGTTTG

TCGGATAGATCTATTCGTCGGCGAATTTTAGGTTTG

TCGCAGAGAACAATTCGTCGGCGAATTTGAGTTTTA

TTGGAGAGAACAATTCGTCGGCGAAAACAAAGAGGTTTG

TC

AAAATGAAAGTGAAACCCTAATTATTCAGTAGGGGTAAGACCGTTATTTTCCATATTCGAAGGGACAGTTTTAGAAATGGCCATCAGAAAAGGGCATTGTTGCAAAAGGTTATTACAAAAATGGCATTTTTGCAAATCTCCCTTTTGATTGTTATATCTATGATTTCACCTGTAGAATATCATCCCGTATTAATATCGATCCAAATCATTCTGACCATATAACCCCCGTCCTATGAAATTAAACATTATTTTGTCCTAGTTTTATATAAACTTCAAATATTTATAATAAGAGAACCAACGAATGTTACTGTAAATGATAACCAAAACATAGAACAAATAGTAAGGAAATTTAAAACGTGTTCACTTAACCGGCGAAGACTCGCAACTCCATCGAAAAATTTGCCACAAAAGTATGTTATCGTCGCAAGTGCGTTGCAATTTGCAGGGGAACTCAGTTTCCCGCAAACCCTCGCAAATTTGCGACGTTTTTGCAGGGAATCATGACTTCCACGTAGTTTACTCGCAATTGTCTCGCAAATTTGCGAGAGAAATATTCGTCGCAAATT

N7=6

TGCGAGGGAAATATTCGTCGGCGAAGAAGAGGTTTG

TCGGAGAGATCTATTCGTCGGCGAAGAAGAGGTTTG

TCGGAGAGATCTATTCGTCGGCGAAGAAGAGGTTTG

TCGGAGAGAACAATTCGTCGGCGAATTTGAGTTTTA

TCGGAGAGAATAATTCGTCTGAAAATTTTAGTTTTA

TCGGAGAGAACAATTCGTTGGCGAATTTGATTAGATTTG

TC

AAAATGAAAGTGAAACCCTAATTATTCAGTAAGGGTAAGACCGTTATTTTCGACATTAGAAGGGGCAGTTTTAAAAAAGGTCATAAGGAAAGGGCATTGTTGCAAAAGGTTATTATAAAAAGGGCACTTATACAAATCTTCCTTTTGATTGTTATATCTAAGATTTCATATGTAGAATATCATCCCGTATTAATATAGACCCAAATCATTCTGACCATATAACCCTGTCCTATGAAATTAAACATCATTTTGTCTTGGTTTTATAAACTTCAAATATTTATAATAAGACTCGCAATCATAGACCCTAACCCGCGAAGACTCGCAACTCCGTCGCAAAAATTGGTACAGAATAAGTTATCGTCGCAAGTGCGTCGCAATTTGCGGGGGAACTCAGTTTCCCGCAAAACCCTCACAAATTTGCGACGCTTTCGCGGGGAATCATGACTTCCACGCAATTTCCTCGCAATTGTGTAGCAAACGTGCGAGGGAAATATTCGTCGCAATATTTGCGACGGAATAGCGAGTTGGTCATGTTCCC

N8=5

TCCGAGAGATCTATTCGTCGGCAAAGAAGAGGTTTG

TCGGAGAGATCTATTCGTCGGCGAAGAAGAGGTTTG

TCGGAGAGATCTATTCGTCGGCAAATTTGAGGTTTG

CGGAGTGAACAATTCTTCGTCGAATTTGAGTTTTA

TCAAAGAGAATAATTCGTCGGCAAATTTGAGTAAGTTTG

TC

AAAATGAAAGTGAAACCCTAATTATTCAGTAAGGGTAAGACCGTTATTTTCCACATTAGAAGGGGCAGTTTTAGAAATGGCCATCAGAAAATGGCATTGATTGCAAAAGGTTATTAGAAAAGGGCACTTTTGCAAATCTCCCTTTTGATTGTTATATATCTAAGATTTTACCTGTAGAATATCATCCCCTATTAATATCGACCCAAATCATTCTGACCATATAACCCTGTCCTATAAAATTAAACATCATTTTGTCTTAGTTTTATATAAACTTCAAATATTTATAATAAGAGAACTAATGAATGTTACTGTAAATGATAACCAAAACATAGAAAAAATAGTAAGGAAATTTAAAACGTGTTCACCTAACCCGCGAAGACTCGCAACTCCATCGGAAAATTTGCTACAGAATAAGTTATCGTTCGCAAGTGCGTCGCAATTTACGGGGAAACTCAGTTTCCCGCAAAGCCCTCGCAAATTTGCAAAGTTTTTGCGGGGAATCATTGTCTCGCAAATCTGCGAGGGAAATATTCGTCTCAAAGTTTGCGATGGAATAGCGAGTTAGTCATGTTCCA

N9=5

TCGGAGAGATCTATTCGTCGGCGAAGAAGAGATTTG

TCAGAGAGATCTATTCGTCGGTGAAGAAGAGGTTTG

TCGAAGAGATCTATTTGTCGGCGAATTTGAGGTTTG

TCGGAGAAATAATTCATCGGCGAATTTGAGTTTTA

TCGGACAGAACAATTCGTCGGCGAATTTGAGTAGGTTTG

TC

AAAATAAAAGTGAAACCCTAATTATTCAGTAAAAGGTAAGACCGTTATTTTCCACGTTCGAAGGGGCAACTTTTCAAAAGGCTATCAGGAAAGGGCATTGTTGCAAAAGATTATTAGAAAAATGGCACTTTTGCAAATCTCCCTTTTGATGTTTGTATCTTAGATTTCACCTCTAGAATATCATCTCGTATTAATATCGACACAAATCATTCTAACCATATAACCCCGTCCTATGAAATTAAACATCATTTTGTCTTAGTTTTATAAACTTGAAATATTTATAATAACACTCGCATCCTAGACCCTAACCCGCGAAGACTCGCGAACTCCGTCGCAAAAATTGCTACACAATAAGTTATCGTCGCAAGTGCTTCGCAATTTGCGGGGGGAACTCAGTTTCCCGCAAAGCCCTCATAAATTTGCTACGTT

N10=4

-------------TTTGTCGGCGAATTTGAGGTTTG

TCGGAGAGAACAATTCGTCGGCGAATTTGAGGTTTG

TCGGAGAGAACAATTCGTCGGCGAATTTGAGTTTTA

TTAGAGAAAAAGATTCGCCGGCGAAAACGAAGAGGTTTG

TC

AAAATGAAAGGAAAACCCTAATTATTCAGTAAGGGTAAGACCGTTATTTTCCTTATTAGAAGGGGCAATTCTAAAAATGGCCATTAGGAAAGGGCATTGTTGCAAAAGATTATTAGAAATAGGGCACTTTTGCAAATCTCCCTTTTGATTGTTATATCTAAGATTTCACCTGTAGAATATCATCACGTATTAATATCGACCCAAATCATTCTGACTATATAACCGCGTCTTATGAAATTAAATATCATTTTGTCTTAGTTAAAAAACTTCAAATATTTATAATAA

GACTCGCAACCCTAAACCCTAACCCGCGAAACTCGCAACTCCGTCGCAAAAATTGCTACAGAATAAGTTTTCGTCGCAAGTGCGTCGCAATTTGCGTGGGAACTCAGTTTCACGCAAATCCTTTGCAAATTTGCGACGTTTTTGCGGGGAATCAGTGACTTCCACGCAGTTTCCATGCAATTGTCTTGCAAATTTGCGAGGGAAATATTCGTCGCAATGTTTGCGACGGAATAGCGAGTTGGTCCTGTTCCCTCGCAAACCTCTCGCAACTCTGTAACAATTTGCAAGGGATGTTTTCCATCGCAAATTTTTCTCGCAAATCGCATGTTTTCTTGTTGTCGAAAAAAAAAAAAATCTAAACTATTTGCTTCATCATCGCTCTTCCTACAAACCAAACTGGCTTGAAAAAAGCTGTCATTGTTTTCATCTTGTGTTATCCATCATTATCTGTCCATCTATATGTTTATATTTTGATAAAACTTGTATCTTTTGATATCATTTACATTGATTTCACTTGTTGGACACATAGAAGTGTAGAGAAATAATTTGCATTCCTTGATTTAGTGGACATAATACTGTGGAACTCAATTTGCGAGGAACTCAGTTTCCCGCAAAGCTCTCGCAACTCTGTAGCAATTTGCGAGGGATGTTTTCCATCGCAAATTTCCCTCGCAAATCGTCTGTTTTCTTGTAGCGAAAAAGAAAAAATCTAAACTATTTGCTTCATCATCGCTCTTCCTACAAAACAACCTGGCTTGAAAAAAGGTGTCATTGTTTTCATAATGTGTTATTTTATCATTATCTGTCCATCTATATGTTTATATTTCGCTAAAACTTGTATCTTTTGATATCATTTACATGGACTTTCACTTGTTGGACACATAGAAGTGTAGAGAAATAATTTGCATTCCTTGATTTAGTGGACATGATATTGTGGATACTATCTAACTTGCAGGAAACTATTTCTTATATTTTTTCAGATCAGTTTCTTGTTTAAAGGGGATCAAGTCAAACCACGGCATGGACTCTTCTACGGTCAATACATTCTCTCTCTGGAACATTAAAGACATAAACACCATTTAGATTATGAAGATTATTTTTTTACGCTACAAGTGACAACAAAATTGTTAGCAAATAAAGAAGTAATGACCCCCAAAAAAAGAAAGAAAACAATACAACACGTTTCATCGAGAAACTTACAGTGTTAAAGAAACATATAGTTATTTCATCATTTGACTGATTGATAATATGAGTTTTGAGTGACTGAGCAATGCAACTAACATCAATGTGAAAATGACTTTTTGTTTGTCTTCTTTCTCTTGTCAAAAATCAATATAGTACAAAAGGGCAAGTAGAGCTGAACATTTTGGGAGAAGCATCAATTAGATAGACTACAAATTCTTTGGAATTCTATGGAAGCCTCTTTTTCCTGTTGCACCAAAGATTCATCACTAACCAATACTAATCAATGTTTCATTTCTTTTTTGGCTTTCACTAAAAAGTCCAAGAAGAAAAAAAGGTCAAAACTGAAATATATTATTTACATCAAACATAATCAATATAACCGGTTAAGCAATTAATGATACAGAAGACTCATCAATGGACAACTATGATATCATTTACATGAATTTGACTTGTTGGACACACAGAAGTGGACAACAATAGTTTGCAATCCTTGATTTAGTGGACATGATATTGTGGATACTATGTAATTTGCATAAACTATTTTTTTTATTTGGGTGGATATAAGGACAACTATGATATCATTTACATGGATTTGACTTGTTGGACACATAGAAGTGGACAACAATAGTTTGCAGTCCTTGATTTAGTGGACATGATATTGTGGATACTATCTAATTTGCATAAACTATTTCTTCTATTTTTTTAGATCAGTTTCTTGTTTAATGGGGATCAAGTCAAACCACAGCATGGACTCTACTATTGTTGATAAAAAATTTTATTTTTAGAAAGCAACATGACTTTTCCTTTCTCAATTCTAGCCCATTATGATTTATGTACATAGATATCTTATATTTTTTCTGCTAACGATGCAGAATGCAGTTGAAATGGCAACATTCATCTGATTCATGGTAAGGCCACAGTCTAAATTATGTGCATCAATCCTTAAATCAGGTATGTAATGTTATTCAGAAAAAAACTTTCATTTAATATTTCGGTGGGGAATAAAAGAAATTTTTTGCTTTATGAAGAACCATACGATGATATAAAGCCGACTAAAGTCTGAGTGCGTTGGAAGTACACAATAATTTGCAGCAACACACTATCTCTAATCGTCACTCTCGTTAGTCTAAACTTTTCAGATATCTACAAGTTCCATAATCTTAAGCTTATCAAAGTCTATGATAAAACATCGCTCTGCCAGGTCAATAC

(c) Alignment high-speed rail model sequences of Ler-0_seq and NCBI.

NCBI -TTTGCGACGGAATAGCGAGTTGGTCCTGTTCTCTCGGAGAGATCTATTCGTCGGCGAAG

Ler-0 TTTTGCGACGGAATAGCGAGTTGGTCCTGTTCTCTCGGAGAGATCTATTCGTCGGCGAAG

***********************************************************

NCBI AAGAGGTTTGTGGGAGAGATCTATTCGTCGGCGAAGAAGAGGTTTGTCGGAGAGATCTAT

Ler-0 AAGAGGTTTGTGGGAGAGATCTATTCGTCGGCGAAGAAGAGGTTTGTCGGAGAGATCTAT

************************************************************

NCBI TCGTCGGCGAATTTGAGGTTTGTCGGAGAGAACAATTCATCGGCGAATTTGAGTTTTATC

Ler-0 TCGTCGGCGAATTTGAGGTTTGTCGGAGAGAACAATTCATCGGCGAATTTGAGTTTTATC

************************************************************

NCBI GGAGAGAACAATTCGTCGGCGAAAACAAAGAGGTTTGTCAAAATGAAACTGAAACCTTAA

Ler-0 GGAGAGAACAATTCGTCGGCGAAAACAAAGAGGTTTGTCAAAATGAAACTGAAACCTTAA

************************************************************

NCBI TTTTTCAGTAAGGGTAACACCGTTATTTACCACATTAGAAGGGGCAGTTTTAAAAATGAC

Ler-0 TTTTTCAGTAAGGGTAACACCGTTATTTACCACATTAGAAGGGGCAGTTTTAAAAATGAC

************************************************************

NCBI CATCAGGAAAGGGCATTGTTGCAAAAGGTTATTAGAAAAGGCACTTTTGCAAATCTCCCT

Ler-0 CATCAGGAAAGGGCATTGTTGCAAAAGGTTATTAGAAAAGGCACTTTTGCAAATCTCCCT

************************************************************

NCBI TTTGATTGTTATATCTAAGATTTCACTTGTACAATATCATCCCCTATTAATATCGACCCA

Ler-0 TTTGATTGTTATATCTAAGATTTCACTTGTACAATATCATCCCCTATTAATATCGACCCA

************************************************************

NCBI AATCATTCTAACCATATAACCCCGTCCTATGAAATTAAACATTATTTTGTCTTAGTTTTA

Ler-0 AATCATTCTAACCATATAACCCCGTCCTATGAAATTAAACATTATTTTGTCTTAGTTTTA

************************************************************

NCBI TATAAACTTCAAATATTTATAATAATAAAACTAACGAATGTTACTGTAAATGATAACCAA

Ler-0 TATAAACTTCAAATATTTATAATAATAAAACTAACGAATGTTACTGTAAATGATAACCAA

************************************************************

NCBI AACATTAAACAAATAGTAAGGAAATTTAAAACGTATTAACCTAACCCGAAAAGACTCGCA

Ler-0 AACATTAAACAAATAGTAAGGAAATTTAAAACGTATTAACCTAACCCGAAAAGACTCGCA

************************************************************

NCBI ACTCCGTCGCAAAAATTGCTACAGAATAAGTTATCGTCGTAAGTGCGTCGCAATTTGCGG

Ler-0 ACTCCGTCGCAAAAATTGCTACAGAATAAGTTATCGTCGTAAGTGCGTCGCAATTTGCGG

************************************************************

NCBI GGGAACTCAGTTTCCCGCAAAGCCCTCGCAGACTTGCGACGTTTTGCGGGGAATCATGGC

Ler-0 GGGAACTCAGTTTCCCGCAAAGCCCTCGCAGACTTGCGACGTTTTGCGGGGAATCATGGC

************************************************************

NCBI TTCTACGCAGTTTCCTCGCAATTGTCTCGCAAATTTGCGAGGGATATATTCGTCGTAAAG

Ler-0 TTCTACGCAGTTTCCTCGCAATTGTCTCGCAAATTTGCGAGGGATATATTCGTCGTAAAG

************************************************************

NCBI TTCGCGACGGAATAGCGAGTTGGTCATGTTCCCTCAGAGAGATCTATTTATCGGCGAAGA

Ler-0 TTCGCGACGGAATAGCGAGTTGGTCATGTTCCCTCAGAGAGATCTATTTATCGGCGAAGA

************************************************************

NCBI AGAGGTTTGTCGAAGAGAACAATTCGTCGGCGAATTTGAGTTTTATTGGAGAGAACAATT

Ler-0 AGAGGTTTGTCGAAGAGAACAATTCGTCGGCGAATTTGAGTTTTATTGGAGAGAACAATT

************************************************************

NCBI TGTCGGCGAAAACAAAGAGGTTTGTCAAAATGAAAGTGAAACCCTAATTATTCAGTAAGG

Ler-0 TGTCGGCGAAAACAAAGAGGTTTGTCAAAATGAAAGTGAAACCCTAATTATTCAGTAAGG

************************************************************

NCBI GTAACACCGTTATTTTCCACAATAGAAGGGGCAGTTTTAAAAATGGCTATTAGGAAAGGG

Ler-0 GTAACACCGTTATTTTCCACAATAGAAGGGGCAGTTTTAAAAATGGCTATTAGGAAAGGG

************************************************************

NCBI CATTGTTGCAAAAGGTTATTAGAAAAGGGAACTTTCGCAAATGTCCCTTTTGATGGTTAT

Ler-0 CATTGTTGCAAAAGGTTATTAGAAAAGGGAACTTTCGCAAATGTCCCTTTTGATGGTTAT

************************************************************

NCBI ATCTAAGATTTCACCTGTACAATATCATCTCCTATTAATATCGACCCAAATCATTCTAAC

Ler-0 ATCTAAGATTTCACCTGTACAATATCATCTCCTATTAATATCGACCCAAATCATTCTAAC

************************************************************

NCBI CATATAACCCCGTCCTATGAAATTAAACATCATTTTGTCTTAGTTTTATATAAACTTCAA

Ler-0 CATATAACCCCGTCCTATGAAATTAAACATCATTTTGTCTTAGTTTTATATAAACTTCAA

************************************************************

NCBI ATATTTATAATAAGAGAACTAACGAATGTTACTGTAAATGATAACCAAAACATAGAACAA

Ler ATATTTATAATAAGAGAACTAACGAATGTTACTGTAAATGATAACCAAAACATAGAACAA

************************************************************

NCBI ATAGTAAGGAAATTTAAAACGTATTCACCTAACCCGCGAAAACTCGCAACTCCATCGGAA

Ler-0 ATAGTAAGGAAATTTAAAACGTATTCACCTAACCCGCGAAAACTCGCAACTCCATCGGAA

************************************************************

NCBI AATTTGCTAAAGAATAAGTTATCGTCGCAAGTGCGTCGCAATTTGCGGGGGAACTCAGTT

Ler-0 AATTTGCTAAAGAATAAGTTATCGTCGCAAGTGCGTCGCAATTTGCGGGGGAACTCAGTT

************************************************************

NCBI TCCACGCAATTGTCCCGCAAATTTGCGAGGGCAATATTCGTCACAAAGTTTGTGACGGAA

Ler-0 TCCACGCAATTGTCCCACAAATTTGCGAGGGCAATATTCGTCACAAAGTTTGTGACGGAA

**************** *******************************************

NCBI TAGCGAGTTGGTCCTGTTCCCTCGGAGAGATCTATTCGTCGGCGAAGAAGAGATTTGTCA

Ler-0 TAGCGAGTTGGTCCTGTTCCCTCGGAGAGATCTATTCGTCGGCGAAGAAGAGATTTGTCA

************************************************************

NCBI GAGAGATCTATTCGTCGGTGAAGAAGAGGTTTGTCGAAGAGATCTATTCGTCGGCGAATT

Ler-0 GAGAGATCTATTCGTCGGTGAAGAAGAGGTTTGTCGAAGAGATCTATTCGTCGGCGAATT

************************************************************

NCBI TGAGGTTTGTCGGAGAGAACAATTCGTCGGCGAATTTGAGTTTTATCGGACAGAACAATT

Ler-0 TGAGGTTTGTCGGAGAGAACAATTCGTCGGCGAATTTGAGTTTTATCGGACAGAACAATT

************************************************************

NCBI CGTCGGCGAAAACGGAGAGGTTTGTCAAAATAAAAGTGAAACCCTAATTATTCAGTAAGG

Ler-0 CGTCGGCGAAAACGGAGAGGTTTGTCAAAATAAAAGTGAAACCCTAATTATTCAGTAAGG

************************************************************

NCBI GTAAGACCGTTATTTTCCACATTAGAAGGGGCAGTTTTAAAATGGCTATCAGGAAAGGGC

Ler-0 GTAAGACCGTTATTTTCCACATTAGAAGGGGCAGTTTTAAAATGGCTATCAGGAAAGGGC

************************************************************

NCBI ATTATTGCAAAAGGTTATTAGAAAATGGCAGTTTTGCAAATCTCCCTTTTGATTGTTATA

Ler-0 ATTATTGCAAAAGGTTATTAGAAAATGGCAGTTTTGCAAATCTCCCTTTTGATTGTTATA

************************************************************

NCBI TCTAAGATTTCACCTGTAGAATATCATCCCCTATTAATATCAACCCAAATCATTCTGACC

Ler-0 TCTAAGATTTCACCTGTAGAATATCATCCCCTATTAATATCAACCCAAATCATTCTGACC

************************************************************

NCBI ACATAACCCCGTCCTATGAAATTAAACATCATTTTGTCTTAGTTTTATATAAACTTCAAA

Ler-0 ACATAACCCCGTCCTATGAAATTAAACATCATTTTGTCTTAGTTTTATATAAACTTCAAA

************************************************************

NCBI TATTTATAATAAAAGAACTAACGAATGTTACTGTAAATGATAACCAAAACATAGAACAAA

Ler-0 TATTTATAATAAAAGAACTAACGAATGTTACTGTAAATGATGACCAAAACATAGAACAAA

***************************************** ******************

NCBI TAGTAAGGAAATTTAAAACGTGTTCACCTAACCCGCGAAGACTCGCAACTCCGTCGGAAA

Ler-0 TAGTAAGGAAATTTAAAACGTGTTCGCCTAACCCGCGAAGACTCGCAACTCCGTCGGAAA

************************* **********************************

NCBI ATTTGCTACAAAATAAGTTATCGTCGCAAGTGCGTCGCAATTTGCGGGGGAACTCAGTTT

Ler-0 ATTTGCTACAAAATAAGTTATCGTCGCAAGTGCGTCGCAATTTGCGGGGGAACTCAGTTT

************************************************************

NCBI CCTCGCAATTGTCTCGCAAATTTACGAGGGAAATATTCGTCGCAAAGTTCATGACGGAAT

Ler-0 CCTCGCAATTGTCTCGCAAATTTACGAGGGAAATATTCGTCGCAAAGTTCATGACGGAAT

************************************************************

NCBI AGCGAGTTGGTCCTGTTCCCTCGGAGAGATCAATTCGTCGGCGAAGAAGTGGTTTGTCGG

Ler-0 AGCGAGTTGGTCCTGTTCCCTCGGAGAGATCAATTCGTCGGCGAAGAAGTGGTTTGTCGG

************************************************************

NCBI GGAGATCTGTTCGTCAGCGAATTTGAGGTTTGTCGGAGACAACAATTCATCGGCGAATTT

Ler-0 GGAGATCTGTTCGTCAGCGAATTTGAGATTTGTCGGAGACAACGATTCATCGGCGAATTT

*************************** *************** ****************

NCBI GAGTTTTATTGGAGAGAACAATTCGTCGGCGAAAACAAAGAGGTTTGTCAAAATGAAAGT

Ler-0 GAGTTTTATTGGAGAGAACAATTCGTCGGCGAAAACAAAGAGGTTTGTCAAAATGAAAGT

************************************************************

NCBI GAATCTCTAATTATTCAGTAAGGGTAAGACCGTTATTTTCCACATTAGAAGAGGCAGTTT

Ler GAATCTCTAATTATTCAGTAAGGGTAAGACCGTTATTTTCCACATTAGAAGAGGCAGTTT

************************************************************

NCBI TAAAAATGGCCATTTGGAAAAGGCATTGTTGCAAAAGGTTATTAGAAATTGGCACTTTTG

Ler-0 T-AAAATGGCCATTTGGAAAAGGCATTGTTGCAAAAGGTTATTAGAAATTGGCACTTTTG

* **********************************************************

NCBI CAAATCTCCCTTTTGATTGTTATATCTAAGATTTCACCTGTACAATATCATCCCCTATTA

Ler-0 CAAATCTCCCTTCTGATTGTTATATCTAAGATTTCACCTGTACAATATCATCCCCTATTA

************ ***********************************************

NCBI ATATCGACCCAAATCATTCTGACCATATAACCCCGTCCTATGAAATTAAACATCATTTTG

Ler-0 ATATCGACCCAAATCATTCTGACCATATAACCCCGTCCTATGAAATTAAACATCATTTTG

************************************************************

NCBI TCTTAGTTTTATATAAACTTCAAATATTTATAATAAGAGAACTAACGAATGTTACCGTAA

Ler-0 TCTTAGTTTTATATAAACTTCAAATATTTATAATAAGAGAACTAACGAATGTTACCGTAA

************************************************************

NCBI ATGATAACCAAAACATAGAACAAATAGTAAGGAAATTTAAATTGTGTTCACCTAACTCGC

Ler-0 ATGATAACCAAAACATAGAACAAATAGTAAGGAAATTTAAATTGTGTTCACCTAACTCGC

************************************************************

NCBI GAAGACTCGCAACTTCATCGGAAAATTTGCTAGAGAATAAGTTATCGTCGCAAGTGCGTC

Ler-0 GAAGACTCGCAACTTCATCGGAAAATTTGCTAGAGAATAAGTTATCGTCGCAAGTGCGTC

************************************************************

NCBI GCAATTTGC-GGGGGAACTCTGTTTCCCGCTAAGCCCTCGCAAATTTGCGATG-TTTTTG

Ler-0 GCAATTTGCGGGGGGAACTCTGTTTCCCGCTAAGCCCTCGCAAATTTGCGATGTTTTTTG

********* ******************************************* ******

NCBI CGGGAATCATGACTTCCACGCAGTTTCCTCGCAATTGTCTTGCAAATTTGCGAAGGAAAT

Ler-0 CGGGAATCATGACTTCCACGCAGTTTCCTCGCAATTGTCTTGCAAATTTGCGAAGGAAAT

************************************************************

NCBI ATACGTCGTAAAGTTTGCGACGGAATAGCGAGTTGGTCATGTTCCCTCGGAGAGATCTAT

Ler-0 ATACGTCGTAAAGTTTGCGACGGAATAGCGAGTTGGTCATGTTCCCTCGGAGAGATCTAT

************************************************************

NCBI TCGTCGGCGAAGAAGAGGTTTGTCGGAGAGATCTATTCGTCGGCGAATTTGAGATTTGTC

Ler-0 TCGTCGGCGAAGAAGAGGTTTGTCGGAGAGATCTATTCGTCGGCGAATTTGAGATTTGTC

************************************************************

NCBI GGAGAGAACAATTCGTCGGCGAATTTGAGTTTTATCAGAGAGAACAATTCGTCGGCGAAT

Ler-0 GGAGAGAACAATTCGTCGGCGAATTTGAGTTTTATCAGAGAGAACAATTCGTCGGCGAAT

************************************************************

NCBI TTGAGTTGGTTTGTCAAAATGAGAGTGAAACCCTAATTATTCAGTAAGGGTAAAACCATT

Ler-0 TTGAGTTGGTTTGTCAAAATGAGAGTGAAACCCTAATTATTCAGTAAGGGTAAAACCATT

************************************************************

NCBI ATTTTCCACATTAGAAAGGGCAGTTTTAAAAATGGCCATCAGAAAAGGGCATTGTTGTAA

Ler-0 ATTTTCCACATTAGAAAGGGCAGTTTTAAAAATGGCCATCAGAAAAGGGCATTGTTGTAA

************************************************************

NCBI AAGGTTATTACAAAAATGGCACTTTTGGAAATCTCCCTTTTGATTGTTATATCTAAGATT

Ler-0 AAGGTTATTACAAAAATGGCACTTTTGGAAATCTCCCTTTTGATTGTTATATCTAAGATT

************************************************************

NCBI TCACCTGTACAATATCATCCCCTATTAATATCGACCCAAATCATTCTGACCATATAACCC

Ler-0 TCACCTGTACAATATCATCCCCTATTAATATCGACCCAAATCATTCTGACCATATAACCC

************************************************************

NCBI CGTCCTATGAAATTAAACATTATTTTGTCTTACTTTTATATAATTTCAAATATTTATAAT

Ler-0 CGTCCTATGAAATTAAACATTATTTTGTCTTACTTTTATATAATTTCAAATATTTATAAT

************************************************************

NCBI ATTTAAACTAACGAATGTTACTGTAAATGATAACCAAAACATTAAACAAATAGTAAGGAA

Ler-0 ATTTAAACTAACGAATGTTACTGTAAATGATAACCAAAACATTAAACAAATAGTAAGGAA

************************************************************

NCBI ATTTAAAACGTGTTCACCTAACCTGCGAATACTCGCAACTCCATCGGAAAATTTGCTACA

Ler-0 ATTTAAAACGTGTTCACCTAACCTGCGAATACTCGCAACTCCATCGGAAAATTTGCTACA

************************************************************

NCBI AAATAAGTTATCGTCGCAAGGGCGTCGCAATTTGCAGGGGGTCTCAGTTTCCTCGCAATT

Ler-0 AAATAAGTTATCGTCGCAAGGGCGTCGCAATTTGCAGGGGGTCTCAGTTTCCTCGCAATT

************************************************************

NCBI GTCTTTCAAATTTGCGAGGGAAATATTCGTCGCAAAGTTTACGACGGAATAGCGAGTTGG

Ler-0 GTCTTTCAAATTTGCGAGGGAAATATTCGTCGCAAAGTTTACGACGGAATAGCGAGTTGG

************************************************************

NCBI TCCTGTTCCCTCGGAGAGATCTATTCGTTGGCGAAGAAGATGTTTGTCGGAGAGATCTAT

Ler-0 TCCTGTTCCCTCGGAGAGATCTATTCGTTGGCGAAGAAGATGTTTGTCGGAGAGATCTAT

************************************************************

NCBI TCGTCGGCGAAGAAGAGGTTTGTCGGATAGATCTATTCGTCGGCGAATTTGAGGTTTGTC

Ler-0 TCGTCGGCGAAGAAGAGGTTTGTCAGATAGATCTATTCGTCGGCGAATTTGAGGTTTGTC

************************ ***********************************

NCBI GCAGAGAACAATTCGTCGGCGAATTTGAGTTTTATTGGAGAGAACAATTCGTCGGCGAAA

Ler-0 GCAGAGAACAATTCGTCGGCGAATTTGAGTTTTATTGGAGAGAACAATTCGTCGGCGAAA

************************************************************

NCBI TCAAAGAGGTTTGTCAAAATGAAAGTGAAACCCTAATTATTCAGTAGGGGTAAGACCGTT

Ler-0 TCAAAGAGGTTTGTCAAAATGAAAGTGAAACCCTAATTATTCAGTAGGGGTAAGACCGTT

************************************************************

NCBI ATTTTCCATATTCGAAGGGACAGTTTTAGAAAGGGCCATCAGGAAAGGGCATTGTTGCAA

Ler-0 ATTTTCCATATTCGAAGGGACAGTTTTAGAAAGGGCCATCAGGAAAGGGCATTGTTGCAA

************************************************************

NCBI AAGGTTATTACAAAAATGGCATTTTTGCAAATCTCCCTTTTGATTGTTATATCTATGATT

Ler-0 AAGGTTATTACAAAAATGGCATTTTTGCAAATCTCCCTTTTGATTGTTATATCTATGATT

************************************************************

NCBI TCACCTGTAGAATATCATCCCGTATTAATATCGATCCAAATCATTCTGACCATATAACCC

Ler-0 ACACCTGTAGAATATCATCCCGTATTAATATCGATCCAAATCATTCTGACCATATAACCC

***********************************************************

NCBI CGTCCTATGAAATTAAACATTATTTTGTCTTAGTTTTATATAAACTTCAAATATTTATAA

Ler-0 CGTCCTATGAAATTAAACATTATTTTGTCTTAGTTTTATATAAACTTCAAATATTTATAA

************************************************************

NCBI TAAGAGAACCAACGAATGTTACTGTAAATGATAACCAAAACATAGAACAAATAGTTTGGA

Ler-0 TAAGAGAACCAACGAATGTTACTGTAAATGGTAACCAAAACATAGAACAAATAGTTTGGA

****************************** *****************************

NCBI AATTTAAAACGTGTTCACCTAACCGGCGAAGACTCGCAACTCCATCAAAAAATTTGCCAC

Ler-0 AATTTAAAACGTGTTCACCTAACCGGCGAAGACTCGCAACTCCATCAAAAAATTTGCCAC

************************************************************

NCBI AAAATAAGTTATCGTCGCAAGTGCGTTGCAATTTGCAGGGGAACTCAGTTTCCCGCAAAC

Ler-0 AAAATAAGTTATCGTCGCAAGTGCGTTGCAATTTGCAGGGGAACTCAGTTTCCCGCAAAC

************************************************************

NCBI CCTCGCAAATTTGCGACGTTTTTGCAGGGAATCATGACTTCCACGTAGTTTACTCGCAAT

Ler-0 CCTCGCAAATTTGCGACGTTTTTGCAGGGAATCATGACTTCCACGTAGTTTACTCGCAAT

************************************************************

NCBI TGTCTTGCAAAAGGTTATGTTCCCTCGGAGAGATCTATTTTTCGGCGAAGAAGAGGTTTG

Ler-0 TGTCTTGCAAAAGGTTATGTTCCCTCGGAGAGATCTATTTTTCGGCGAAGAAGAGGTTTG

************************************************************

NCBI TCGGAGAGATCTATTCGTCGGCGAAGAAGAGGTTTGTCGGAGAGATCTATTCGTCGGCGA

Ler-0 TCGGAGAGATCTATTCGTCGGCGAAGAAGAGGTTTGTCGGAGAGATCTATTCGTCGGCGA

************************************************************

NCBI AGAAGAGGTTTGTCGGAGAGAACAATTCGTCGGCGAATTTGAGTTTTATCGGAGAAAATA

Ler-0 AGAAGAGGTTTGTCGGAGAGAACAATTCGTCGGCGAATTTGAGTTTTATCGGAGAAAATA

************************************************************

NCBI ATTCGTCTGAGAATTTGAGTTTTATCGGAGAGAACAATTCGTTGGCGAATTTAATTAGAT

Ler-0 ATTCGTCTGAGAATTTGAGTTTTATCGGAGAGAACAATTCGTTGGCGAATTTAATTAGAT

************************************************************

NCBI TTGTCAAAATGAAAGTGAAATCCTAATTATTCAGTAAGGGTAAGACCGTTATTTTCGACA

Ler-0 TTGTCAAAATGAAAGTGAAATCCTAATTATTCAGTAAGGGTAAGACCGTTATTTTCGACA

************************************************************

NCBI TTAGAA-GGGGCAGTTTTAAAAAAGGTCATAAGGAAAGGGCATTGTTGCAAAAGGTTATT

Ler-0 TTAGAAGGGGGCAGTTTTAAAAAAGGTCATAAGGAAAGGGCATTGTTGCAAAAGGTTATT

****** *****************************************************

NCBI ATAAAAAGGGCACTTATACAAATCTTCATTTTGATTGTTATATCTAAGATTTCACATGTA

Ler-0 ATAAAAAGGGCACTTATACAAATCTTCATTTTGATTGTTATATCTAAGATTTCACATGTA

************************************************************

NCBI GAATATCATCCCGTATTAATATCGACCCAAATCATTCTGACCATATAACCCTGTCCTATG

Ler-0 GAATATCATCCCGTATTAATATCGACCCAAATCATTCTGACCATATAACCCTGTCCTATG

************************************************************

NCBI AAATTAAACATCATTTTGTCTTAGTTTTATAAACTTCAAATATTTATAATAAGACTCGCA

Ler-0 AAATTAAACATCATTTTGTCTTAGTTTTATAAACTTCAAATATTTATAATAAGACTCGCA

************************************************************

NCBI ATCATAGACCCTAACCCGCAAAGACTCGCAACTCCGTCGCAAAAATTGGTACAGAATAAG

Ler-0 ATCATAGACCCTAACCCGCAAAGACTCGCAACTCCGTCGCAAAAATTGGTACAGAATAAG

************************************************************

NCBI TTATCGTCGCAAGTGCGTCGCAATTTGCGGGG--AAACTCAGTTTCCCGCAAAACCCTTG

Ler-0 TTATCGTCGCAAGTGCGTCGCAATTTGCGGGGGAAAACTCAGTTTCCCGCAAAACCCTTG

******************************** **************************

NCBI CAAATTTGCGACGTTTTCGCGGGGAATCATGACTTCCACGCAATTTCCTCGCAATTGTGT

Ler-0 CAAATTTGCGACGTTTTCGCGGGGAATCATGACTTCCACGCAATTTCCCCGCAATTGTGT

************************************************ ***********

NCBI AGCAAACGTGCGAGGGAAATATTCGTCTCAATAATTGCGACGGAATAGCGAGTTGGTCAT

Ler-0 AGCAAACGTGCGAGGGAAATATTCGTCTCAATAATTGCGACGGAATAGCGAGTTGGTCAT

************************************************************

NCBI GTTCCCTCCGAGAGATCTATTCGTCGGCAAAGAAGAGGTTTGTCGGGAAGATCTATTCGT

Ler-0 GTTCCCTCCGAGAGATCTATTCGTCGGCAAAGAAGAGGTTTGTCGGGAAGATCTATTCGT

************************************************************

NCBI CGGCGAAGAAGAGGTTTGTCGGAGAGATCTATTCGTCGGCAAATTTGAGTTTTGCGGAGA

Ler-0 CGGCGAAGAAGAGGTTTGTCGGAGAGATCTATTCGTCGGCAAATTTGAGTTTTGCGGAGA

************************************************************

NCBI GAACAATTCTTCGTCGAATTTGAGTTTTATCAGAGAGTATAATTCGTCGGCGAATTTGAG

Ler-0 GAACAATTCTTCGTCGAATTTGAGTTTTATCAGAGAGTATAATTCGTCGGCGAATTTGAG

************************************************************

NCBI TAGGTTTGTCAAAATGAAAGTGAAACCCTAATTATTCAGTAAGGGTAAGACCGTTATTTT

Ler-0 TAGGTTTGTCAAAATGAAAGTGAAACCCTAATTATTCAGTAAGGGTAAGACCGTTATTTT

************************************************************

NCBI CCACAATAGAA-GGGGCAGTTTTAAAAATGGCCATCAGAAAATGGCATTGTTGCAAAAGG

Ler-0 CCACAATAGAAGGGGGCAGTTTTAAAAATGGCCATCAGAAAATGGCATTGTTGCAAAAGG

*********** ************************************************

NCBI TTATTAGAAAAAGGGCACTTTTGCAAATCTCCCTTTTGATTGTTATATCTAAGATTTTAC

Ler-0 TTATTAGAAAAAGGGCACTTTTGCAAATCTCCCTTTTGATTGTTATATCTAAGATTTTAC

************************************************************

NCBI CTGTTGAATATTATCCCGTATTAATATCAACCCAAATCATTCTGACCATATAACTCCGTC

Ler-0 CTGTTGAATATTATCCCGTATTAATATCAACCCAAATCATTCTGACCATATAACTCCGTC

************************************************************

NCBI CTATAAAATTAAACATCATTTTGTCTTAGTTTTATATAAACTTCAAATATTTATAATAAG

Ler-0 CTATAAAATTAAACATCATTTTGTCTTAGTTTTATATAAACTTCAAATATTTATAATAAG

************************************************************

NCBI AGAACTAACGAGTGTTACTGTAAATGATAACCAAAACATAGAACAAATAGTAAGGAAATT

Ler-0 AGAACTAACGAGTGTTACTGTAAATGATAACCAAAACATAGAACAAATAGTAAGGAAATT

************************************************************

NCBI TAAAACGTGTTCACCTAACCCGCGAAGACTCGCAACTCCATCGGAAAATTTGCTACAGAA

Ler-0 TAAAACGTGTTCACCTAACCCGCGAAGACTCGCAACTCCATCGGAAAATTTGCTACAGAA

************************************************************

NCBI TAAGTTATCGTCGCAAGTGCGTCGCAATTTACGGGGAAACTCAGTTTCCCGCAAAG-CCC

Ler-0 TAAGTTATCGTCGCAAGTGCGTCGCAATTTACGGGGAAACTCAGTTTCCCGCAAAGCCCC

******************************************************** ***

NCBI TCGCAAATTTGCAAAGTTTTTGCGGGGAATCATTGTCTCGCAAATTTGCGAGGGAAATAT

Ler-0 TCGCAAATTTGCAAAGTTTTTGCGGGGAATCATTGTCTCGCAAATTTGCGAGGGAAATAT

************************************************************

NCBI TCGTCTCAAAGTTTGCGATGGAATAGCGAGTTAGTCATGTTCCATCGGAGAGATCTATTC

Ler-0 TCGTCTCAAAGTTTGCGATGGAATAGCGAGTTAGTCATGTTCCGTCGGAGAGATCTATTC

******************************************* ****************

NCBI GTCGGCGAAGAAGAGATTTGTCAGAGAGATCTATTCGTCGGTGAAGAAGAGGTTTGTCGA

Ler-0 GTCGGCGAAGAAGAGATTTGTCAGAGAGATCTATTCGTCGGTGAAGAAGAGGTTTGTCGA

************************************************************

NCBI AGAGATCTATTCGTCGGCGAATTTGAGGTTTGTCGGAGAGAACAATTCGTCGGCGAATTT

Ler-0 AGAGATCTATTCGTCGGCGAATTTGAGGTTTGTCGGAGAGAACAATTCGTCGGCGAACTT

********************************************************* **

NCBI GAGTTTTATTGGACAGAACAATTCGTCGGCGAATTTGAGTAGGTTTGTCAAAATAAAAGT

Ler-0 GAGTTTTATTGGACAGAACAATTCGTCGGCGAATTTGAGTAGGTTTGTCAAAATAAAAGT

************************************************************

NCBI GAAACCCTAATTATTCAGTAAAGGGTAAGACCGTTATTTTCCACGTTCGAAGGGGCAATT

Ler-0 GAAACCCTAATTATTCAGTAAAGGGTAAGACCGTTATTTTCCACGTTCGAAGGGGCAATT

************************************************************

NCBI TTCAAAAGGCTATCAGGAAAGGGCATTGTTGCAAAAGATAATTAGAAAAAGGGCACTTTT

Ler-0 TTCAAAAGGCTATCAGGAAAGGGCATTGTTGCAAAAGATAATTAGAAAAAGGGCACTTTT

************************************************************

NCBI GCAAATCTCCCTTTTGATTGTTATATCTTAGATTTCACCTCTAGAATATCATCTCGTATT

Ler-0 GCAAATCTCCCTTTTGATTGTTATATCTTAGATTTCACCTCTAGAATATCATCTCGTATT

************************************************************

NCBI AATATCGACACAAATCATTCTAACCATATAACCCCGTCCTATGAAATTAAACATCATTTT

Ler-0 AATATCGACACAAACCATTCTAACCATATAACCCCGTCCTATGAAATTAAACATCATTTT

************** *********************************************

NCBI GTCTTAGTTTTATAAACTTCAAATATTTATAATAACACTCGCATCCTAGACCCTAACCCG

Ler-0 GTCTTAGTTTTATAAACTTCAAATATTTATAATAACACTCGCATCCTAGACCCTAACCCG

************************************************************

NCBI CGAAGACTCGCAACTCCGTCGCAAAAATTGCTACAGAATAAGTTATCGTCGCAAGTGCGT

Ler-0 CGAAGACTCGCAACTCCGTCGCAAAAATTGCTACAGAATAAGTTATCGTCGCAAGTGCGT

************************************************************

NCBI CGCAATTTAC-GGGGGAACTCAGTTTTCCGCAAAGTCCTCATAAATTTGCTATGTTTTTG

Ler-0 CGCAATTTACGGGGGGAACTCAGTTTTCCGCAAAGTCCTCATAAATTTGCTATGTTTTTG

********** *************************************************

NCBI CGGGGAATCATGACTTACACACAGTTTCCTCGCAATTGTCTCGCAAATTTGTGAGGGAAA

Ler-0 CGGGGAATCATGACTTACACACAGTTTCCTCGCAATTGTCTCGCAAATTTGTGAGGGAAA

************************************************************

NCBI TATTTGTCTCAAAGTTTACGACGGAATAACGAGTTGGTCATGTTCCCTCAGAGAGATCTA

Ler-0 TATTTGTCTCAATGTTTACGACGGAATAACGAGTTGGTCATGTTCCCTCAGAGAGATCTA

************ ***********************************************

NCBI TTCGTCGGCGAAGAAGAGGTTTGTCGGAGAGATCTATTCGTCGACGAAGAAGAGGTTTGT

Ler-0 TTCGTCGGCGAAGAAGAGGTTTGTCGGAGAGATCTATTCGTCGACGAAGAAGAGGTTTGT

************************************************************

NCBI CGGAGAGATCTATTCGTCGGCGAATTTGAGGTTTGTCGGAGAGAACAATTCGTCGGCAAA

Ler-0 CGGAGAGATCTATTCGTCGGCGAATTTGAGGTTTGTCGGAGAGAACAATTCGTCGGCAAA

************************************************************

NCBI TTTGAGTTTTATCGGAGAGAACAATTCGTCGGCGAATTTGAGTAGGTTTGTCAAAATGAA

Ler-0 TTTGAGTTTTATCGGAGAGAACAATTCGTCGGCGAATTTGAGTAGGTTTGTCAAAATGAA

************************************************************

NCBI AGTGAAACCCTAATTATTTAGTAAAGGGATAGACCGTTATTTTCCACATTAAAAGGGGCA

Ler-0 AGTGAAACCCTAATTATTTAGTAAAGGGATAGACCGTTATTTTCCACATTAAAAGGGGCA

************************************************************

NCBI ATTTTCAAAATGGCTATCAGGAAAGGGCATTGTTGCAAAAGGTTATTAGAAAAAAGGCAC

Ler-0 ATTTTCAAAATGGCTATCAGGAAAGGGCATTGTTGCAAAAGGTTATTAGAAAAAAGGCAC

************************************************************

NCBI TTTTGCAAATCTCCTTTTTGATTGTTATATCTTAGATTTCACTTGTACAATATCATCTCG

Ler-0 TTTTGCAAATCTCCTTTTTGATTGTTATATCTTAGATTTCACTTGTACAATATCATCTCG

************************************************************

NCBI TATTTATATCGACCCAAATCATTCTAACCATATAACCCCGTCCTATGAAATTAAACATCA

Ler-0 TATTTATATCGACCCAAATCATTCTAACCATATAACCCCGTCCTATGAAATTAAACATCA

************************************************************

NCBI TTTTGTCTTAGTTTTCTAAACTTTAAATATTTATAATAATAGAACTAACGAATGTTACAA

Ler-0 TTTTGTCTTAGTTTTCTAAACTTTAAATATTTATAATAATAGAACTAACGAATGTTACAA

************************************************************

NCBI TAAATGATAACCAAAACATAGAACAAATAGTAAGGAAATTTATAACGTGTTCACCTAACC

Ler-0 TAAATGATAACCAAAACACAGAACAAATAGTAAGGAAATTTATAACGTGTTCACCTAACC

****************** *****************************************

NCBI CGCGAAGACTCGCAACTCTGTTGCAAAAATTGCTACAGAATAAGTTATCGTCGCAAGTGC

Ler-0 CGCGAAGACTCGCAACTCTGTTGCAAAAATTGCTACAGAATAAGTTATCGTCGCAAGTGC

************************************************************

NCBI GTTGCAATTTAC-GGGGGAACTCAGTTTCCCGCAAAGCCCTCGCAAATTTGCGACGTTTT

Ler-0 GTTGCAATTTACGGGGGGAACTCAGTTTCCCGCAAAGCCCTCGCAAATTTGCGACGTTTT

************ ***********************************************

NCBI TGCGGGGAATCATGACTTCCATGCAGTTTCCTCGCAATTGTCTCGCAAATTTGCGAGGGA

Ler-0 TGCGGGGAATCATGACTTCCATGCAGTTTCCTCGCAATTGTCTCGCAAATTTGCGAGGGA

************************************************************

NCBI AATATTTGTCGCAAAGTTTGCGACGCAATAACGAGTTGGTCCTGTTCCCTCGGAGAGATC

Ler-0 AATATTTGTCGCAAAGTTTGCGACGCAATAACGAGTTGGTCCTGTTCCCTCGGAGAGATC

************************************************************

NCBI TATTCGTCGGCGAAGAAAAGGTTTGTCGGAGAGATCTATTCGTCGGTGAAGAAGAGGTTT

Ler-0 TATTCGTCGGCGAAGAAAAGGTTTGTCGGAGAGATCTATTCGTCGGTGAAGAAGAGGTTT

************************************************************

NCBI GTTAGAGAGATCTATTCGTCAACGAATTTGAGGTTTGTCGGAGAGAACAATTCGTTGGCG

Ler-0 GTTAGAGAGATCTATTCGTCAACGAATTTGAGGTTTGTCGGAGAGAACAATTCGTTGGCG

************************************************************

NCBI AATTTGAGTTTTATTAGAGAAAAAGATTCGCCGGCGAAAACGAAGAGGTTTGTCAAAATG

Ler-0 AATTTGAGTTTTATTAGAGAAAAAGATTCGCCGGCGAAAACGAAGAGGTTTGTCAAAATG

************************************************************

NCBI AAAGGAAAACCTTAATTATTCAGTAAGGGTAAGACCGTTATTTTCCTTATTAGAAGGGGC

Ler-0 AAAGGAAAACCTTAATTATTCAGTAAGGGTAAGACCGTTACTTTCCTTATTAGAAGGGGC

**************************************** *******************

NCBI AATTTTAAAAATGGCCATTAGGAAAGGGCATTGTTGCAAAAGATTATTAGAAATAGGGCA

Ler-0 AATTTTAAAAATGGCCATTAGGAAAGGGCATTGTTGCAAAAGATTATTAGAAATAGGGCA

************************************************************

NCBI CTTTTGCAAATCTCTCTTTTGATTGTTATATCTAAGATTTCACCTGTAGAATATCATCAC

Ler-0 CTTTTGCAAATCTCTCTTTTGATTGTTATATCTAAGATTTCACCTGTAGAATATCATCAC

************************************************************

NCBI GTATTAATATCGACCCAAATCATTCTGACTATATAACCGCGACTTATGAAATTAAATATC

Ler-0 GTATTAATATCGACCCAAATCATTCTGACTATATAACCGCGACTTATGAAATTAAATATC

************************************************************

NCBI ATTTTGTCTTAGTTTAAAAACTTCAAATATTTATAATAAGGCTCGCAACCCTAAACCCTA

Ler-0 ATTTTGTCTTAGTTTAAAAACTTCAAATATTTATAATAAGGCTCGCAACCCTAAACCCTA

************************************************************

NCBI ACCCGCGAAGACTCGCAACTCCGTCGCAAAAATTGCTACAGAATAAGTTTTCGTCGCAAG

Ler-0 ACCCGCGGAGACTCGCAACTCCGTCGCAAAAATTGCTACAGAATAAGTTTTCGTCGCAAG

******* ****************************************************

NCBI TGCATCGCAATTTGCGTGGGAACTCAGTTTCACGCAAATCCCTTGCAAATTTGCGACGTT

Ler-0 TGCATCGCAATTTGCGTGGGAACTCAGTTTCACGCAAATCCCTTGCAAATTTGCGACGTT

************************************************************

NCBI TTTGCGGGGAATCATGACTTCCACGCAGTTTCCTTGCAATTGTCTTGCAAATTTGCGAGG

Ler-0 TTTGCGGGGAATCATGACTTCCACGCAGTTTCCTTGCAATTGTCTTGCAAATTTGCGAGG

************************************************************

NCBI GAAATATTCGTCGCAATGTTTGCGACGGAATAGCGAGGTGGTCCTGTTCCCCCTAACCCG

Ler-0 GAAATATTCGTCGCAATGTTTGCGACGGAATAGCGAGGTGGTCCTGTTCCC---------

***************************************************

NCBI CGAAGACTCGCAACTCCGTCGCAAAAATTGCTACAGAATAAGTTTTCGTCGTCGCAAACC

Ler-0 ---------------------------------------------------TCGCAAACC

*********

NCBI TCTCGCAACTCTGTAACAATTTGCAAGGGATGTTTTCCATCGCAAATTTCTCTCGCAAAT

Ler-0 TCTCGCAACTCTGTAACAATTTGCAAGGGATGTTTTCCATCGCAAATTTCTCTCACAAAT

****************************************************** *****

NCBI CGCCTGTTTTCTTGTTGTCGAAAAAAAAAAAATCTAAACTATTTGCTTCATCATCGCTCT

Ler-0 CGCCTGTTTTCTTGTTGTCGAAAAAAAAAAAATCTAAACTATTTGCTTCATCATCGCTCT

************************************************************

NCBI TCCTACAAACCAAACTGGCTTGAAAAAAGCTGTCATTGTTTTCATCATGTGTTATCCATC

Ler-0 TCCTACAAACCAAACTGGCTTGAAAAAAGCTGTCATTGTTTTCATCATGTGTTATCCATC

************************************************************

NCBI ATTATCTGTCCATCTATATGTTTATATTTTGATAAAACTTGTATCTTTTGATATCATTTA

Ler-0 ATTATCTGTCCATCTATATGTTTATATTTTGATAAAACTTGTATCTTTTGATATCATTTA

************************************************************

NCBI CATTGATTTCACTTGTTGGACACATAGAAGTGTAGAGAAATAATTTGCATTCCTTGATTT

Ler-0 CATTGATTTCACTTGTTGGACACATAGAAGTGTAGAGAAATAATTTGCATTCCTCGATTT

****************************************************** *****

NCBI AGTGGACATAATATTGTGGAACTCAATTTGCGAGGAACTCAGTTTCCCGCAAAGCTCTCG

Ler-0 AGTGGACATAATATTGTGGAACTCAATTTGCGAGGAACTCAGTTTCCCGCAAAGCTCTCG

************************************************************

NCBI CAACTCTGTAGCAATTTGCGAGGGATGTTTTCCATCGCAAATTTCCCTCGCAAATCGCCT

Ler-0 CAACTCTGTAGCAATTTGCGAGGGATGTTTTCCATCGCAAATTTCCCTCGCAAATCGCCT

************************************************************

NCBI GTTTTCTTGTAGCGAAAAAGAAAAAATCTAAACTATTTGCTTCATCATCGCTCTTCCTAC

Ler-0 GTTTTCTTGTAGCGAAAAAGAAAAAATCTAAACTATTTGCTTCATCATCGCTCTTCCTAC

************************************************************

NCBI AAAACAACCTGGCTTAAAAAAAGGTGTCATTGTTTTCATAATGTGTTATTTTATCATTAT

Ler-0 AAAACAACCTGGCTTAAAAAAAGGTGTCATTGTTTTCATAATGTGTTATTTTATCATTAT

************************************************************

NCBI TTGTCCATCTATATTTTTATATTTTGCTAAAACTTGTATCTTTTGATATCATTTACATGG

Ler-0 TTGTCCATCTATATTTTTATATTTTGCTAAAACTTGTATCTTTTGATATCATTTACATGG

************************************************************

NCBI ATTTCACTTGTTGGACACATAGAAGTGTAGAGAAATAATTTGCATTCCTTGATTTAGTGG

Ler-0 ATTTCACTTGTTGGACACATAGAAGTGTAGAGAAATAATTTGCATCCCTTGATTTAGTGG

********************************************* **************

NCBI ACATGATATTGTGGAACTCAATTTGCGAGGGAACTCAGTTTCCCGCAAAGCCCTCGCTAA

Ler-0 ACATGATATTGTGGAACTCAATTTGCGAGGGAACTCAGCTTCCCGCAAAGCCCTCGCTAA

************************************** *********************

NCBI TTTGCGACGTTTTTGCGGGGAATCATGACTTCCACGCAGTTTCCTCGCAATTGTCTCACA

Ler-0 TTTGCGACGTTTTTGCGGGGAATCATGACTTCCACGCAGTTTCCTCGCAATTGTCTCACA

************************************************************

NCBI AATTTGTGAGGAAAATATTCGTCACAAAGTTTGCGACAGAATATCGACTTGGTCCTGTTC

Ler-0 AATTTGTGAGGAAAATATTCGTCACAAAGTTTGCGACAGAATATCGACTTGGTCCTGTTC

************************************************************

NCBI CCTCACAATCCTCTCGCAACTCTGTAGTAATTTGAGTGATGTTTTCCATCGCAAATTTCT

Ler-0 CCTCACAATCCTCTCGCAACTCTGTAGTAATTTGAGTGATGTTTTCCATCGCAAATTTCT

************************************************************

NCBI CTCGCAAATCGCCTGTTTTCTTGTAGTGAAAAAGAAAAAATCTAAACTATTTGCTTCATC

Ler-0 CTCGCAAATCGCCTGTTTTCTTGTAGTGAAAAAGAAAAAATCTAAACTATTTGCTTCATC

************************************************************

NCBI ATCGCTCTTGCTACAAACCAAACTGGCTTGAAAAAAGATGTCATTGTTTTCATAATGTGT

Ler-0 ATCGCTCTTGCTACAAACCAAACTGGCTTGAAAAAAGATGTCATTGTTTTCATAATGTGT

************************************************************

NCBI TATCCATCATTATATGTCCATCTATATGTTTATATTTTTCTAAAACTTGTATCTTTTGAT

Ler-0 TATCCATCATTATATGTCCATCTATATGTTTATATTTTTCTAAAACTTGTATCTTTTGAT

************************************************************

NCBI ATCATTTACATGGATTTCACTTGTTGGACACATAGAAGTGTAGAGAAATAATTTGCATTC

Ler-0 ATCATTTACATGGATTTCACTTGTTGGACACATAGAAGTGTAGAGAAATAATTTGCATTC

************************************************************

NCBI CTTGATTTAGTGGACATGATATTGTGGATACTATCTAACTTGCAGAAACTATTTCTTATA

Ler-0 CTTGATTTAGTGGACATGATATTGTGGATACTATCTAACTTGCAGAAACTATTTCTTATA

************************************************************

NCBI TTTTTTCAGACCAGTTTCTTGTTTAAAGGGGATCAAGTCAAACCACGGCATGGACTCGGT

Ler-0 TTTTTTCAGACCAGTTTCTTGTTTAAAGGGGATCAAGTCAAACCACGGCATGGACTCGGT

************************************************************

NCBI CAATACATTCTCTCACTGGAACATTAAAGACATAAACACCATTTAGATTATGAAGATTAT

Ler-0 CAATACATTCTCTCACTGGAACATTAAAGACATAAACACCATTTAGATTATGAAGATTAT

************************************************************

NCBI TTTTTTACGCTACAAGTGACAACAAAATTGTTAGCAAATAAAGAAGTAATGACCCCCAAA

Ler-0 TTTTTTACGCTACAAGTGACAACAAAATTGTTAGCAAATAAAGAAGTAATGACCCCCAAA

************************************************************

NCBI AAAAGAAAGAAAACAATACAACACGTTTCATCGAGAAACTTACAGTGTTAAAGAAACATA

Ler-0 AAAAGAAAGAAAACAATACAACACGTTTCATCGAGAAACTTACAGTGTTAAAGAAACATA

************************************************************

NCBI TAGCTATTTCATCATTTGACTGATTGATAATATGAGTTTTGAGTGACTGAGCAATGCAAC

Ler-0 TAGCTATTTCATCATTTGACTGATTGATAATATGAGTTTTGAGTGACTGAGCAATGCAAC

************************************************************

NCBI TAACATCAATGTG-AAAATGA-TTTTTTGTTTGTCTTCTTTCTCTTGTCAAAAATCAATA

Ler-0 TAACATCAATGTGAAAAATGATTTTTTTGTTTGTCTTCTTTCTCTTGTCAAAAATCAATA

************* ******* **************************************

NCBI TAATACAAAAGGGCAAGTAGAGCTGAACATTTTGGGAGAAGCATCAATTAGATAGACTAC

Ler-0 TAATACAAAAGGGCAAGTAGAGCTGAACATTTTGGGAGAAGCATCAATTAGATAGACTAC

************************************************************

NCBI AAATTCTTTGGAATTGTTTGGAAGCCTCTTTTTCCTGTTGCACCAAAAGATTCATCACTA

Ler-0 AAATTCTTTGGAATTGTTTGGAAGCCTCTTTTTCCTGTTGCACCAAAAGATTCATCACTA

************************************************************

NCBI ACCAATACTAATCAATGTTTCATTTCTTTTTTGGCTTTCACTAAAAAATCCAAGAAGAAA

Ler-0 ACCAATACTAATCAATGTTTCATTTCTTTTTTGGCTTTCACTAAAAAATCCAAGAAGAAA

************************************************************

NCBI AAAAGGTCAAAACTGAAAGATATCATTTACATCAAACATAATCAATATAACCAGTTAAGC

Ler-0 AAAAGGTCAAAACTGAAAGATATCATTTACATCAAACATAATCAATATAACCAGTTAAGC

************************************************************

NCBI AATTAATGATACAGAAGACTCATCAATGGACAACTATGATATCATTTACATGAATTTGAC

Ler-0 AATTAATGATACAGAAGACTCATCAATGGACAACTATGATATCATTTACATGAATTTGAC

************************************************************

NCBI TTGTTGGACACACAGAAGTGGACAACAATAGTTTGCAATCCTTGATTTAGTGGACATGAT

Ler-0 TTGTTGGACACACAGAAGTGGACAACAATAGTTTGCAATCCTTGATTTAGTGGACATGAT

************************************************************

NCBI ATTGTTGATACTATGTAATTTGCATAAACTATTTTTTTTATTTGGGTGGATATAAGGACA

Ler-0 ATTGTTGATACTATGTAATTTGCATAAACTATTTTTTTTATTTGGGTGGATATAAGGACA

************************************************************

NCBI ACTATGATATCATTTACATGGATTTGACTTGTTGGACACATAGAAGTGGACAACAATAGT

Ler-0 ACTATGATATCATTTACATGGATTTGACTTGTTGGACACATAGAAGTGGACAACAATAGT

************************************************************

NCBI TTGCAGTCCTTGATTTAGTGGACATGATATTGTGCATACTATCTAATTTGCATAAACTAT

Ler-0 TTGCAGTCCTTGATTTAGTGGACATGATATTGTGCATACTATCTAATTTGCATAAACTAT

************************************************************

NCBI TTCTTCTATTTTTTTAGATCAATTTTTTGTTTAATGGGGATCAAGTCAAACCACAGCATG

Ler-0 TTCTTCTATTTTTTTAGATCAATTTTTTGTTTAATGGGGATCAAGTCAAGCCACAGCATG

************************************************* **********

NCBI GACTCTACTATTGTTGATAAAAATTTTCATTTTTAGAAAGCAACATGACTTTTCCTTTCT

Ler-0 GACTCTACTATTGTTGATAAAAATTTTCATTTTTAGAAAGCAACATGACTTTTCCTTTCT

************************************************************

NCBI CAATTCTAGCCCATCATGATTTATGTACATAGATATCTTAT

Ler-0 CAATTCTAGCCCATCATGATTTATGTACATAGATATCTTAT

*****************************************

(d) Alignment high-speed rail model sequences of Col-0_seq and NCBI.

NCBI GCTGAGTATCGGGCAATGGCTGTTG-CTACTAAGGAACTCAAATGGGTTGGTTATCTCTT

Col-0_exp GCTGAGTATCGGGCAATGGCTGTTGCCTACTAAGGAACTCAAATGGGTTGGTTATCTCTT

************************* **********************************

NCBI GACAGCTCTTCCTTTCAAAGCATCGGCTTACTTATACTGTGATAATGAAGCAGTCTTGCA

Col-0_exp GACAGCTCTTCCTTTCAAAGCATCGGCTTACTTATACTGTGATAATGAAGCAGTCTTGCA

************************************************************

NCBI CATTGCTAGCAATTTTGTGTTTCACGAGCGAACTAAACACTAGAGCGTGATTGTCACAAG

Col-0_exp CATTGCTAGCAATTTTGTGTTTCACGAGCGAACTAAACACTAGAGCGTGATTGTCACAAG

************************************************************

NCBI GTAAGAGAATGTATTGAAGCTGGGATTCTCAAGACTATGTTTGTTCGATCAGATAATCAG

Col-0_exp GTAAGAGAATGTATTGAAGCTGGGATTCTCAAGACTATGTTTGTTCGATCAGATAATCAG

************************************************************

NCBI CTTGCAGACATGTTTACTAAACCTCTTTATCCGGTGCTCTTTAGAACTAACAATAGCAAG

Col-0_exp CTTGCAGACATGTTTACTAAACCTCTTTATCCGGTGCTCTTTAGAACTAACAATAGCAAG

************************************************************

NCBI CTTGGAGTTCTTAACATCTTTGAAACTCAAGCTTGAGGGGGCTATTAGACTGTATATATA

Col-0_exp CTTGGAGTTCTTAACATCTTTGAAACTCAAGCTTGAGGGGGCTATTAGACTGTATATATA

************************************************************

NCBI GATTCGGTTTAGACCGGTTAACTTAGATTGTATTTACAATGTATATGGTTTAGATTGGTT

Col-0_exp GATTCGGTTTAGACCGGTTAACTTAGATTGTATTTACAATGTATATGGTTTAGATTGGTT

************************************************************

NCBI TAGTGTAGAGTATATAAACATCCAATTGCACATTCTGTTTCGATTGAGAAATATAGAAAA

Col-0_exp TAGTGTAGAGTATATAAACATCCAATTGCACATTCTGTTTCGATTGAGAAATATAGAAAA

************************************************************

NCBI CTTTCACATATCTCTTTGATTCA-TGTTCTCTATCAGTATTTGCTTGCAAAGTCGAAATT

Col-0_exp CTTTCACATATCTCTTTGATTCAGTGTTCTCTATCAGTATTTGCTTGCAAAGTCGAAATT

*********************** ************************************

NCBI ATTTCAACACTTTTACCTTAACGGTTTTGTCATCGTACATGATCCAACAATCCACATATA

Col-0_exp ATTTCAACACTTTTACCTTAACGGTTTTGTCATCGTACATGATCCAACAATCCACATATA

************************************************************

NCBI TAACTCAACTGAAATGGAATTAGGGCATCAATTGGATAAAGAAAACTATGAGTACAGATT

Col-0_exp TAACTCAACTGAAATGGAATTAGGGCATCAATTGGATAAAGAAAACTATGAGTACAGATT

************************************************************

NCBI TATATCCCCAATGAATACATGAAGGAACCATGGTGAAATATCATTGAGGATCTCGTTCCT

Col-0_exp TATATCCCCAATGAATACATGAAGGAACCATGGTGAAATATCATTGAGGATCTCGTTCCT

************************************************************

NCBI ATTTCTGGCTTACTTCCGGATGCA-CTAACCTTTCCAACTCATGCGTTCACTTGATCACC

Col-0_exp ATTTCTGGCTTACTTCCGGATGCACCTAACCTTTCCAACTCATGCGTTCACTTGATCACC

************************ ***********************************

NCBI TCTTCTCTTGATTTGGTCAGCATGAAGATCTTGTTTATCACCATGCTCTGTGATTTTTCA

Col-0_exp TCTTCTCTTGATTTGGTCAGCATGAAGATCTTGTTTATCACCATGCTCTGTGATTTTTCA

************************************************************

NCBI TAACCACACAGAAGATAATGAATCAAATTATGTAAGACCGAAGGAAATGAAAGTAGCTTC

Col-0_exp TAACCACACAGAAGATAATGAATCAAATTATGTAAGACCGAAGGAAATGAAAGTAGCTTC

************************************************************

NCBI AAACCGAAGGAAATGAGAAGAATGAATGCTTGAGAAGCGTACCTAAATTCTGCATCAAAA

Col-0_exp AAACCGAAGGAAATGAGAAGAATGAATGCTTGAGAAGCGTACCTAAATTCTGCATCAAAA

************************************************************

NCBI AGGACAATTGTTCATCAAAAAGGTTAGGGAAAAACCTTCGGTGATGCACTTAAGGGAGAT

Col-0_exp AGGACAATTGTTCATCAAAAAGGTTAGGGAAAAACCTTCGGTGATGCACTTAAGGGAGAT

************************************************************

NCBI GTGGAGAGGTGTCGTTTATGAGATAAATGGTAGGAAGGGGAAACGATTGAACTTTCTTGG

Col-0_exp GTGGAGAGGTGTCGTTTATGAGATAAATGGTAGGAAGGGGAAACGATTGAACTTTCTTGG

************************************************************

NCBI ACATTATTGAAAACCCTAATTTCTTAGACTAAAGATTCTA-TGTCGAGAGAAGTCTAAA-

Col-0_exp ACATTATTGAAAACCCTAATTTCTTAGACTAAAGATTCTAGTGTCGAGAGAAGTCTAAAG

**************************************** ******************

NCBI AGTTAATGACGTGGCTGTACTTGGGAAGAATTTTGAGTTAGAAAGAGAGTAATATGACGT

Col-0_exp AGTTAATGACGTGGCTGTACTTGGGAAGAATTTTGAGTTAGAAAGAGAGTAATATGACGT

************************************************************

NCBI GGCTTTTTCATTAAGTTAACTCAATAATACATAAATATTGTTTCATGTCGTTAATTTACT

Col-0_exp GGCTTTTTCATTAAGTTAACTCAATAATACATAAATATTGTTTCATGTCGTTAATTTACT

************************************************************

NCBI GAATGTATAATTATGAATTTGCTGATGTGGCATGATTTTAGGTAGTCTTGAATTTGCTGA

Col-0_exp GAATGTATAATTATGAATTTGCTGATGTGGCATGATTTTAGGTAGTCTTGAATTTGCTGA

************************************************************

NCBI TGTAGATGGTTTGAGAAGCCACAATGAGCTTCTTTTATTAGTAAGAGATTGGTTTAGAAA

Col-0_exp TGTAGATGGTTTGAGAAGCCACAATGAGCTTCTTTTATTAGTAAGAGATTGGTTTAGAAA

************************************************************

NCBI TATTTTTAAAGAGAAATTTCTAACAAAATCTTACATGTATGTTTCTTATCTCCTACCGCT

Col-0_exp TATTTTTAAAGAGAAATTTCTAACAAAATCTTACATGTATGTTTCTTATCTCCTACCGCT

************************************************************

NCBI ACCACCACTTTCCTAGATTTATTAGCACGTGACACTTTTAGGATACAAGGAATTGAACAT

Col-0_exp ACCACCACTTTCCTAGATTTATTAGCACGTGACACTTTTAGGATACAAGGAATTGAACAT

************************************************************

NCBI GCATTTGTTAGAACCGTTGAATAACAATTCATAAATTATTTTTAAGTACTACATAATTTG

Col-0_exp GCATTTGTTAGAACCGTTGAATAACAATTCATAAATTATTTTTAAGTACTACATAATTTG

************************************************************

NCBI TTTCCAATGGGAAAAGTAATTAAAAAAAAGAAAAAATAG-AAAAAGGCGCCATTAAAAGT

Col-0_exp TTTCCAATGGGAAAAGTAATTAAAAAAAAGAAAAAATAGAAAAAAGGCGCCATTAAAAGT

*************************************** ********************

NCBI AGTAACCAACATATACTTTAACCGATGGCTCAAAAACTTCGTTCTCTTAACAGGTGTGGG

Col-0_exp AGTAACCAACATATACTTTAACCGATGGCTCAAAAACTTCGTTCTCTTAACAGGTGTGGG

************************************************************

NCBI TTTCCTCGCAATTGTCTTGCAAATTTGCGAGGGAAATATTCGTCGCAAAGTTTGCGACGG

Col-0_exp TTTCCTCGCAATTGTCTTGCAAATTTGCGAGGGAAATATTCGTCGCAAAGTTTGCGACGG

************************************************************

NCBI AATAGCGAGTTGGTCCTGTTCTTTCGGAGAGATCTATTCGTCGGCGAAGAAGAGGTTTGT

Col-0_exp AATAGCGAGTTGGTCCTGTTCTTTCGGAGAGATCTATTCGTCGGCGAAGAAGAGGTTTGT

************************************************************

NCBI CGGAGAGATCTATTCGTCGGCGAAGAAGAGGTTTGTCGGAGAGATCTATTCGTCGGCGAA

Col-0_exp CGGAGAGATCTATTCGTCGGCGAAGAAGAGGTTTGTCGGAGAGATCTATTCGTCGGCGAA

************************************************************

NCBI TTTGAGGTTTGTCGGAGAGAACAATTCATCGGCGAATTTGAGTTTTATCGGAGAGAACAA

Col-0_exp TTTGAGGTTTGTCAGAGAGAACAATTCATCGGCGAATTTGAGTTTTATCGGAGAGAACAA

************* **********************************************

NCBI TTCGTCGGTGAAAACGAAGAGGTTTGTCAAAATGAAACTGAAACCTTAATTTTTCAGTAA

Col-0_exp TTCGTTGGTGAAAACGAAGAGGTTTGTCAAAATGAAACTGAAACCTTAATTTTTCAGTAA

***** ******************************************************

NCBI GGTTAAGACCGTTATTTACCACATTAGAAGGGGCAGTTTTAAAAATGGCCATCAGGAAAG

Col-0_exp GGTTAAGACCGTTATTTACCACATTAGAA-GGGCAGTTTTAAAAATGGCCATCAGGAAAG

***************************** ******************************

NCBI GGCATTGTTGCAAAAGGTTATTAGAAAAGGCACTTTTGCAAATCTCCCTTTTGATTGTTA

Col-0_exp GGCATTGTTGCAAAAGGTTATTAGAAAAGGCACTTTTGCAAATCTCCCTTTTGATTGTTA

************************************************************

NCBI TATATAAGATTTCACCTGTA-CAATATCATCCCCTATTAATATCGACCTAAATCATTCTA

Col-0_exp TATATAAGATTTCACCTGTATCAATATCATCCCCTATTAATATCGACCTAAATCATTCTA

******************** ***************************************

NCBI ACCATATAACCCCGTCCTATGAAATTAAACATTATTTTGTATTAGTTTTATATAAACTTC

Col-0_exp ACCATATAACCCCGTCCTATGAAATTAAACACTATTTTGTATTAGTTTTATATAAACTTC

******************************* ****************************

NCBI AAATATTTATAATAATAAAACTAACGAATGTTACTGTAAATGATAACCAAAACATTAAAC

Col-0_exp AAATATTTATAATAATAAAACTAACGAATGTTACTGTAAATGATAACCAAAACATTAAAC

************************************************************

NCBI AAATAGTAAGGATATTTAAAACGTGTTAACCTAACCCGAGAAGACTCGCAACTCCGTCGC

Col-0_exp AAATAGTAAGGATATTTAAAACGTGTTAACCTAACCCGAGAAGACTCGCAACTCCGTCGC

************************************************************

NCBI AAAAATTGCTACAGAATAAGTTATCGTCGCAAGTGCGTCGCAATTTGCGGGGGAACTCAG

Col-0_exp AAAAATTGCTACAGAATAAGTTATCGTCGCAAGTGCGTCGCAATTTGCGGGGG-ACTCAG

***************************************************** ******

NCBI TTTCCCGCAAAGCCCTCGCAGACTTGCGACGTTTTGCGGGGAATCATGGCTTCTACGCAG

Col-0_exp TTTCCCGCAAAGCCCTCGCAGACTTGCGACGTTTTGCGGGGAATCATGGCTTCTACGCAG

************************************************************

NCBI TTTCCTCGCAATTGTCTCGCAAATTTGCGAGGGATATACTCGTCGTAAAGTTCGCGACGG

Col-0_exp TTTCCTCGCAATTGTCTCGCAAATTTGCGAGGGATATACTCGTCGTAAAGTTCGCGACGG

************************************************************

NCBI AATAGCGAGTTGGTCCTGTTCTGTCGGAGAGATCTATTTGTCGGCCAAGAAGAGGTTTGT

Col-0_exp AATAGCGAGTTGGTCCTGTTCTGTCGGAGAGATCTATTTGTCGGCCAAGAAGAGGTTTGT

************************************************************

NCBI TGGAGAGATCTATTCGTTGGCGAAGTTGAGGTTTGTCGGAGAGAACAATTTGTCGACGAA

Col-0_exp TGGAGAGATCTATTCGTTGGCGAAGTTGAGGTTTGTCGGAGAGAACAATTTGTCGACGAA

************************************************************

NCBI TTTAAGTTTTATTGGAGAGAACAATTCGTCGGCGAAAACGGAGAGGTTTGTCAAAATGAA

Col-0_exp TTTAAGTTTTATTGGAGAGAACAATTCGTCGGCGAAAACGGAGAGGTTTGTCAAAATGAA

************************************************************

NCBI AGTGAAACCCTAATTATTCAGTAAGGGTAAGACCGTTATTTTCCACATTAGAAGGGGCAA

Col-0_exp AGTGAAACCCTAATTATTCAGTAAGGGTAAGACCGTTATTTTCCACATTAGAAGGGGCAA

************************************************************

NCBI TTTTAAAAATGGCTGGCATTGTTGCAAAAGGTTATTAGAAATTGGCACTTTTGCAAATCT

Col-0_exp TTTTAAAAATGGCTGGCATTGTTGCAAAAGGTTATTAGAAATTGGCACTTTTGCAAATCT

************************************************************

NCBI CCCTTTTGATTGTTATATCTAAGATTTCACCTGTAGAATATCATCCCCTATTAATATCGA

Col-0_exp CCCTTTTGATTGTTATATCTAAGATTTCACCTGTAGAATATCATCCCCTATTAATATCGA

************************************************************

NCBI CCCAAATTATTCTGTCCATATAACCCTGTCCTATGAAATTAAACATCATTTTGTCTTAGT

Col-0_exp CCCAAATTATTCTGTCCATATAACCCTGTCCTATGAAATTAAACATCATTTTGTCTTAGT

************************************************************

NCBI TTTATATAAACTTCAAATATTTATAATAAGAGAACTAACGAATGTTACCGTAAATGATAA

Col-0_exp TTTATATAAACTTCAAATATTTATAATAAGAGAACTAACGAATGTTACCGTAAATGATAA

************************************************************

NCBI CCAAAACATAAAACAAATAGTAAAAAAATTTAAATTGTGTTCACCTAACCCGCGAAGACT

Col-0_exp CCAAAACATAAAACAAATAGTAAAAAAATTTAAATTGTGTTCACCTAACCCGCGAAGACT

************************************************************

NCBI CGCAACTCCATCGGAAAATTTGCTAGAGAATAAATTATCGTCGCAAGTGCGTCACAATTT

Col-0_exp CGCAACTCCATCGGAAAATTTGCTAGAGAATAAATTATCGTCGCAAGTGCGTCACAATTT

************************************************************

NCBI GCGGGGGAACTCTGTTTCCCGCAAAGCCCTCGCAAATTTGCGACGTTTTTGCGGGAATCA

Col-0_exp GCGGGGGAACTCTGTTTCCCGCAAAGCCCTCGCAAATTTGCGACGTTTTTGCGGGAATCA

************************************************************

NCBI TGACTTCCACGCAGTTTCCTTGC-AATTGTCTTGCAAATTTGCGAAGGAAATATACGTCG

Col-0_exp TGACTTCCACGCAGTTTCCTTGCGAATTGTCTTGCAAATTTGCGAAGGAAATATACGTCG

*********************** ************************************

NCBI CAAAGTTTGCGACGGAATATCGAGTTGGTCATGTTCCCTCGGAGAGATTCGTCGGCGAAG

Col-0_exp CAAAGTTTGCGACGGAATATCGAGTTGGTCATGTTCCCTCGGAGAGATTCGTCGGCGAAG

************************************************************

NCBI AAGAGGTTTGTCGGAGAGATCTATTCGTCGGCGAATTTGAGGTTTGTCAGGGAGAAAACA

Col-0_exp AAGAGGTTTGTCGGAGAGATCTATTCGTCGGCGAATTTGAGGTTTGTCAGGGAGAAAACA

************************************************************

NCBI ATTCGTCGGCGAATTTGAGTTTTATCAGAGAGAACAATTTGTCGACGAATTTGAGTAGGT

Col-0_exp ATTCGTCGGCGAATTTGAGTTTTATCAGAGAGAACAATTTGTCGACGAATTTGAGTAGGT

************************************************************

NCBI TTGTCAAAATGAGAGTGAAACCCTAATTATTCAGTAAGGGTAAAACCATTATTTTCCACA

Col-0_exp TTGTCAAAATGAGAGTGAAACCCTAATTATTCAGTAAGGGTAAAACCATTATTTTCCACA

************************************************************

NCBI TTAGAAAGGGCAGTTTTAAAAATGGCCATCAGAAAAGGGCATTGTTGCAAAAGGTTATTA

Col-0_exp TTAGAAAGGGCAGTTTTAAAAATGGCCATCAGAAAAGGGCATTGTTGCAAAAGGTTATTA

************************************************************

NCBI CAAAAATGGCACTTTTGCAAATCTCCCTTTTGATTGTTATATCTAAGATTTCACCTCTAC

Col-0_exp CAAAAATGGCACTTTTGCAAATCTCCCTTTTGATTGTTATATCTAAGATTTCACCTCTAC

************************************************************

NCBI AATATCATCCCCTATTAATATCGACCCAAATCATTCTGACCATATAACCCCGTCCTATGA

Col-0_exp AATATCATCCCCTATTAATATCGACCCAAATCATTCTGACCATATAACCCCGTCCTATGA

************************************************************

NCBI AATTAAACATTATT-TTGTCTTAGTTTTATATAAACTTCAAATATTTATAATAATTAAAC

Col-0_exp AATTAAGCATTATTCTTGTCTTAGTTTTATATAAACTTCAAATATTTATAATAATTAAAC

****** ******* *********************************************

NCBI TAACGAATGTTACTGTAAATGATAACCAAAACATTAAACAAATAGTAATGAAATTTAAAA

Col-0_exp TAACGAATGTTACTGTAAATGATAACCAAGACATTAAACAAATAGTAATGAAATTTAAAA

***************************** ******************************

NCBI CGTGTTCACCTAACCTGCGAATACTCGCAACTCCATCGGAAAATTCGCTACAATATAAGT

Col-0_exp CGTGTTCACCTAACCTGCGAATACTCGCAACTCCATCGGAAAATTCGCTACAATATAAGT

************************************************************

NCBI TATCGTCGCAAGTGCGTCGCAATTTGCAGGGGGTCTCAGTTTCCTCGCAATTGTCTTTCA

Col-0_exp TATCGTCGCAAGTGCGTCGCAATTTGCAGGGGGTCTCAGTTTCCTCGCAATTGTCTTTCA

************************************************************

NCBI AATTTGCGAGGGAAATATTCGTCGCAAAGTTTGCGACGGAATAGCGAGTTGGTCCTGTTC

Col-0_exp AATTTGCGAGGGAAATATTCGTCGCAAAGTTTGCGACGGAATAGCGAGTTGGTCCTGTTC

************************************************************

NCBI CCTCGAAGAGATCTATTCGCTAGCGAAGAAGAGGTTTGTCGGAGAGATCTATTCGTCGGC

Col-0_exp CCTCGAAGAGATCTATTCGCTAGCGAAGAAGAGGTTTGTCGGAGAGATC--TTCGTCGGC

************************************************* *********

NCBI GAAGAAGAGGTTTGTCGGATAGATCTATTCGTCGGCGAATTTGAGGTTTGTCGCAGAGAA

Col-0_exp GAAGAAGAGGTTTGTCGGATAGATCTATTCGTCGGCGAATTTGAGGTTTGTCGCAGAGAA

************************************************************

NCBI CAATTCGTCGGCGAATTTGAGTTTTATTGGAGAGAACAATTCGTCGGCGAAAACAAAGAG

Col-0_exp CAATTCGTCGGCGAATTTGAGTTTTATTGGAGAGAACAATTCGTCGGCGAAAACAAAGAG

************************************************************

NCBI GTTTGTCAAAATGAAAGTGAAACCCTAATTATTCAGTAGGGGTAAGACCGTTATTTTCCA

Col-0_exp GTTTGTCAAAATGAAAGTGAAACCCTAATTATTCAGTAGGGGTAAGACCGTTATTTTCCA

************************************************************

NCBI TATTCGAAGGGACAGTTTTAGAAATGGCCATCA-GGAAAGGGCATTGTTGCAAAAGGTTA

Col-0_exp TATTCGAAGGGACAGTTTTAGAAATGGCCATCAGGGAAAGGGCATTGTTGCAAAAGGTTA

********************************* **************************

NCBI TTACCAAAATGGCATTTTTGCAAATCTCCCTTTTGATTTTTATATCTATGATTTCACCTG

Col-0_exp TTACCAAAATGGCATTTTTGCAAATCTCCCTTTTGATTTTTATATCTATGATTTCACCTG

************************************************************

NCBI TAGAATATCATCCCGTATTAATATCGATCCAAATCATTCTGACCATATAACCCCGTCCTA

Col-0_exp TAGAATATCATCCCGTATTAATATCGATCCAAATCATTCTGACCATATAACCCCGTCCTA

************************************************************

NCBI TAAAATTAAACATTATTTTGTCTTAGTTTTATATAAACTTCAAATATTTATAATAAGAGA

Col-0_exp TAAAATTAAACATTATTTTGTCTTAGTTTTATATAAACTTCAAATATTTATAATAAGAGA

************************************************************

NCBI ACTAACGAATGTTACTGTAAATGATAACCAAAACATAGAACAAATAGTAAGGAAATTTAA

Col-0_exp ACTAACGAATGTTACTGTAAATGATAACCAAAACATAGAACAAATAGTAAGGAAATTTAA

************************************************************

NCBI AACGTGTTCACCTAACCCGCGAAGACTCGCAACTCCATCGGAAAATTTGCTACAGAATAA

Col-0_exp AACGTGTTCACCTAACCCGCGAAGAC-CGCAACTCCATCGGAAAATTTGCTACAGAATAA

************************** *********************************

NCBI GTTATCGTCGCAAGTGCGTCGCAATTTACGGGGAAACTCAGTTTCCCGCAAAGCCCTCGC

Col-0_exp GTTATCGTCGCAAGTGCGTCGCAATTTACGGGGAAACTCAGTTTCCCGCAAAGCCCTCGC

************************************************************

NCBI AAATTTGCCAAGTTTTTGCGGGGAATCATTGTCTCGCAAATTTGCGAGG-GAAATATTCG

Col-0_exp AAATTTGCCAAGTTTTTGCGGGGAATCATTGTCTCGCAAATTTGCGAGGCGAAATATTCG

************************************************* **********

NCBI TCTCAAAGTTTGCGATGGAATAGCGAGTTAGTCATGTTCCATCGGAGAGATCTATTCTTC

Col-0_exp TCTCAAAGTTTGCGATGGAATAGCGAGTTAGTCATGTTCCATCGGAGAGATCTATTCTTC

************************************************************

NCBI GGCGAAGAAGAGATTTGTCAGAGAGATCTATTCGTCGGCGAAGAAGAGGTTTGTCGAAGA

Col-0_exp GGCGAAGAAGAGATTTGTCAGAGAGATCTATTCGTCGGCGAAGAAGAGGTTTGTCGAAGA

************************************************************

NCBI GATCTATTCGTCGGCGAATTTGAGGTTTGTCGGAGAGAACAATTCGTTGGCGAATTTGAG

Col-0_exp GATCTATTCGTCGGCGAATTTGAGGTTTGTCGGAGAGAACAATTCGTTGGCGAATTTGAG

************************************************************

NCBI TTTTATCGGACAGAACAATTCGTCGGCGAATTTGAGTAGGTTTGTCAAAATAAAAGTGAA

Col-0_exp TTTTATCGGACAGAACAATTCGTCGGCGAATTTGAGTAGGTTTGTCAAAATAAAAGTGAA

************************************************************

NCBI ACCCTAATTATTCAGTAAAGGGTAAGACCGTTATTTTCCACGTTCGAAGGGGCAATTTTC

Col-0_exp ACCCTAATTATTCAGTAAAGGGTAAGACCGTTATTTTCCACGTTCGAAGGGGCAATTTTC

************************************************************

NCBI AAAAGGCTATCAGGAAAGGGCATTGTTGCAAAAGGTTATTAGAAAAAGGGCACTTTTGCA

Col-0_exp AAAAGGCTATCAGGAAAGGGCATTGTTGCAAAAGGTTATTAGAAAAAGGGCACTTTTGCA

************************************************************

NCBI AATCTCCCTTTTGATTGTTATATCTTAGATTTCACCTCTAGAATATCATCTCGTATTAAT

Col-0_exp AATCTCCCTTTTGATTGTTATATCTTAGATTTCACCTCTAGAATATCATCTCGTATTAAT

************************************************************

NCBI ATCGACACAAATCATTCTAACCACATAACCCCGTCCTATGAAATTAAACATCATTTTGTC

Col-0_exp ATCGACACAAATCATTCTAACCACATAACTCCGTCCTATGAAATTAAACATCATTTTGTC

***************************** ******************************

NCBI TTAGTTTTATAAACTTCAAATATTTATAATAAGACTCGCATCCTAGACCCTAACCCGCGA

Col-0_exp TTAGTTTTATAAACTTCAAATATTTATAATAAGACTCGCATCCTAGACCCTAACCCGCGA

************************************************************

NCBI AGACTCGCAACTCCGTCGCAAAAATTGCTACAGAATAAGTTATCGTCGCAAGTGCGTCGC

Col-0_exp AGACTCGCAACTCCGTCGCAAAAATTGCTACAGAATAAGTTATCGTCGCAAGTGCGTCGC

************************************************************

NCBI AATTTGC-GGGGGAACTAAGTTTCCCGCAAAGCCCTCATAAATTTGCTACGTTTTTGCGG

Col-0_exp AATTTGCGGGGGGAACTAAGTTTCCCGCAAAGCCCTCATAAATTTGCTACGTTTTTGCGG

******* ****************************************************

NCBI GGAATCATGACTTACACACAGTTTTCTCGCAATTGTCTAGCAAACGTGCGAGGGAAATAT

Col-0_exp GGAATCATGACTTACACACAGTTTTCTCGCAATTGTCTAGCAAACGTGCGAGGGAAATAT

************************************************************

NCBI TCGTCGCAAAATTTGCGACGGAATAGCGTGTTGGTCATGTTCCCTCGGAGAGATCTATTC

Col-0_exp TCGTCGCAAAATTTGCGACGGAATAGCGTGTTGGTCATGTTCCCTCGGAGGGATCTATTC

************************************************** *********

NCBI ATCGGCGAAGAAGAGGTTTGTCGGAGAGATCTATTCGTCGGCGAAGAAGAGGTTTGTCGG

Col-0_exp ATCGGCGAAGAAGAGGTTTGTCGGAGAGATCTATTCGTCGGCGAAGAAGAGGTTTGTCGG

************************************************************

NCBI AGAGATCTATTCGTCGGCACATTTGAGGTTTGCGGAGAGAACAATTCTTCGTCGAATTTG

Col-0_exp AGAGATCTATTCGTCGGCACATTTGAGGTTTGCGGAGAGAACAATTCTTCGTCGAATTTG

************************************************************

NCBI AGTTTTATCAGAGAGAATAATTCCTCGGCGAATTTGAATAGGTTTGTCAAAATGAAAGTG

Col-0_exp AGTTTTATCAGAGAGAATAATTCCTCGGCGAATTTGAATAGGTTTGTCAAAATGAAAGTG

************************************************************

NCBI AAACCCTAATTATTCAGTGAGGATAAGACCGTTATTTTCCACATTAGAAGGGACAGTTTT

Col-0_exp AAACCCTAATTATTCAGTGAGGATAAGACCGTTATTTTCCACATTAGAAGGGACAGTTTT

************************************************************

NCBI AAAAATGGCCATCAGAAAATGGCATTGTTGCAAAAGGTTATTAGAAAAAGGGCACCTTTG

Col-0_exp AAAAATGGCCATCAGAAAATGGCATTGTTGCAAAAGGTTATTAGAAAAAGGGCACCTTTG

************************************************************

NCBI CAAATCTCCCTTTTGATTGTTATATCTAAGATTTCACTTGTATAATATCATCCCGTATTA

Col-0_exp CAAATCTCCCTTTTGATTGTTATATCTAAGATTTCACTTGTATAATATCATCCCGTATTA

************************************************************

NCBI ATATCGACCCAAATCATTCTGACCATATAACCCCGTCCTATAAAATTAAACATCAGTTTG

Col-0_exp ATATCGACCCAAATCATTCTGACCATATAACCCCGTCCTATAAAATTAAACATCAGTTTG

************************************************************

NCBI TCTTAGTTTTATATAAACTTCAAATATTTATAATAAGAGAACTAA-CGAATGTTACTGTA

Col-0_exp TCTTAGTTTTATATAAACTTCAAATATTTATAATAAGAGAACTAAGCGAATGTTACTGTA

********************************************* **************

NCBI AATGATAATCAAAACATAGAACAAATAGTAAGGAAATTTAAAACGTGTTCACCTAACCCG

Col-0_exp AATGATAATCAAAACATAGAACAAATAGTAAGGAAATTTAAAACGTGTTCACCTAACCCG

************************************************************

NCBI CGAAGACTCGCAACTCCATCGGAAAATTTGCTACAGAATAAGTTATCGTCGCAAGTGCGT

Col-0_exp CGAAGACTCGCAACTCCATCGGAAAATTTGCTACAGAATAAGTTATCGTCGCAAGTGCGT

************************************************************

NCBI CGCAATTTACGGGGAAACTCAGTTTCCCGCAAAGCCCTTGCAAATTTGCCAAGTTTTTGC

Col-0_exp CGCAATTTACGGGGAAACTCAGTTTCCCGCAAAGCCCTTGCAAATTTGCCAAGTTTTTGC

************************************************************

NCBI GGGGAATCATTGTCTCGCAAATTTGCGAGGGAAATATTCGTCTCAAAGTTTGCGATGGAA

Col-0_exp GGGGAATCATTGTCTCGCAAATTTGCGAGGGAAATATTCGTCTCAAAGTTTGCGATGGAA

************************************************************

NCBI TAGCGAGTTAGTCATGTTCCATCGGAGAGATCTATTCTTCGGCGAAGAAGAGATTTGTCA

Col-0_exp TAGCGAGTTAGTCATGTTCCATCGGAGAGATCTATTCTTCGGCGAAGAAGAGATTTGTCA

************************************************************

NCBI GAGAGATCTATTCGTCGGCGAAGAAGAGGTTTGTCGAAGAGATCTATTCGTCGGCGAATT

Col-0_exp GAGAGATCTATTCGTCGGCGAAGAAGAGGTTTGTCGAAGAGATCTATTCGTCGGCGAATT

************************************************************

NCBI TGACGTTTGTCGGAGAGAACAATTCGTTGGCGAATTTGAGTTTTATCGGACAGAACAATT

Col-0_exp TGACGTTTGTCGGAGAGAACAATTCGTTGGCGAATTTGAGTTTTATCGGACAGAACAATT

************************************************************

NCBI CGTCGGCGAATTTGAGTAGGTTTGTCAAAATAAAAGTGAAACCCTAATTATTCAGTAAAG

Col-0_exp CGTCGGCGAATTTGAGTAGGTTTGTCAAAATAAAAGTGAAACCCTAATTATTCAGTAAAG

************************************************************

NCBI GGTAAGACCGTTATTTT-CCACGTTCGAAGGGGCAATTTTCAAAAGGCTATCAGGAAAGG

Col-0_exp GGTAAGACCGTTATTTTCCCACGTTCGAAGGGGCAATTTTCAAAAGGCTATCAGGAAAGG

***************** ******************************************

NCBI GCATTGTTGCAAAAGGTTATTAGAAAAAGGGCACTTTTGCAAATCTCCCTTTTGATTGTT

Col-0_exp GCATTGTTGCAAAAGGTTATTAGAAAAAGGGCACTTTTGCAAATCTCCCTTTTGATTGTT

************************************************************

NCBI ATATCTTAGATTTCACCTCTAGAATATCATCTCGTATTAATATCGACACAAATCATTCTA

Col-0_exp ATATCTTAGATTTCACCTCTAGAATATCATCTCGTATTAATATCGACACAAATCATTCTA

************************************************************

NCBI ACCATATAACCCCGTCCTATGAAATTAAACATCATTTTGTCTTAGTTTTATAAACTTCAA

Col-0_exp ACCATATAACCCCGTCCTATGAAATTAAACATCATTTTGTCTTAGTTTTATAAACTTCAA

************************************************************

NCBI ATATTTATAATAAGACTCGCATCCTAGACCCTAACCCGCGAAGACTCGCAACTCCGTCGC

Col-0_exp ATATTTATAATAAGACTCGCATCCTAGACCCTAACCCGCGAAGACTCGCAACTCCGTCGC

************************************************************

NCBI AAAAATTGCTACAGAATAAGTTATCGTCGCAAGTGTGTCGCAATTTGCGGGTGAACTCAG

Col-0_exp AAAAATTGCTACAGAATAAGTTATCGTCGCAAGTGTGTCGCAATTTGCGGGTGAACTCAG

************************************************************

NCBI TTTCCCGCAAAGCCCTCATAAATTTGCTACGTTTTTGCGGGGAATCATGACTTACACACA

Col-0_exp TTTCCCGCAAAGCCCTCATAAATTTGCTACGTTTTTGCGGGGAATCATGACTTACACACA

************************************************************

NCBI GTTTCCTCGCAATTGTCTAGCAAACGTGCGAGGGAAATATTCGTCGCAAAATTTGCGAC-

Col-0_exp GTTTCCTCGCAATTGTCTAGCAAACGTGCGAGGGAAATATTCGTCGCAAAATTTGCGACG

***********************************************************

NCBI GGAATAGCGAGTTGGTCATGTTCCCTCGGAGAGATCTATTCGTCGGCGAAGAAGAGGTTT

Col-0_exp GGAATAGCGAGTTGGTCATGTTCCCTCGGAGAGATCTATTCGTCGGCGAAGAAGAGGTTT

************************************************************

NCBI GTCGGAGAGATCTATTCGTCGGCGAAGAAGAGGTTTGTCGGAGAGATCTATTCGTCGGCA

Col-0_exp GTCGGAGAGATCTATTCGTCGGCGAAGAAGAGGTTTGTCGGAGAGATCTATTCGTCGGCA

************************************************************

NCBI AATTTGAGGTTTGCGGAGAGAACAATTCTTCGTCGAATTTGAGTTTTATCAGAGAGAATA

Col-0_exp AATTTGAGGTTTGCGGAGAGAACAATTCTTCGTCGAATTTGAGTTTTATCAGAGAGAATA

************************************************************

NCBI ATTCGTCGGCGAATTTGAGTAGGTTTGTCAAAATGAAAGTGCAACCCTAATTATTCAGTG

Col-0_exp ATTCGTCGGCGAATTTGAGTAGGTTTGTCAAAATGAAAGTGCAACCCTAATTATTCAGTG

************************************************************

NCBI AGGATAAGACCGTTATTTTCCACATTAGAAGGGGCAGTTTTAAAAATGGCCATCAGAAAA

Col-0_exp AGGATAAGACCGTTATTTTCCACATTAGAAGGGGCAGTTTTAAAAATGGCCATCAGAAAA

************************************************************

NCBI TGGCATTGTTGCAAAAGGTTATTAGAAAAATGGCACTTTTGCAAATATCCCTTTTGATTG

Col-0_exp TGGCATTGTTGCAAAAGGTTATTAGAAAAATGGCACTTTTGCAAATATCCCTTTTGATTG

************************************************************

NCBI TTATATCTAAGATTTCACCTGTAGAATATCATCCAGTATTAATATCGACCCAAATCATTC

Col-0_exp TTATATCTAAGATTTCACCTGTAGAATATCATCCAGTATTAATATCGACCCAAATCATTC

************************************************************

NCBI TAACCATATAACCCCGTCCTATGAAATTAAACATCATTTTGTCTTAGTTTTATATAAACT

Col-0_exp TAACCATATAACCCCGTCCTATGAAATTAAACATCATTTTGTCTTAGTTTTATATAAACT

************************************************************

NCBI TCAAATATTTATAATAAGAGAATTAACGAATGTTACTGTAAATGATAACCAAAACATAGA

Col-0_exp TCAAATATTTATAATAAGAGAATTAACGAATGTTACTGTAAATGATAACCAAAACATAGA

************************************************************

NCBI ACAAATAGTAAGGAAATTTAAAACGTGTTCATCTAACCCGCGAAGACTCGCAACTCCATC

Col-0_exp ACAAATAGTAAGGAAATTT-AAACGTGTTCACCTAACCCGCGAAGACTCGCAACTCCATC

******************* *********** ****************************

NCBI AAAAAATTTACCACAGAATAAGTTATCGTCGCAAGTGCGTTGCAATTTGTAGGGGAACTC

Col-0_exp AAAAAATTTACCACAGAATAAGTTATCGTCGCAAGTGCGTTGCAA-TTGTAGGGGAACTC

********************************************* **************

NCBI AGTTTCCCGCAAAGCCCTCGCAAATTTGCGACGTTTTTGCGGGGAATCATTGGCTCGCAA

Col-0_exp AGTTTCCCGCAAAGCCCTCGCAAATTTGCGACGTTTTTGCGGGGAATCATTGGCTCGCAA

************************************************************

NCBI ATTTGCGAGAGAAATATTCGTCTCAAAGTTTGCGATGGAATAGCGAGTTAGTCATGTTCC

Col-0_exp ATTTGCGAGAGAAATATTCGTCTCAAAGTTTGCGATGGAATAGCGAGTTAGTCATGTTCC

************************************************************

NCBI ATCGGAGAGATCTATTCGTCGGCAAAGAAGAGATTTGTCAGAGAGATCTATTCGTCGGCG

Col-0_exp ATCGGAGAGATCTAATCGTCGGCAAAGAAGAGATTTGTCAGAGAGATCTATTCGTCGGCG

************** *********************************************

NCBI AAGAAGAGGTTTGTCGAAGAGATCTATTCGTCGGCGAATTTGAGGTTTGTCGGAGAGAAT

Col-0_exp AAGAAGAGGTTTGTCGAAGAGATCTATTCGTCGGCGAATTTGAGGTTTGTCGGAGAGAAT

************************************************************

NCBI AATTCGTCGGCGAATTTGAGTTTTATCGGACAGAACAATTTGTCAGCGAATTTGAGTTTT

Col-0_exp AATTCGTCGGCGAATTTGAGTTTTATCGGACAGAACAATTTGTCAGCGAATTTGAGTTTT

************************************************************

NCBI ATCAGAGAGAACAATTCGTCGGCAAATTTGAGTAGGTTTGTCAAAATGAAAGTGAAACCC

Col-0_exp ATCAGAGAGAACAATTCGTCGGCAAATTTGAGTAGGTTTGTCAAAATGAAAGTGAAACCC

************************************************************

NCBI TAATTATTCAGTAAGGGTAAGACCATTATTTTCCACATTAGAAGGGGCAGTTTTAAAAAT

Col-0_exp TAATTATTCAGTAAGGGTAAGACCATTATTTTCCACATTAGAAGGGGCAGTTTTAAAAAT

************************************************************

NCBI GGCCATCAGAAAAGGGCATTGTTGCAAAAGCTTATTACAAAAATGGCACTTTTGCAAATC

Col-0_exp GGCCATCAGAAAAGGGCATTGTTGCAAAAGCTTATTACAAAAATGGCACTTTTGCAAATC

************************************************************

NCBI TCCCTTTTGATTGTTATATCTAAGATTTAATCTGTAAAATATCATCCCGTATGTATATCG

Col-0_exp TCCCTTTTGATTGTTATATCTAAGATTTAATCTGTAAAATATCATCCCGTATGTATATCG

************************************************************

NCBI ACCCAAATCATTCTGAACATATAACCCCGTCCTATGAAATTAAACATCATTTTGTCTTAG

Col-0_exp ACCCAAATCATTCTGAACATATAACCCCGTCCTATGAAATTAAACATCATTTTGTCTTAG

************************************************************

NCBI TTTTATAAACTTCAAATATTTATAATAAGACTCGCAATCCTAGACCCTAACCCGCGAAAA

Col-0_exp TTTTATAAACTTCAAATATTTATAATAAGACTCGCAATCCTAGACCCTAACCCGCGAAAA

************************************************************

NCBI CTCGCAACTCCGTCGCAAAAATTGCTACAGAATAAGTTATCGTCACAAGTGCGTCGCAAT

Col-0_exp CTCGCAACTCCGTCGCAAAAATTGCTACAGAATAAGTTATCGTCACAAGTGCGTCGCAAT

************************************************************

NCBI TTATGGGGGAACTCAGTTTCCCGCAAAGCCCTCGCAAATTTGCGACGTCTTTGCGGTGAA

Col-0_exp TTATGGGGGAACTCAGTTTCCCGCAAAGCCCTCGCAAATTTGCGACGTCTTTGCGGTGAA

************************************************************

NCBI TCATGACTTCCACGCAGTTTCCTCGCAATTGTCTCGCAAACGTGCGAGGGAAATATTCGT

Col-0_exp TCATGACTTCCACGCAGTTTCCTCGCAATTGTCTCGCAAACGTGCGAGGGAAATATTCGT

************************************************************

NCBI CGCAAAGTTTGCGACGGAATAGCGAGTTGGTCATGTTCCTTCGGAGAGATCTATTCATCG

Col-0_exp CGCAAAGTTTGCGACGGAATAGCGAGTTGGTCATGTTCCTTCGGAGAGATCTATTCATCG

************************************************************

NCBI AAGAAAAGGTTTGTCAGAGAGATCTATTCGTCGGCGAAGAAGAGGTTTGTCGGAGAGATC

Col-0_exp AAGAAAAGGTTTGTCAGAGAGATCTATTCGTCGGCGAAGAAGAGGTTTGTCGGAGAGATC

************************************************************

NCBI TATTCGTCGGCGAATTTGAGGTTTGTTGGAGAGAACAATTTGTCGGCGAATTTGAGTTTT

Col-0_exp TATTCGTCGGCGAATTTGAGGTTTGTTGGAGAGAACAATTTGTCGGCGAATTTGAGTTTT

************************************************************

NCBI ATCAGAGAGAACAATTTGTCGGCGAATTTGAGTAGGTTTGTCAAAATGAAAGTGAAACCC

Col-0_exp ATCAGAGAGAACAATTTGTCGGCGAATTTGAGTAGGTTTGTCAAAATGAAAGTGAAACCC

************************************************************

NCBI TAATTATTCAGTAAGGGTAAGACAGTTATTTTCCACATTAGAAGGGGCAGTTTTATAAAT

Col-0_exp TAATTATTCAGTAAGGGTAAGACAGTTATTTTCCACATTAGAAGGGGCAGTTTTATAAAT

************************************************************

NCBI GGCCATTCGGAAAGGGCATTGTTGCAAAATGTTATTACAAAAATGGCACTTTTGCAAATC

Col-0_exp GGCCATTCGGAAAGGGCATTGTTGCAAAATGTTATTACAAAAATGGCACTTTTGCAAATC

************************************************************

NCBI TCCCTTTTGATTGTTATATCTAAGATTTCACCTGTAGAATATCATCCAGTATTAATATCG

Col-0_exp TCCCTTTTGATTGTTATATCTAAGATTTCACCTGTAGAATATCATCCAGTATTAATATCG

************************************************************

NCBI ACCCAAATCATTCTAACCATATAACCCCGTCCTATGAAATTAAACATCATTTTGTCTTAG

Col-0_exp ACCCAAATCATTCTAACCATATAACCCCGTCCTATGAAATTAAACATCATTTTGTCTTAG

************************************************************

NCBI TTTTATATAAACTTCAAATATTTATAATAAGAGAATTAACGAATGTTACTGTAAATGATA

Col-0_exp TTTTATATAAACTTCAAATATTTATAATAAGAGAATTAACGAATGTTACTGTAAATGAT-

***********************************************************

NCBI ACCAAAACATAGAACAAATAGTAAGGAAATTTAAAACGTGTTCATCTAACCCGCGAAGAC

Col-0_exp ACCAAAACATAGAACAAATAGTAAGGAAATTTAAAACGTGTTCATCTAACCCGCGAAGAC

************************************************************

NCBI TCGCAACTCCATCAAAAAATTTACCACAGAATAAGTTATCGTCGCAAGTGCGTTGCAATT

Col-0_exp TCGCAACTCCATCAAAAAATTTACCACAGAATAAGTTATCGTCGCAAGTGCGTTGCAATT

************************************************************

NCBI TGTAGGGGAACTCAGTTTCCCGCAAAGCCCTCGCAAATTTGCGACGTTTTTGCGGGGAAT

Col-0_exp TGTAGGGGAACTCAGTTTCCCGCAAAGCCCTCGCAAATTTGCGACGTTTTTGCGGGGAAT

************************************************************

NCBI CATTGGCTCGCAAATTTGTGAGGGAAATATTTGTCTCAAAGTTTGCGACGGAATAACGAG

Col-0_exp CATTGGCTCGCAAATTTGTGAGGGAAATATTTGTCTCAAAGTTTGCGACGGAATAACGAG

************************************************************

NCBI TTGGTCATGTTCCCTCAGAGAGATCTATTCGTCGGCGAAGAAGAGGTTTGTCGGAGAGAT

Col-0_exp TTGGTCATGTTCCCTCAGAGAGATCTATTCGTCGGCGAAGAAGAGGTTTGTCGGAGAGAT

************************************************************

NCBI CTATTCGTCGACGAAGAAGAGGTTTGTCGGAGAGATCTATTCGTCGGCGAATTTGAGGTT

Col-0_exp CTATTCGTCGACGAAGAAGAGGTTTGTCGGAGAGATCTATTCGTCGGCGAATTTGAGGTT

************************************************************

NCBI TGTCGGAGAGAACAATTCGTCGGCGAATTTGAGTTTTATCGGAGAGAACAATTCGTCGGC

Col-0_exp TGTCGGAGAGAACAATTCGTCGGCGAATTTGGGTTTTATCGGAGAGAACAATTCGTCGGC

******************************* ****************************

NCBI GAATTTGAGTAGGTTTGTCAAAATGAAAGTGAAACCCTAATTATTCAGTAAAGGGATAGA

Col-0_exp GAATTTGAGTAGGTTTGTCAAAATGAAAGTGAAACCCTAATTATTCAGTAAAGGGATAGA

************************************************************

NCBI CCGTTATTTTCCACATTAGAAGGGCAATTTTCAAAATGGCTATCAGGAAAGGGCATTGTT

Col-0_exp CCGTTATTTTCCACATTAGAAGGGCAATTTTCAAAATGGCTATCAGGAAAGGGCATTGTT

************************************************************

NCBI GGAAAAGGTAATTAGAAAAAGGGCACTTTTGCAAATCTCCTTTTTGATTGTTATACCTTA

Col-0_exp GGAAAAGGTAATTAGAAAAAGGGCACTTTTGCAAATCTCCTTTTTGATTGTTATACCTTA

************************************************************

NCBI GATTTCACCTGTAGAATATCATCTCGTATTAATATCGACCCAAATCATTCTAACCATATA

Col-0_exp GATTTCACCTGTAGAATATCATCTCGTATTAATATCGACCCAAATCATTCTAACCATATA

************************************************************

NCBI ACCCCGTCCTATGAAATTAAACATCATTTTGTCTTAGTTTTCTAAACTTTAAATATTTAT

Col-0_exp ACCCCGTCCTATGAAATTAAACATCATTTTGTCTTAGTTTTCTAAACTTTAAATATTTAT

************************************************************

NCBI AATAATAGAACAAACGAATGTTACAATAAATGATAACCAAAACATAGAACAAATAGTAAG

Col-0_exp AATAATAGAACAAACGAATGTTACAATAAATGATAACCAAAACATAGAACAAATAGTAAG

************************************************************

NCBI GAAATTTATAACGTGTTCACCTAACCCGCGAAGACTCGCAACTCTGTTGCAAAATTTGCT

Col-0_exp GAAATTTATAACGTGTTCACCTAACCCGCGAAGACTCGCAACTCTGTTGCAAAATTTGCT

************************************************************

NCBI ACAGAATAAGTTATCGTCGCAAGTGCGTTGCAATTTACGGGGGAACTCAGTTTCCGCAAA

Col-0_exp ACAGAATAAGTTATCGTCGCAAGTGCGTTGCAATTTACGGGGGAACTCAGTTTCCGCAAA

************************************************************

NCBI GCCCTCGCAAATTTGCGACGTTTTTGCGGGGAATCATGACTTCCACGCAGTTTCCTCGCA

Col-0_exp GCCCTCGCAAATTTGCGACGTTTTTGCGGGGAATCATGACTTCCACGCAGTTTCCTCGCA

************************************************************

NCBI ATTGTCTCGCAAATTTGCGAGGGAAATATTTGTCGCAAAGTTTGCGATTGGTCCTGTTCC

Col-0_exp ATTGTCTCGCAAATTTGCGAGGGAAATATTTGTCGCAAAGTTTGCGATTGGT-CTGTTCC

**************************************************** *******

NCBI CTCGGAGAGATCTATTCGTCGGCGAAGAAAAGGTTTGTCGGAGAGATCTATTCGTCATTA

Col-0_exp CTCGGAGAGATCTATTCGTCGGCGAAGAAAAGG-TTGTCGGAGAGATCTATTCGTCATTA

********************************* **************************

NCBI GGAAAGGGCATTGTTGCAAAAGATTATTAGAATATCATCACGTATTAATATCGACCCAAA

Col-0_exp GGAAAGGGCATTGTTGCAAAAGATTATTAGAATATCATCACGTATTAATATCGACCCAAA

************************************************************

NCBI TCATTCTGACTATATAACCGCGTCTTATGAAATTAAATATCATTTTGTCTTAGTTTAAGA

Col-0_exp TCATTCTGACTATATAACCGCGTCTTATGAAATTAAATATCATTTTGTCTTAGTTTAAGA

************************************************************

NCBI ACTTCAAATATTTATAATAAGACTCGCAACCCTAAACCCTAACCAGC-GAAGACTCGCAA

Col-0_exp ACTTCAAATATTTATAATAAGACTCGCAACCCTAAACCCTAACCAGCAGAAGACTCGCAA

*********************************************** ************

NCBI CTCCGTCGCAAAAATTGCTACAGAATAAGTTTTCGTCGCAAGTGCGTCGCAATTTGCGTG

Col-0_exp CTCCGTCGCAAAAATTGCTACAGAATAAGTTTTCGTCGCAAGTGCGTCGCAATTTGCGTG

************************************************************

NCBI GGAACTCAGTTTCCCGCAAATCCCTTGCAAATTTGCGACGTTTTTGCGGGGAATCATGAC

Col-0_exp GGAACTCAGTTTCCCGCAAATCCCTTGCAAATTTGCGACGTTTTTGCGGGGAATCATGAC

************************************************************

NCBI TTCCACGCAGTTTCCTTGCAATTGTCTTGCAAATTTGCGAGGGAAATATTCGTCGCAATG

Col-0_exp TTCCACGCAGTTTCCTTGCAATTGTCTTGCAAATTTGCGAGGGAAATATTCGTCGCAATG

************************************************************

NCBI TTTGCGACGGAATAGCGAGTTGGTCCTGTTCCCTCGCAAACCTCTCGCAACTCTGTAACA

Col-0_exp TTTGCGACGGAATAGCGAGTTGGTCCTGTTCCCTCGCAAACCTCTCGCAACTCTGTAACA

************************************************************

NCBI ATTTGCAAGGGATGTTTTCCATCGCAAATTTCTCTCGCAAATCGCCTGTTTTCTTGTTGT

Col-0_exp ATTTGCAAGGGATGTTTTCCATCGCAAATTTCTCTCGCAAATCGCCTGTTTTCTTGTTGT

************************************************************

NCBI CAAAAAAAAAAAATCTAAACTATTTGCTTCATCATCGCTCTTCCTACAAACCAAACTGGC

Col-0_exp CAAAAAAAAAAAATCTAAACTATTTGCTTCATCATCGCTCTTCCTACAAACCAAACTGGC

************************************************************

NCBI TTGAAAAAAGCTGTCATTGTTTTCATCATGTGTTATCCATCATTATCTGTCCATCTATAT

Col-0_exp TTGAAAAAAGCTGTCA-TGTTTTCATCATGTGTTATCCATCATTATCTGTCCATCTATAT

**************** *******************************************

NCBI GTTTATATTTTGATAAAACTTGTATCTTTTGATATCATTTACATTGATTTCACTTGTTGG

Col-0_exp GTTTATATTTTGATAAAACTTGTATCTTTTGATATCATTTACATTGATTTCACTTGTTGG

************************************************************

NCBI ACACATAGAAGTGTAGAGAAATAATTTGCATTCCTTGATTTAGTGGACATAATATTGTGG

Col-0_exp ACACATAGAAGTGTAGAGAAATAATTTGCATTCCTTGATTTAGTGGACATAATATTGTGG

************************************************************

NCBI AACTCAATTTGCGAGGAACTCAGTTTCCCGCAAAGCTCTCGCAACTCTGTAGCAATTTGC

Col-0_exp AACTCAATTTGCGAGGAACTCAGTTTCCCGCAAAGCTCTCGCAACTCTGTAGCAATTTGC

************************************************************

NCBI GAGGGATGTTTTCCATCGCAAATTTCCCTCGCAAATCGCCTGTTTTCTTGTAGCGAAAAA

Col-0_exp GAGGGATGTTTTCCATCGCAAATTTCCCTCGCAAATCGCCTGTTTTCTTGTAGCGAAAAA

************************************************************

NCBI GAAAAAATCTAAACTATTTGCTTCATCATCGCTCTTCCTACAAAACAACCTGGCTTGAAA

Col-0_exp GAAAAAATCTAAACTATTTGCTTCATCATCGCTCTTCCTACAAAACAACCTGGCTTGAAA

************************************************************

NCBI AAAGGTGTCATTGTTTTCATAATGTGTTATTTTATCATTATCTGTCCATCTATATATTTA

Col-0_exp AAAGGTGTCATTGTTTTCGTAATGTGTTATTTTATCATTATCTGTCCATCTATATATTTA

****************** *****************************************

NCBI TATTTTGCTAAAACTTGTATATTTTGATATCATTTACATGGATTTCACTTGTT-GGACCC

Col-0_exp TATTTTGCTAAAACTTGTATATTTTGATATCATTTACATGGATTTCACTTGTTGGGACCC

***************************************************** ******

NCBI ATAGAAGTGTAGAGAAATAATTTGCATTCCTTGATTTAGTGGACATGATATTGTGGAACT

Col-0_exp ATAGAAGTGTAGAGAAATAATTTGCATTCCTTGATTTAGTGGACATGATATTGTGGAACT

************************************************************

NCBI CAATTTGCGAGTGAACTCAGTTTCCCGCAAAGCCCTCGCTAATTTGCGACGTTTTTGCGG

Col-0_exp CAATTTGCGAGTGAACTCAGTTTCCCGCAAAGCCCTCGCTAATTTGCGACGTTTTTGCGG

************************************************************

NCBI GGAATCATGACTTCCACGCAGTTTCCTCGCAATTGTCTCACAAATTTGTGAGGGAAATAT

Col-0_exp GGAATCATGACTTCCACGCAGTTTCCTCGCAATTGTCTCACAAATTTGTGAGGGAAATAT

************************************************************

NCBI TCGTCACAAAGTTTGCGACGGAATATCGACTTGGTCCTGTTCCCTCACAATCCTCTCGCA

Col-0_exp TCGTCACAAAGTTTGCGACGGAATATCGACTTGGTCCTGTTCCCTCACAATCCTCTCGCA

************************************************************

NCBI ACTCTGTAGCAATTTGAGTGATGTTTTCCATTGCAAATTTCTCTCGCAAATCGCCTGTTT

Col-0_exp ACTCTGTAGCAATTTGAGTGATGTTTTCCATTGCAAATTTCTCTCGCAAATCGCCTGTTT

************************************************************

NCBI TCTTGTAGTGAAAAAGAAAAAATCTAAACTATTTGCTTCATC-ATCGCTCTTGCTACAAA

Col-0_exp TCTTGTAGTGAAAAAGAAAAAATCTAAACTATTTGCTTCATCGATCGCTCTTGCTACAAA

****************************************** *****************

NCBI CCAAACTGGCTTGAAAAAAGATGTCATTGTTTTCATAATATGTTATCCATCATTATATGT

Col-0_exp CCAAACTGGCTTGAAAAAAGATGTCATTGTTTTCATAATATGTTATCCATCATTATATG-

***********************************************************

NCBI CCATCTATATGTTTATATTTTTCTAAAACTTGTATCTTTTGATATCATTTACATGGATTT

Col-0_exp CCATCTATATGTTTATATTTTTCTAAAAC-TGTATCTTTTGATATCATTTACATGGATTT

***************************** ******************************

NCBI CACTTGTTGCACACATAGAAGTGTAGAGAAATAATTTGCATTCCTTGATTTAGTGGACAT

Col-0_exp CACTTGTTGCACACATAGAAGTGTAGAGAAATAATTTGCATTCCTTGATTTAGTGGACAT

************************************************************

NCBI GATATTGTGGATACTATCTAACTTGCAGAAACTATTTCTTATATTTTTTCAGACCAGTTT

Col-0_exp GATATTGTGGATACTATCTAACTTGCAGAAACTATTTCTTATATTTTTTCAGACCAGTTT

************************************************************

NCBI CTTGTTTAAAGGGGATCAAGTCAAACCACGGCATGGACTCTTCTACGGTCAATACATTCT

Col-0_exp CTTGTTTAAAGGGGATCAAGTCAAACCACGGCATGGACTCTTCTACGGTCAATACATTCT

************************************************************

NCBI CTCTCTGGAACATTAAAGACATAAACACCATTTACATTATGAAGATTATTTTTTTACGCT

Col-0_exp CTCTCTGGAACATTAAAGACATAAACACCATTTACATTATGAAGATTATTTTTTTACGCT

************************************************************

NCBI ACAAGTGACAACAAAATTGTTAGCAAATAAAGAAGTAAT-GACCCCCAAAAAAAGAAAGA

Col-0_exp ACAAGTGACAACAAAATTGTTAGCAAATAAAGAAGTAATGGACCCCCAAAAAAAGAAAGA

*************************************** ********************

NCBI AAACAATACAACACGTTTCATCGAGAAACTTACAGTGTTAAA-GAAACATATAGCTATTT

Col-0_exp AAACAATACAACACGTTTCATCGAGAAACTTACAGTGTTAAAGGAAACATATAGCTATTT

****************************************** *****************

NCBI CATCATTTGACTGATTGATAATATGAGTTTTGAGTGA-CTGAGCAATGC-AACTAACATC

Col-0_exp CATCATTTGACTGATTGATAATATGAGTTTTGAGTGAGCTGAGCAATGCAAACTAACATC

************************************* *********** **********

NCBI AATGTGAAAATGACTTTTTGTTTGTCTTCTTTCTCTTGTTAAATATCAATATAATACAAA

Col-0_exp AATGTGAAAATGACTTTTTGTTTGTCTTCTTTCTCTTGTTAAATATCAATATAATACAAA

************************************************************

NCBI AGGGCAAGTAGAGCTGAACATTTTGGGAGAAGCATCAATTAGATAGACTACAAATTCTTT

Col-0_exp AGGGCAAGTAGAGCTGAACATTTTGGGAGAAGCATCAATTAGATAGACTACAAATTCTTT

************************************************************

NCBI GGAATTCTTTGGAAGCCTCTTTTTCCTGTTGCACCAAAAGATTCATCACTAACCAATACT

Col-0_exp GGAATTCTTTGGAAGCCTCTTTTTCCTGTTGCACC-AAAGATTCATCACTAACCAATACT

*********************************** ************************

NCBI AATCAATGTTTCATTTCTTTTTTGGCTTTCACTAAAAAGTCCAAGAAGAAAAAAAGGTTA

Col-0_exp AATCAATGTTTCATTTCTTTTTTGGCTTTCACTAAAAAGTCCAAGAAGAAAAAAAGGTTA

************************************************************

NCBI AAACTGAAAGATATCATTTACATCAAACATAATCAATATAACCGGTTAAGCAATTAATGA

Col-0_exp AAACTGAAAGATATCATTTACATCAAACATAATCAATATAACCGGTTAAGCAATTAATGA

************************************************************

NCBI TACAGAAGACTCATCAATGGACAACTATGATATCATTTACATGAATTT-GACTTGTTGGA

Col-0_exp TACAGAAGACTCATCAATGGACAACTATGATATCATTTACATGAATTTGGACTTGTTGGA

************************************************ ***********

NCBI CACACAGAAGTGGACAACAATAGTTTGCA-ATCCTTGATTTAGTGGACATGATATTGTGG

Col-0_exp CACACAGAAGTGGACAACAATAGTTTGCAGATCCTTGATTTAGTGGACATGATATTGTGG

***************************** ******************************

NCBI ATACTATGTAATTTGCAT-AAACTATTTTTTTATTTGGGTGGATATAAGGACAACTATGA

Col-0_exp ATACTATGTAATTTGCATAAAACTATTTTTTTATTTGGGTGGATATAAGGACAACTATGA

****************** *****************************************

NCBI TATCATTTACATGGATTTGACTTGTTGGACACATAGAAGTGGACAACAATAGTTTGCAGT

Col-0_exp TATCATTTACATGGATTTGACTTGTTGGACACATAGAAGTGGACAACAATAGTTTGCAGT

************************************************************

NCBI CCTTGATTTAGTGGACATGATATTGTGGATACTATCTAATTTGCATAAACTATTTCTTCT

Col-0_exp CCTTGATTTAGTGGACATGATATTGTGGATACTATCTAATTTGCATAAACTATTTCTTCT

************************************************************

NCBI ATTTTTTTAGATCAATTTCTTGTTTAATGGGGATCAAGTCAAACCACAGCATGGACTCTA

Col-0_exp ATTTTTTTAGATCAATTTCTTGTTTAATGGGGATCAAGTCAAACCACAGCATGGACTCTA

************************************************************

NCBI CTATTGTTGATAAAAATTTTCATTTTTAGAAAGCAACATGACTTTTCCTTTCTCAATTCT

Col-0_exp CTATTGTTGATAAAAATTTTCATTTTTAGAAAGCAACATGACTTTTCCTTTCTCAATTCT

************************************************************

NCBI AGCCCATTATGATTTATGTACATAGATATCTTATATTTTTTCTGCTAACGATGCAGAATG

Col-0_exp AGCCCATTATGATTTATGTACATAGATATCTTATATTTTTTCTGCTAACGATGCAGAATG

************************************************************

NCBI CAGTTGAAATGGCAACATTCATCTGATTCATGGTAAGGCCACAGTCTAAATTATGTGCAT

Col-0_exp CAGTTGAAATGGCAACATTCATCTGATTCATGGTAAGGCCACAGTCTAAATTATGTGCAT

************************************************************

NCBI CAATCCTTAAATCAGCTATGTAATGTTATTCAGAAAAAACTTTCATTTAATATTTCGGTG

Col-0_exp CAATCCTTAAATCAGCTATGTAATGTTATTCAGAAAAAACTTTCATTTAATATTTCGGTG

************************************************************

NCBI GGGAATAAAAGAAATTTTTTGCTTTATGAAGAACCATACGATGATATAAAGCCGACTAAA

Col-0_exp GGG-ATAAAAGGAATTTTTTGCTTTATGAAGAACCATACGATGATATAAAGCCGACTAAA

*** ******* ************************************************

NCBI GTCTGGGTGCGTTGGAAGTACACAATAATTTGCAGCAACACACTATCTCTAATCGTCACT

Col-0_exp GTCTGGGTGCGTTGGAAGTACACAATAATTTGCAGCAACACACTATCCCTAATCGTCACT

*********************************************** ************

NCBI CTCGTTAGTCTAAACTTTTCAGATATCTACAAGTTCCATAATCTTAAGCTTATCAAAGTC

Col-0_exp CTCGTTAGTCTAAACTTTTCAGATATCTACAAGTTCCATAATCTTAAGCTTATCAAAGTC

************************************************************

NCBI TATGATAAAACATCGCTCTGCCAGGTCAATACAAA

Col-0_exp TATGATAAAACATCGCTCTGCCAGGT-----CAAA

************************** ****

**Supplementary Figure S11.** Fragment analysis of the high-speed rail model sequences among different *Arabidopsis thaliana* ecotypes. Ler indicated Ler-0; ws indicated Ws-0; col-0 indicated Col-0; Ws-cs indicated Ws-1.

**
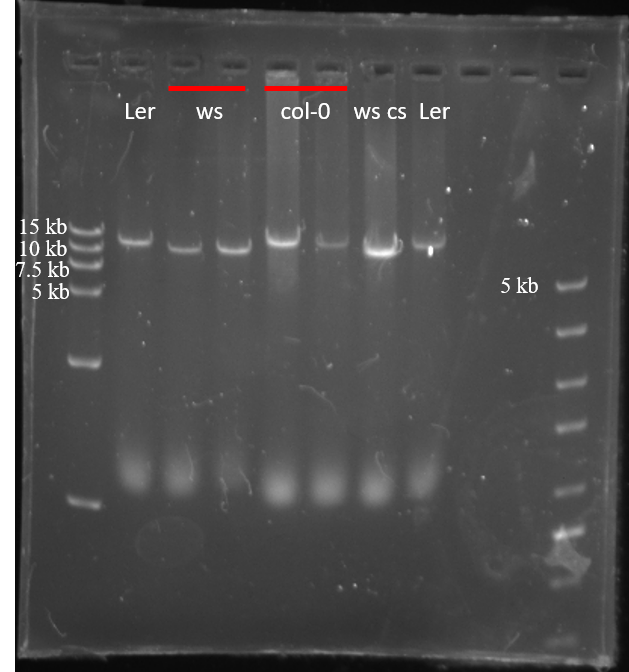
**

Gordon, D. (2003). "Viewing and editing assembled sequences using Consed." Curr Protoc Bioinformatics **Chapter 11**(2): Unit11 12.

Luo, M. C., C. Thomas, F. M. You, J. Hsiao, S. Ouyang, C. R. Buell, M. Malandro, P. E. McGuire, O. D. Anderson and J. Dvorak (2003). "High-throughput fingerprinting of bacterial artificial chromosomes using the snapshot labeling kit and sizing of restriction fragments by capillary electrophoresis." Genomics **82**(3): 378-389.
